# A freshwater resilient and connected network to sustain biodiversity under a changing climate

**DOI:** 10.1371/journal.pone.0351782

**Published:** 2026-09-16

**Authors:** Mark G. Anderson, Arlene P. Olivero, Analie R. Barnett, Mary L. Khoury, Erik H. Martin

**Affiliations:** 1 Center for Resilient Conservation Science, The Nature Conservancy, Newburyport, Massachusetts, United States of America; 2 The Nature Conservancy, Atlanta, Georgia, United States of America; 3 The Nature Conservancy, Chicago, illinois, United States of America; 4 The Nature Conservancy, Brunswick, Maine, United States of America; Universität für Bodenkultur Wien: Universitat fur Bodenkultur Wien, AUSTRIA

## Abstract

Over the last century, the integrity of streams and lakes in the U.S. has deteriorated. Our river networks are fragmented by thousands of dams hindering movement and adaptation. Water diversions, groundwater withdrawals, and habitat degradation have depleted flows and altered hydrology. Now climate change is altering precipitation patterns and temperature regimes, sending freshwater species into rapid decline. To address this crisis, The Nature Conservancy (TNC), working with 66 freshwater conservationists representing 44 states, mapped the characteristics that impart resilience to freshwater systems and identified a representative network that, if conserved and restored, could theoretically sustain the nation’s aquatic biodiversity. Key attributes of resilience included connectivity, condition, water availability, and naturalness of flow. Each attribute was assessed individually, and the results were combined into a resilience score for every small watershed (HUC-12). We overlaid the results with 91 spatial freshwater biodiversity assessments to identify a network that meets criteria for representation, resilience, connectivity, and biodiversity: what we have termed a ‘freshwater resilient and connected network.’ The network covers 35% of the conterminous U.S. and separates into 19% scoring as resilient and 16% as restorable (i.e., average resilience with one below-average factor). It includes 67% of recognized biodiversity areas, 75% of free-flowing rivers, and at least 30% of every freshwater ecoregion except one. Current conservation of the network distributes as 6% protected and resilient, 2% protected and restorable, 13% unprotected and resilient, and 14% unprotected and restorable. The results form a foundation for sustaining diverse and adaptive freshwater systems.

## Introduction

Freshwater ecosystems are vital to biodiversity and human wellbeing. Rivers, lakes, and wetlands support thousands of species that collectively purify water, cycle nutrients, maintain watershed integrity, and provide food [1]. Inland recreational fisheries destined for human consumption in the U.S. are worth an estimated $2.38 billion annually [2]. Yet the diverse freshwater biota of the U.S. is the country’s most threatened species group [3] with 72% of freshwater mussels and 39% of freshwater fish in North America being imperiled, including nearly 60% of freshwater fish in California [4–6]. Likewise, a quarter of the world’s freshwater species are threatened with extinction from pollution (54%), dams and water extraction (39%), agriculture and land use change (37%), and invasive species and disease (28%) [7].

Freshwater species are uniquely vulnerable to changes in climate because they are confined to aquatic habitats where movement to alternative habitats is typically more restricted than in terrestrial systems [8]. Climate-driven range shifts to more northern latitudes or higher altitudes have been documented for temperate fish populations [9–11], however, the 150,000 + dams in the conterminous U.S. hamper the ability of species to move and have contributed to the decline of many freshwater species, particularly migratory fish [12,13].

The Convention on Biological Diversity Target 3 calls for conservation of 30% of inland waters by 2030 [14], but to date, most area-based conservation plans are strongly focused on land and are unlikely to sustain freshwater biodiversity on their own [15,16]. To meet this challenge and support freshwater conservation efforts, we led a four-year, 48-state project to 1) assess and map the characteristics of freshwater systems that convey resilience to climate change and 2) identify a resilient and connected freshwater network that, if conserved and restored, could potentially sustain the full spectrum of aquatic biodiversity. To understand the scope of challenges ahead, we evaluated the network’s representation of species and ecosystems and assessed its current conservation status.

Given the high uncertainty associated with changes in precipitation, and the effectiveness of humanity’s collective actions [17], we do not attempt to predict how climate change will affect specific freshwater systems and only use projected stream temperature changes in the analysis due to the importance of cold-water systems. Instead, we identify places where the enduring physical conditions set the stage for aquatic species and ecosystems to adapt to changing climate conditions. This approach, known as Conserving Nature’s Stage [18], has been used successfully for mapping a climate-resilient terrestrial network [19]. Accordingly, we map the abiotic components of each freshwater network, along with current condition, to estimate its ability to sustain biological diversity even as conditions change in response to a changing climate, e.g., its “resilience” [20]. Geophysical factors act as a template that dictates environmental regimes, such as water flow and temperature, which in turn determine the suitability of a habitat for specific fish species [21,22]. However, we recognize that freshwater species are particularly sensitive to flow variation and have lower and more specialized thermal tolerances than terrestrial species [23]. We assume that by creating or maintaining the enabling conditions for freshwater systems to adapt and thrive, we can reduce some of the uncertainty of climate change impacts.

## Methods

### Approach and study area

We developed spatially explicit datasets for quantifying key aspects of freshwater climate-resilience, integrated them into a continuous resilience score, and combined them with biodiversity data to identify an ecologically representative, resilient, and connected freshwater network for CONUS. The project was guided by a steering committee of 66 TNC scientists and partners from 44 state and regional programs with expert knowledge of the geographic region they represented. With their guidance, we adjusted details of our methods and varied our weighting schemes to accommodate the wide variety of freshwater systems across the U.S. (flowchart in S1 File). We used three scales of analysis units: 1) individual stream catchments (median size 1.4 km^2^, IQR 0.4–3.1 km^2^); 2) small watersheds (HUC-12s, median size 88.2 km^2^, IQR 63.8–117.2 km^2^); and 3) Functionally Connected Networks (FCNs, median size 171.7 km^2^, IQR 88.5–457.8 km^2^). The latter refers to the contiguous portions of a river network that an aquatic organism could potentially move through before they encounter a dam, barrier, headwater source, or river mouth [24]. The length of the included stream network, and the land area draining into each FCN, was used to represent the stream channel and area influencing the surface and groundwater flows.

Depending on dam locations, FCN’s can cover thousands of hectares, or they can subdivide a HUC-12. Our premise was that an FCN’s size, diversity, condition, and water availability govern the degree to which the network provides options for species and creates the enabling conditions needed to maintain ecological function. Our methods and indices integrate across scales and deliver the results at a HUC-12 spatial resolution appropriate for informing local, regional, and national decisions. All spatial analyses were done with Esri ArcGIS Pro 3.5.2.

Climatic regions were used to inform the weighting scheme applied when combining components. We classified the study area into three climatic zones: humid (Eastern Forests and Pacific Northwest), arid (Great Plains and Columbia Plateau), and xeric (Southwest Deserts) based on an aridity index calculated as the ratio of precipitation to potential evapotranspiration (>0.65 humid, < 0.65 arid, < 0.03 xeric, Fig 2-8) [25,26]. Freshwater ecoregions [27] were assigned to one climate region based on the region’s dominant climatic zone (Fig 2A), and all HUC-12s and FCNs were assigned to their dominant freshwater ecoregion and associated climatic zone based on the proportion of the FCN area in each. In arid and xeric ecoregions, more weight was given to the water score, while in humid ecoregions more weight was given to connectivity and condition (S1 File).

### Streams and dams

To assess streams, rivers, and waterbodies, we used the 1:100,000 scale National Hydrography Dataset Plus version 2.1 (NHDPlusV2) [28]. We converted the dataset into a dendritic network, and classified streams into size classes based on total upstream drainage area following Wang et al. [29]: Headwaters (1 = < 100 km^2^) or Rivers (2 Small = < 1,000 km^2^, 3 Medium = < 10,000 km^2^, 4 Mainstem = < 25,000 km^2^, 5 Large = < 100,000 km^2^, 6 Great = ≤100,000 + km^2^, and 7 Mega = 1,000,000 + km^2^).

Locations of 152,508 dams were compiled from multiple sources [30–34]. For dams on rivers (Size 2 or larger, hereafter “*river dams*,” 7,399 locations), we manually reviewed their attributes and “snapped” their spatial location to the correct streamline for the FCN analysis. Dams with fish passage structures, navigation locks, or submerged at high flows were identified using multiple criteria and tagged as partially passable [35]. For dams on headwaters (Size 1, hereafter “*headwater dams*,” 145,109 locations), we did not review their spatial accuracy or use them to create FCNs. Instead, we developed a headwater fragmentation metric for the HUC-12 scale (S1 File).

### Resilience model

**Fig 1:**
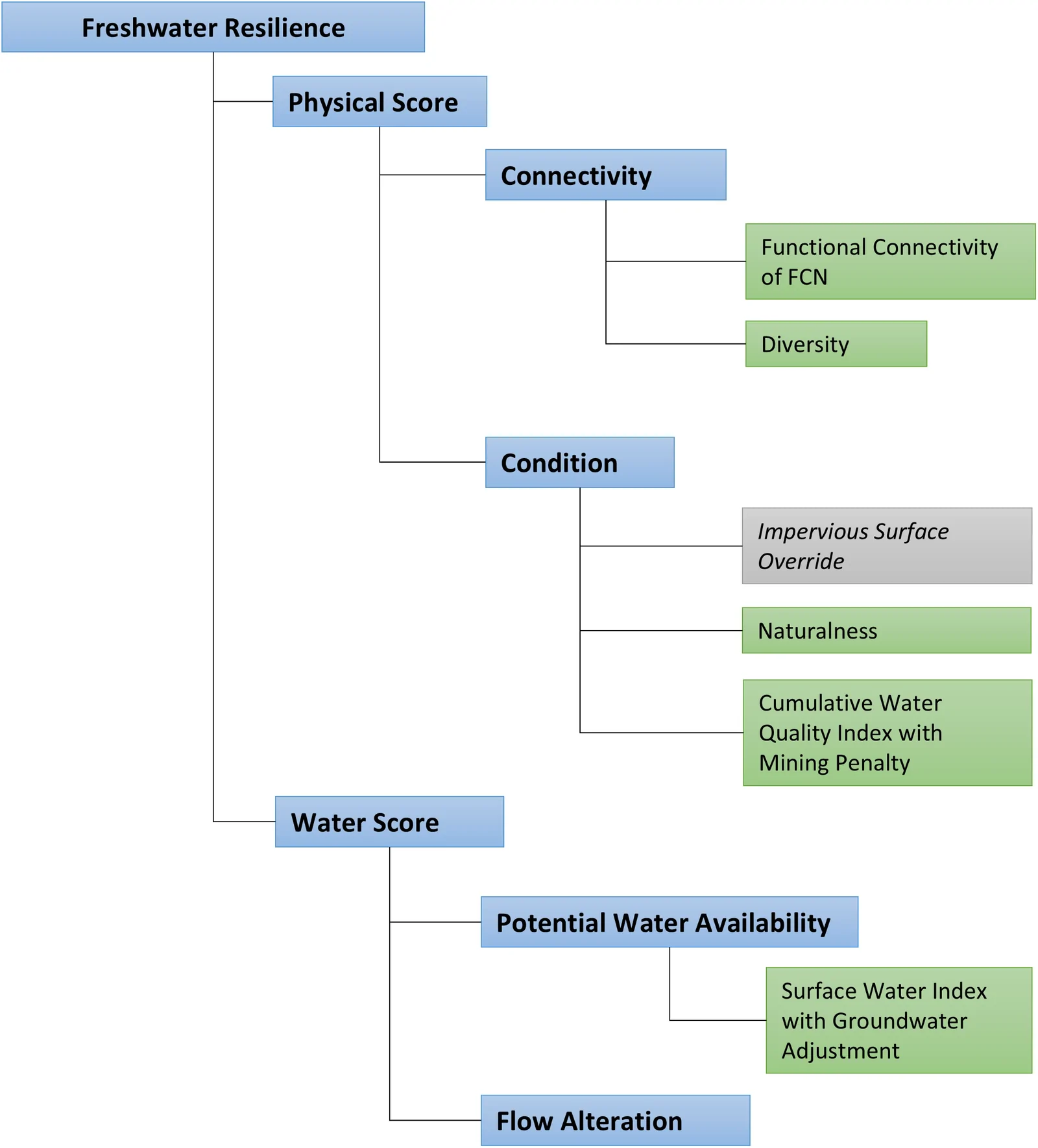
General model for the freshwater resilience score.

Our model for estimating freshwater resilience was derived from Higgins et al.’s [36] Key Ecological Attributes (KEAs) as fundamental characteristics of freshwater ecosystems essential for the long-term persistence of biodiversity (Fig 1). Our resilience score integrates the components of connectivity, habitat, water quality, and hydrologic regime. Biotic composition was not included as it is necessarily in flux with ongoing climate change and the needed data do not exist.

Each component was quantified and scored using specific metrics. For example, the Functional Connectivity component was quantified as the length of the FCN adjusted for passible dams and connections to the ocean or Great Lakes. All intermediate scores were calculated at the HUC-12 scale except for Functional Connectivity and Temperature Diversity, which were calculated at the FCN scale. Final scores were assigned to units created by the intersection of FCNs and HUC-12s.

The components were combined into a resilience score by applying weights based on the climatic zones. The relative weighting of the components was determined by testing the models in different geographic regions and reviewing the results with steering committee members. This approach enabled us to calibrate the model to local conditions while still using the same set of components throughout CONUS, thus ensuring the results would be useful for local planning as well as CONUS-wide. Primary weighting and stratification are described below with full details in the S1 File. Finally, before combining components and/or metrics, we converted all scores to standard normal units (z-scores), transforming them as needed to approximate a normal distribution and capping them at −3 SD to 3 SD to avoid outlier influence. Due to extensive hydrologic alteration, several core datasets were not available for the South Florida ecoregion, thus we excluded this area from resilience scoring.

### Physical score


$$\begin{array}{c} \text{Physical Score }=\text{ Connectivity }+\text{ Condition} \end{array}$$

The physical score was designed to describe the size and diversity of each FCN and the condition of its internal waters and wetlands. For river corridors, resilience derives from connectivity, spatial heterogeneity, and ecological integrity [37]. Specifically, larger, more complex networks impart resilience by providing greater access to life-stage habitat needs, refugia, and unimpaired riverine processes.

### Connectivity

#### Functional connectivity.

We intersected the river dams with the dendritic river network and delineated the FCNs based on each connected river network bound on all sides by dams, headwater sources, or its terminus (e.g., confluence with an ocean or Great Lake). For each FCN, we measured its length and the size of its immediate drainage area. Using drainage area, we grouped the FCNs into eleven size classes applying Natural Breaks [38] to account for their skewed distribution towards small areas. We adjusted the size class of some FCNs upward to account for passable dams and/or connectivity to the ocean or Great Lakes, and downward to account for the presence of large reservoirs and/or headwater fragmentation (S1 File).

#### Diversity.

We extracted the estimated mean summer stream temperature for each stream reach using Hill et al. [39] supplemented by Lyons et al. [40] and classified the continuous temperature predictions into five classes following McManamay et al. [41]. For each FCN, we tabulated the number and percentage of temperature classes and created a cold-weighted temperature diversity index based on classes making up over 5% of the FCN (S1 File). We converted the results to z-scores and added 0.5 SD if the Hill et al. [42] model indicated the lowest temp class was maintained in predicted 2.2°C warmer waters.

To estimate the diversity of physical habitats, we created fine scale macrohabitats [43] based on abiotic variables that structure aquatic communities: stream size class, gradient, local temperature, and tidal influence. We used Poisson regression to establish a relationship between the size of the HUC-12 and the count of macrohabitats and then calculated the standardized residuals to identify watersheds that had higher or lower diversity than expected for their size. We adjusted the scores upward to account for perennial streams and downward for headwater fragmentation (S1 File).

Finally, we calculated an integrated diversity score for each HUC-12 by averaging the temperature score with the macrohabitat score.

#### Connectivity score.

We calculated a final connectivity score for each HUC-12 by integrating the functional connectivity score measured at the FCN scale with the diversity score measured at the HUC-12 scale. To account for the greater importance of FCN size over the more nuanced measure of diversity, we gave connectivity twice the weight.


$$\begin{array}{c} Connectivity\;Score\;=\;(\text{2}*Functional\;Connectivity\;+\;\text{1}*Diversity)/\;\text{3} \end{array}$$

### Condition

#### Land cover naturalness.

We assessed the land cover naturalness of three essential river features – the floodplain, riparian area, and watershed – and then combined them into an index of naturalness giving more weight to undeveloped floodplain and riparian zones to reflect the importance of their direct interaction with streams and rivers. For each feature, we developed a map of its extent and then overlaid land cover data augmented by mining locations, to quantify the types and amounts of natural, agricultural, and developed/mined land within each extent [44]. We combined the land cover classes into a class-weighted index ranging from 0 for completely natural cover to 100 for completely developed. Transforming the three individual results to z-scores, we then combined them into a floodplain/riparian weighted Land Cover Naturalness score (LCN) stratifying and making minor adjustments by climatic regions (S1 File).

#### Water quality.

We used the USGS SPARROW model to estimate water quality for all regions except southeast Florida for which data was unavailable [45–49]. For each stream catchment, we summarized cumulative yield values for total nitrogen (TN), total phosphorus (TP), and suspended sediment (SS), and converted the values to z-scores by arid and humid regions. Because the TN and TP z-scores were strongly correlated, we took an average of the TN and TP z-scores and then averaged that score with the SS z-score to calculate a cumulative water quality index for each catchment. We multiplied the cumulative water quality z-score by the proportion of the catchment in each HUC-12 and summed the values. In HUC-12s with a high percentage of mining area, we further lowered the score in proportion to the mining area (S1 File) to account for the negative impacts mining activities can have on water quality.

#### Impervious surface override.

We overlaid the NLCD percent imperviousness data [44] on the HUC-12s and assigned a condition score of −3.0 SD to those with over 20% impervious surfaces [50] to account for the detrimental impacts of high imperviousness on freshwater systems. This affected less than 2% of the HUC-12s.

#### Condition score.

We created a condition score equal to the sum of the components stratified and weighted by climatic region, giving slightly more weight to water quality in the humid region where the range of degradation was greater. We capped the condition z-score at 1 SD in the xeric region to limit the influence of natural conditions in watersheds with little or no permanent water (S1 File).

Condition:


$$\begin{array}{c} Humid:\;(\text{5}*Naturalness\;+\;\text{3}*Water\;Quality)/\text{8}\;-\;Impervious\;Surface\;Override \end{array}$$

$$\begin{array}{c} Arid/Xeric:\;(\text{5}*Naturalness\;+\;\text{2}*Water\;Quality)/\text{7}\;w\;Cap\;-\;Impervious\;Surface\;Override \end{array}$$

#### Final physical score.

The final physical score was an equally weighted combination of the connectivity and condition scores


$$\begin{array}{c} Physical\;Score\;=\;(\text{1}*Connectivity\;+\;\text{1}*Condition)\;/\;\text{2} \end{array}$$

### Water score


$$\begin{array}{c} Water\;Score\;=\;Potential\;Water\;Availability\;+\;Hydrologic\;Alteration \end{array}$$

The water score was designed to estimate the availability of ample unaltered water within each river network. As the natural levels of precipitation, surface water, and groundwater differ markedly across the U.S., we stratified the results by climate region to obtain accurate and locally relevant results. The score is a function of potential water availability and hydrologic alteration, which we describe in detail below. We also recognize that water availability is in flux with climatic change. However, areas with enduring sources of available water, greater ability to store potential rainwater, and less hydrologic alteration are likely to be more resilient to climate change relative to their climatic region [51–53]. Thus, the water score was designed to estimate the relative availability of water and unaltered hydrology in each HUC-12.

### Potential water availability

#### Surface water index.

We created a surface water index based on hydrologically relevant topographic features that collect and store water. Our input data included perennial lakes, streams, rivers, springs, pluvial depressions, wetlands, floodplains, and moisture accumulating flats. These were compiled from multiple sources and adjusted to account for tile drainage in agricultural areas [54]. In areas at or above the western upper montane zone, we created a 30-m snowpack persistence map based on elevation and topographic influence to identify areas expected to store and slowly release snowmelt. Each index input was mapped and assigned a weight from 0 to 500 based on their relative importance to sustaining water in river networks (S1 File). Scores were summed for each HUC-12 and transformed to z-scores relative to CONUS.

#### Groundwater adjustments.

We adjusted the surface water score in HUC-12s where groundwater indicators suggested abundant or depleted groundwater resources (S1 File).

Springs (positive): We calculated the density of springs and seeps in each HUC-12 and converted the results to z-scores relative to all HUC-12s that contained a spring. We extracted all HUC-12s above the mean density and applied an increase to their water availability score by rescaling their z- scores to be between 0–1 and applying this score as an override if the score was higher than the surface water score.

Perennial Streams and Rivers in Arid Ecoregions (positive): In arid and xeric ecoregions we accounted for the importance of perennial streams and rivers using an override. We calculated the percentage of perennial systems in each HUC-12 and scored them from 0.1 SD (10%) to 1.0 SD (100%). HUC-12s were assigned the perennial score if it was greater than the surface water z-score. If a HUC-12 contained a perennial river greater than 1,000 km^2^ drainage area, the score was also increased to a minimum of 1 SD.

Groundwater Depletion (negative): We evaluated groundwater depletion in the arid and xeric regions using NASA’s GRACE Shallow Groundwater Drought Indicator [55,56], which measures the current alteration of groundwater volume compared to historic levels. Using the recharge months of February and March, we compiled the data for an 18-year period (2003–2021) relative to a 64-year average (1948–2012). We categorized the results into percentile classes representing the most common depletion level over the 18-year period and applied a groundwater penalty to the severely altered class (0–20% historic levels).

We applied the depletion penalty based on how dependent the area was on groundwater as its main source of water. To assess this, we developed hydrologic landscape regions (HLRs) based on dominant soil, bedrock, and aquifer characteristics following Wolock et al. [57] using revised soil, geology, and landform data [58–60]. Using the HLRs as a spatial framework, we classified HUC-12s as high, moderate, or low for natural groundwater potential. HUC-12s in the high class received a penalty of −0.25 to −0.50 SD, proportional to the percentage of the HUC-12 covered by the GRACE severely altered groundwater depletion class. Similarly, HUC-12s in the moderate class received a penalty of −0.05 to −0.25 SD, and those in the low class received no penalty.

#### Potential water availability score.

The final potential water availability score was calculated from the surface water index with the groundwater modification applied as follows.

*Potential Water Availability:*


$$\begin{array}{c} \text{Humid}\;=\;\text{Surface Water Index with Spring Density Override where applicable}. \end{array}$$

$$\begin{array}{c} \frac{Arid}{Xeric}=\begin{array}{c} Surface\;Water\;Index\;with\;Spring\;Density\;or\;Perennial\;Rivers\;Override\;and\\ \;HLR\;Weighted\;Groundwater\;Depletion\;penalty\;applied\;where\;applicable \end{array}\; \end{array}$$

#### Hydrologic alteration.

We accounted for flow alteration using a Hydrologic Alteration Index (HAI) [61], which used random forest models to assign a degree of flow alteration to each stream reach based on a comparison of current stream flows to reference conditions at a set of gages. We summarized the HAI for all river reaches in the HUC-12 using a length-weighted score that accounted for whether the river was intermittent or perennial. HUC-12s were scored across CONUS to derive a final hydrologic alteration score where +3.0 SD equaled no alteration. In the arid region, we capped the flow alteration score at 1.0 SD in HUC-12s with over 75% intermittent streams and rivers, because these systems did not have enough surface water to experience flow alteration (S1 File).

### Integrated water score

For each HUC-12, we integrated hydrologic alteration (HA) and potential water availability (PWA) into a water score by taking a weighted average of the two scores, with the weights differing substantially between climatic region and by the percent of intermittent streams in the HUC.

*Water Score:*


$$\begin{array}{c} Humid\;=\;(\text{3}*HA\;+\;\text{1}*PWA)/\text{4} \end{array}$$

$$\begin{array}{c} Arid/Xeric\;=\;(\text{1}*HA\;+\;\text{1}*\;PWA)/\text{2} \end{array}$$

$$\begin{array}{c} Arid/Xeric\;and\;Intermittent\;>\text{7}\text{5}\%\;=\;(\text{1}*HA\;+\;\text{3}*PWA)/\text{4} \end{array}$$

### Resilience score

We calculated a resilience score for each HUC-12 by calculating a weighted average of the physical score and the water score, with the relative weight of each component dependent on the climatic region.

*Resilience Score:*


$$\begin{array}{c} Humid\;=\;\text{2}*Physical\;Score\;+\;\text{1}*Water\;Score \end{array}$$

$$\begin{array}{c} Arid\;=\;\text{1}*Physical\;Score\;+\;\text{1}*Water\;Score \end{array}$$

$$\begin{array}{c} Xeric\;=\;\text{1}*Physical\;Score\;+\;\text{2}*Water\;Score \end{array}$$

$$\begin{array}{c} Xeric\;and\;Intermittent\;>\text{7}\text{5}\%\;=\;\text{1}*Physical\;Score\;+\;\text{4}*Water\;Score \end{array}$$

#### Ecoregional override.

To ensure representation of resilient freshwater networks in all ecoregions, we recalculated the HUC-12 z-scores relative to each freshwater and terrestrial ecoregion, then selected the HUC-12s that were above average (>0.5 SD) relative to the ecoregional mean. If a HUC-12 was average relative to CONUS (−0.5 and 0.5 SD) but above average relative to its ecoregion, we adjusted its final score upward to be between 0.5 and 1 SD (S1 File). This raised the score of 3% of the HUC-12s.

### Recognized biodiversity value

We compiled 91 state, ecoregional, regional, and species-specific freshwater studies that identified sites for conserving aquatic biodiversity due to their exemplary habitat or unique biodiversity value. National sources included 57 TNC freshwater ecoregional assessments created between 1998–2013, with sites identified through intensive, expert-driven planning efforts based on Natural Heritage Program inventory data. State-based assessments included State Wildlife Action Plans for 31 states, Crucial Habitat Assessment Tool designations for Oklahoma and Texas (S1 File), and a freshwater biodiversity blueprint completed for California by TNC [62]. Species-specific assessments included anadromous species critical habitat [63], critical habitat for aquatic or aquatic-dependent inland species [64], secure strongholds for brook trout in the Eastern U.S. [65], bull trout and cutthroat trout strongholds in the Western U.S. [66], areas with rare aquatic species [67], and others (S1 File).

All delineations of habitat were attributed to reach catchments from their original source data, which included flowlines, hexagons, watersheds, and free-form polygons. We merged the data into one dataset, preserving a set of consistent attributes.

### Freshwater opportunity map

To identify resilient and biodiverse freshwater conservation opportunities in CONUS, we integrated the freshwater recognized biodiversity map with the freshwater resilience map and categorized the various combinations. We defined “*resilient opportunity systems*” as those areas with both recognized biodiversity value and above-average resilience (>0.5 SD) or with very high resilience only (>1 SD). We defined “*restorable opportunity systems*” as those with recognized biodiversity value, average resilience (−0.5 to 0.5 SD) and only one factor below average (<−0.5). We defined “*vulnerable opportunity systems”* as those with recognized biodiversity value and lower resilience. To identify resilient wetlands not associated with riverine features, we compiled high scoring (>0.5 SD) wetlands, floodplains, riparian areas, and groundwater-dependent ecosystems assessed and scored through an independent terrestrial resilience assessment [19]. Using the same terrestrial dataset, we identified resilient upland areas that were located within the headwaters of resilient and restorable small rivers (100−1,000 km^2^ upstream drainage areas).

### A Freshwater Resilient and Connected Network (FRCN)

We identified a network of freshwater sites that could contribute meaningfully to the CBD Target 3 goal of conserving, connecting, and restoring at least 30 percent of our inland waters by 2030. The network is a subset of the freshwater opportunity map, however, we did not set a goal for spatial extent but instead determined how much area was required to meet our criteria for representation, resilience, connectivity, and biodiversity (Table 1). We began by restricting the criteria to “resilient opportunity systems” but it became apparent that to represent all CONUS freshwater species and ecosystems, we would need to include “restorable opportunity systems” as defined above. Thus, we applied the criteria to both the resilient and restorable HUC-12s, categorizing the latter by their below-average factor. This indicated the dominant intervention needed to restore resilience: reconnect (connectivity), restore condition (condition), restore flow (hydrologic alteration), and verify water (water availability). Where needed, we applied additional criteria to ensure the resilience of the HUC-12.

**Table 1:** Criteria for identifying the Freshwater Resilient and Connected Network (FRCN). Scores are in standard deviations relative to the mean of the climatic region.

FRCN Criteria	Final Scores		Resilience Component Scores				Additional Criteria
	Re-silience	Bio-diversity	Connectivity	Condition	Water Alteration	Water Avail-ability	
**Highest Resilience**							
	**>1.0 SD**	-	-	-	-	-	
**Resilient and Biodiverse**							
	**>0.5 SD**	Y	-	-	-	-	
**Restore Condition**							
Humid	Avg.	Y	>=Avg.	**Below Avg.**	>=Avg.	-	
Arid	Avg.	Y	>=Avg.	**Below Avg.**	>=Avg.	**>= Avg.**	
Xeric	Avg.	Y	>=Avg.	**Below Avg.**	>=Avg.	**>= 0.5 SD**	
**Restore Flow**							Sites could not be just headwaters but had to contain some portion of size 2 river
	Avg.	Y	>=Avg.	>=Avg.	Below Avg*	-	
**Restore Flow and Condition**							
	Avg.	Y	>=Avg.	**Below Avg.**	Below Avg*	-	
**Reconnect**							FCN must be bound by dams and current network 60% or less of its natural size.
Humid FCN	**>=Avg.**	Y	**Below Avg.**	>=Avg.	>=Avg.	-	
Humid HUC	Avg.	Y	**Below Avg.**	>=Avg.	>=Avg.	-	
Arid FCN	**>=Avg.**	Y	**Below Avg.**	>=Avg.	>=Avg.	**>=Avg.**	
Arid HUC	Avg.	Y	**Below Avg.**	>=Avg.	>=Avg.	**>=Avg.**	
**Verify Water (field check to confirm surface and groundwater)**							Sites had to be the best in their T/FW Ecoregion (>0.5 SD)
	Avg.	Y	>=Avg.	>=Avg.	>=Avg.	**Avg.**	

Statistical Definitions: Avg. -0.5 to 0.5 SD, Below Avg. -2.0 to -0.5 SD, Below Avg* <= -0.5 SD

### Representation

To assess how well the FRCN represented all types of freshwater ecosystems and species, we overlaid individual datasets including ecoregions, stream size classes, lakes and ponds, wetlands, floodplain and riparian areas, and geophysical settings (one of 30 bedrock or surficial geologic classes found to correlate with terrestrial species [19]). Additionally, we measured how well the FRCN captured unfragmented river stretches, and we overlaid a global map of groundwater-dependent ecosystems [68]. To further evaluate biodiversity representation, we overlaid each component of the recognized biodiversity layer. Additionally, we calculated the total number of globally rare native fish, mussels, and crayfish per HUC-8 watershed [68–70] and compared that to the percentage of the FRCN that covered each HUC-8. Similarly, we calculated the number of total fish species and endemics in each ecoregion and compared that to the percentage of FRCN in the ecoregion.

### Conservation status

We defined a “conserved” ecosystem as 1) above a threshold for resilience ensuring the system’s function, condition, and adaptive capacity are intact, and 2) protected from further degradation based on sufficient legal mechanisms or community management agreements that prevent degradation [36]. To complement our resilience metrics, we developed a spatially explicit CONUS-wide measure of protection to determine where mechanisms exist to prevent degradation at both local and watershed scales. Local designations restrict activities in specific reaches of rivers and their corridors, while regulating functions are sustained by the extent and spatial distribution of conservation lands within the contributing area of a river [71,72].

To evaluate local protection, we used the Protected River Index (PRI) pioneered by Comte et al. [73]. The PRI includes five categories of local protection (river conservation, riparian and floodplain conservation, endangered species critical habitat, terrestrial protected areas, and multiple-use land) and weights each protection mechanism according to its potential conservation effectiveness in protecting each KEA [36]. We calculated the PRI over the length of full reaches to match the rest of our analysis [73].

To evaluate regulating function, we estimated the protective benefits of upstream conservation lands for the protection of water quality and flow regime using a recently compiled dataset of U.S. conservation lands [74]. We assigned relative protection benefits to the different categories of conservation ownership, and accumulated the amount, type and value of all conservation lands upstream of each reach. We transformed the results into a regulating function score between 0 and 100 (S1 File).

Lastly, we transformed the local protection and regulating function indices to the same scale and combined them into a protection class score:


$$\begin{array}{c} Protection\;Class\;=\;(Local\;Protection\;PRI\;+\;Regulating\;Function)/\text{2} \end{array}$$

To evaluate the conservation status of the FRCN, we calculated the protection class of each reach and graphed the results against the resilience scores to highlight protection and restoration needs.

## Results

### Freshwater resilience

**Fig 2:**
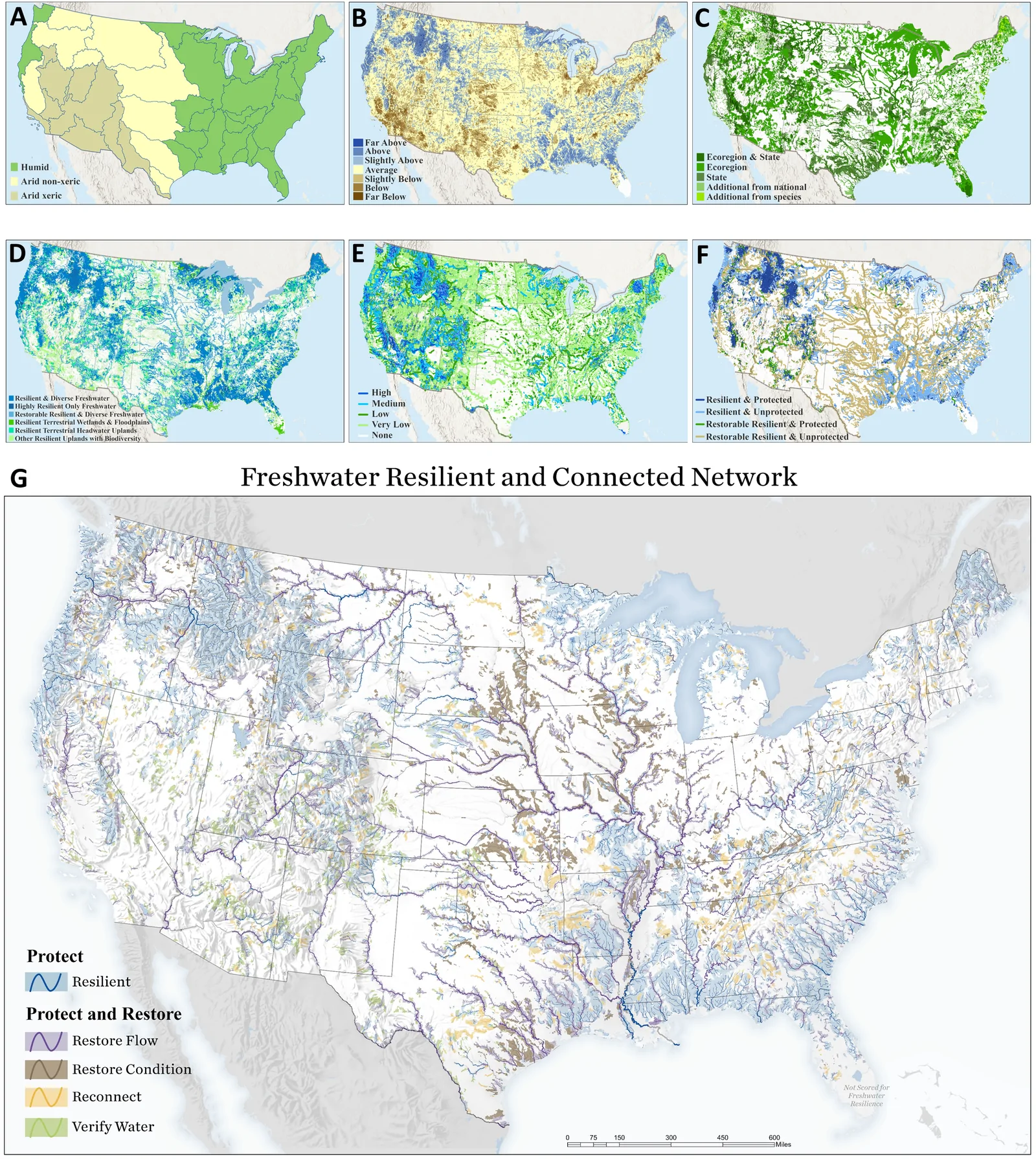
Components and results of the analysis. **A.** Climatic Regions, **B.** Freshwater Resilience, **C.** Freshwater Recognized Biodiversity Value. **D.** Freshwater Opportunity Map. **E.** Freshwater Conservation Status **F.** FRCN Conservation Status. **G.** Freshwater Resilient and Connected Network (FRCN). These maps were created with ArcGIS Pro 3.5.2 (ESRI, Redlands, CA). Hillshade used in Figures A-F from World Hillshade (Sources: Esri, Airbus DS, USGS, NGA, NASA, CGIAR, N Robinson, NCEAS, NLS, OS, NMA, Geodatastyrelsen, Rijkswaterstaat, GSA, Geoland, FEMA, Intermap, and the GIS User Community) [75]. Hillshade used in Figure G from Herwig G. Schutzler, 1965, Shaded Relief Archive, https://shadedreliefarchive.com/usa-schutzler.html.

Of the 5.2 million kilometers of rivers and streams in CONUS, 26% scored above-average for resilience (>0.5 SD) with 7% far-above-average (>1 SD). Resilient river networks were concentrated in the Pacific Coast, Northern Rocky Mountains, Superior Mixed Forest, Northern Maine, the Southeast Coastal Plain, and the Gulf Coastal Plain ecoregions (Fig 2B). River networks that scored average (−0.5 to 0.5 SD) were common (59%) and widely distributed. Vulnerable or below-average river networks (<−0.5 SD) made up 15% of the stream kilometers and were concentrated in the Southwest Deserts, Shortgrass Prairie, North Central Tillplain, and Lower New England. Causes of vulnerability varied from water availability in the deserts to poor condition in the Tillplain to fragmentation in Lower New England. Fragmentation was pervasive with 7,399 dams partitioning CONUS rivers into 9,180 Functionally Connected Networks (FCNs) ranging in size from less than 1 km^2^ to over 277,000 km^2^. Another 145,109 headwater dams occurred on small streams.

### Recognized biodiversity value

Just over 47% (2.4 million km) of CONUS rivers and streams have been recognized for their biodiversity value (Fig 2C). Of these, 25% came from a TNC ecoregional assessment, 10% from a state-based survey, and 8% from both. An additional 2% came from national data on rare species and 1% from a species-specific analysis. Biodiversity sites were distributed across all freshwater ecoregions, averaging 52% of an ecoregion’s stream miles. Among all biodiversity sites, 34% scored resilient, 56% scored average, and 10% scored below-average.

### Freshwater opportunity map

The intersection of freshwater resilience with recognized biodiversity value, and the addition of resilient wetlands and headwaters from a parallel terrestrial analysis [19] created a broad map of freshwater opportunities covering 53% of CONUS (Fig 2D, Table 2). Networks with recognized biodiversity value and above-average resilience covered 17%. Those with biodiversity value and average resilience accounted for an additional 16%. Resilient wetlands, floodplains, and waterbodies accounted for another 6%, headwaters for 7%, and resilient land with vulnerable freshwater diversity for 5%. Representation of freshwater features in the opportunity map was extremely high, with wetlands (73%), groundwater-dependent wetlands (83%) and springs (84%) captured in large proportions. Representation of riparian and floodplain wetlands (52%) and groundwater-dependent uplands (43%) was slightly lower, perhaps reflecting their vulnerability in the arid zone.

**Table 2:** Results of applying the freshwater opportunity, Freshwater Resilient and Connected Network, and conservation status criteria as a percentage of CONUS stream kilometers. We defined “restorable” as average resilience with recognized freshwater biodiversity. Non-riverine wetlands were assessed in an independent terrestrial resilience analysis [19].

FRESHWATER OPPORTUNITIES	
**Freshwater Resilient and Connected Network 35%**	
**Resilient and Diverse Freshwater Ecosystems**	
16%	Resilient freshwater systems recognized for biodiversity.
1%	Resilient freshwater waterbody recognized for biodiversity.
**Highly Resilient Only**	
2%	Highly resilient freshwater systems NOT recognized for biodiversity
**Restorable Resilient and Diverse Freshwater Ecosystems**	
16%	Restorable freshwater systems recognized for their biodiversity (one factor below average)
(4%)	Restore Condition
(5%)	Restore Flow
(3%)	Restore Flow and Condition
(3%)	Reconnect
(2%)	Verify Water (potential subtractions if unable to confirm local water source)
**35%**	
**Other Freshwater Resilient Opportunities 18%**	
**Resilient Terrestrial Wetlands, Floodplains and Waterbodies**	
4%	Resilient wetlands and floodplains recognized for their terrestrial biodiversity
0%	Resilient tidal marsh with adjacent migration space
1%	Resilient wetlands
1%	Non-resilient wetland and floodplain in resilient freshwater HUC
**Headwater Uplands**	
2%	Resilient terrestrial and resilient freshwater
5%	Resilient terrestrial and average or less freshwater
**Resilient Uplands with Biodiversity**	
1%	Resilient terrestrial and freshwater uplands recognized for terrestrial biodiversity
4%	Resilient terrestrial upland with freshwater biodiversity.
**Total 53%**	

### Freshwater resilient and connected network

Application of 30x30 inspired criteria to the opportunity map identified a Freshwater Resilient and Connected Network (FRCN) covering 35% of CONUS by area (Fig 2E, Table 2). In total, 19% of assessed reaches scored as resilient and/or biodiverse, and another 16% scored as restorable and biodiverse. Of the latter, strategy needs were almost equally distributed: restore flow (5%), restore condition (4%), restore flow and condition (3%), reconnect (3%), and verify water (2%). Collectively, the network contained streams and rivers in a similar size-class proportion as for CONUS with a 1% bias towards larger rivers.

The FRCN captured 51% of all CONUS wetlands, 58% of groundwater-dependent wetlands, 47% of all lakes and ponds, and 44% of all springs. It overlapped strongly with free-flowing rivers that had a terminus in the ocean (480 FCNs, 77% overlap), the Great Lakes (199 FCNs, 82% overlap), or was a long tributary connected to a mainstem river (22 FCNs, 90% overlap). The latter included the Salmon, Grande Ronde, Powder, Little Missouri, Moreau, White, Little Snake, Quinn, Amargosa, San Pedro, Santa Crux, Cimarron, Salt Fork Brazos, Beals Creek, Meramec, Grand, Wabash, St. John, and other rivers. Most ocean-reaching FCNs were small, but 23% (109) were large rivers with long stretches of barrier-free mainstem. These included the Mississippi, Sacramento, Pascagoula, Neuse, Klamath, Rouge, Columbia, Suwannee, Altamaha, Pee Dee, Delaware, and other rivers.

The FRCN captured at least 30% of all geological settings except for calcareous loam (23%), deep loess (17%), caliche (16%), and gypsum (7%). It also captured at least 28% of all elevation zones (definitions in [19]).

Of the 38 freshwater ecoregions, 58% had over 30% of their stream kilometers represented in the FRCN and this increased to 97% of ecoregions if counting only perennial streams. Ecoregions with more fish species had a slightly higher proportion in the FRCN (P = < 0.001, R2 = 0.07). In the three ecoregions with the highest species richness and at least 20 endemics, the FRCN made up 40−60% of the stream kilometers: Lower Mississippi (256 species), Tennessee (242 species), and Mobile Bay (237 species). In contrast, for the three ecoregions with the lowest species richness and less than 10 endemics, the FRCN made up 15−25% of stream kilometers: Bonneville (21 species), Death Valley (15 species), and Sonora (12 species). Representation of recognized biodiversity components ranged from 67% to 91% and was particularly high for anadromous (>72%) and coldwater (>86%) fish. Of the HUC-8 watersheds containing rare species, 99% overlapped with the FRCN. Those with over ten rare species were well within the FRCN while those with fewer were in or partially within the FRCN.

### Conservation status

The conservation status of the FRCN varied spatially. By length, the FRCN comprises 35% of stream miles in CONUS. Length and catchment area are highly correlated, but length is more specific for measuring stream protection. Of all stream miles, 6% had above-average resilience and medium-to-high protection, while 14% had average resilience and low-to-no protection. The former class was concentrated in mountainous regions and the latter in the agricultural centers of the Midwest and Great Plains (Fig 2F). Another 13% had above-average resilience but low protection. These networks were spread among the Southeast, Pacific Coast, Great Lakes, and northern New England. The remaining 2% with medium-to-high protection, but only average resilience, were in the arid Southwest. The distribution of protection across the FRCN was similar in proportion to that for all streams in CONUS, suggesting that (unlike for land) the resilience scores were independent of protection status.

## Discussion

To inform actions to reverse freshwater biodiversity loss exacerbated by a changing climate, we completed a four-year study to assess the resilience of streams and rivers in CONUS. We combined the resulting resilience scores with recognized freshwater biodiversity areas and resilient non-riverine wetlands and headwaters to create a comprehensive freshwater opportunity map. Selecting from the latter, we identified a conservation network that met criteria for ecological representation, resilience, connectivity, and biodiversity. The resulting Freshwater Resilient and Connected Network (FRCN) covered 35% of CONUS and, if restored and conserved, could theoretically sustain a wide spectrum of aquatic biodiversity into the future. The conservation challenges are large. Only 6% had both high protection and resilience, and at the opposite extreme, 14% needed both protection and restoration.

The FRCN provides a spatially explicit picture of what we seek to conserve, and the dominant strategies needed to restore or protect it. It can serve as a 30x30 target for conservation, fulfilling in design and characteristics CBD Target 3’s standards for inland waters. However, in addition to protection, almost half the network will require restoration to enhance resilience and sustain diversity. We hope it stimulates the further development of spatial action maps [76] depicting where to work, who to work with, and what strategies to employ. Although we highlight key conservation actions that will improve resilience, we recommend users make a local assessment of ecological integrity and threats [36] and review the environmental justice implications of potential actions, prior to investing in specific strategies.

The information presented here is intended to be used in conjunction with local information and on-the-ground surveys. The extent of the analysis created limitations in source data selection because consistency was needed across the study area to avoid bias from local data discrepancies. The resolution of the underlying datasets also varied from 10-m raster floodplains to 12.5-km GRACE shallow groundwater data. Despite this variation, we sought to use the appropriate summary scales for each metric, to combine variables in a logical multi-scale framework, and to stratify the results by relevant climate zones or ecoregions to increase local relevance of the results.

Our application of weighting factors was tested and informed by multiple rounds of expert review in different geographies as well as by our own visual inspection of local and national results. A pattern we noticed frequently in our sensitivity testing was that the results were less sensitive to the weights than we expected. Often, changes in weights, even by two or three integers, resulted in category changes in less than 1% of the HUC-12/FCN units. This led us to rely on expert inspection of well-studied local stream networks and gave us confidence in the stability of the results. In contrast, stratification of the results by climatic zone and ecoregions had a dramatic impact on the distribution of values. Further refinements to those boundaries, or exploration of their influence, could provide improvements to the results.

To make the results available to a broad range of audiences, and support their interpretation and application by practitioners, we developed the Resilient River Explorer web application (https://www.maps.tnc.org/resilientrivers). The tool is organized into thematic sections, each corresponding to different ways to use the results to inform conservation actions. Additionally, users can provide feedback and report dam errors through the tool and find resources such as data downloads and map service links.

We acknowledge that the needs of people are not represented in this analysis as we focused on other species that also need abundant clean freshwater. We recognize that maps are not neutral about human equity, both in what is interpreted to be important for conservation action, the sourcing of the data used, and who participated in the analysis. Additional data representing human dimensions could be added in the future. As a guide to how this might work, TNC’s Appalachian freshwater team, working with representatives from federal agencies, used the results in conjunction with a suite of socioeconomic data to identify watersheds in the Appalachians where restoration of freshwater connectivity could benefit both nature and people. We have recently expanded this application to CONUS (https://www.maps.tnc.org/raw).

We hypothesized that by assessing the enabling conditions for freshwater systems to adapt and thrive, we could reduce some of the uncertainty of climate change impacts. Extrapolating from evidence, we suggest that networks with more habitat options, natural floodplains, access to groundwater, and other resilience characteristics will be more adaptive relative to similar networks with fewer of these characteristics [77]. However, our metrics are necessarily relative because there is not a specific threshold that guarantees a river to be resilient, and even rivers with high scores may have restoration needs. Thus, the intention of the analysis and associated tool is to provide conservationists in the U.S. a means to evaluate the relative resilience of freshwater river networks so action can be taken to maintain and improve our rivers and sustain the nation’s rich freshwater biodiversity.

Among TNC and partners, the analysis has been used to identify dams for mitigation or removal, prioritize land protection in key headwaters, and improve conditions in restorable river systems. For example, a joint study was implemented with the Sustainable Rivers Program (SRP), a collaboration between TNC and the U.S. Army Corps of Engineers (USACE), to examine the climate resilience of rivers influenced by USACE-managed reservoirs, locks, and dams and the potential for flow management to increase resilience. A review of rivers where TNC is currently engaged indicates that most TNC freshwater conservation activities are focused on FRCN rivers that need both restoration and protection, providing a clear picture of the challenges in sustaining diverse and adaptive U.S. rivers.

## Supporting information

S1 File Supplemental for PLOS FW Resilience 20260303. (DOCX)
