# Supplementary material for: A freshwater resilient and connected network to sustain biodiversity under a changing climate
Source: PLoS One. 2026 Sep 16;21(9):e0351782. doi: 10.1371/journal.pone.0351782 (PMC13581213; doi:10.1371/journal.pone.0351782)
Supplement: Supplementary file 1 [file pone.0351782.s001.docx]

**Supporting Information for**

A freshwater resilient and connected network to sustain biodiversity under a changing climate

Mark G. Anderson [mandersonmga@gmail.com](mailto:mandersonmga@gmail.com), Arlene P. Olivero [arlene_olivero@tnc.org](mailto:arlene_olivero@tnc.org), Analie R. Barnett [abarnett@tnc.org](mailto:abarnett@tnc.org), Mary L. Khoury [mkhoury@tnc.org](mailto:mkhoury@tnc.org), and Erik H. Martin [emartin@tnc.org](mailto:emartin@tnc.org)

Corresponding Author: Mark G. Anderson

Email: [mandersonmga@gmail.com](mailto:mandersonmga@gmail.com)

**This PDF file includes:**

Supplementary Text

Figures S1 to S75

Tables S1 to S24

Supplementary References

Table of Contents

[Large Format Maps from Main Paper	1](#_Toc202356168)

[Model Diagrams	9](#_Toc202356169)

[Mapping Freshwater Resilience	15](#_Toc202356170)

[Physical Score	15](#_Toc202356171)

[Connectivity Score	15](#_Toc202356172)

[Functional Connectivity	15](#_Toc202356173)

[Delineating FCNs	16](#_Toc202356174)

[FCN Size Class	17](#_Toc202356175)

[Accounting for Partially Passable Dams	19](#_Toc202356176)

[Connectivity to Large Water Bodies	20](#_Toc202356177)

[Reservoir Penalty	20](#_Toc202356178)

[Headwater Fragmentation	22](#_Toc202356179)

[HUC-12 Headwater Fragmentation	22](#_Toc202356180)

[FCN Headwater Fragmentation	24](#_Toc202356181)

[Scoring of HUC-12s not within an FCN	26](#_Toc202356182)

[Final Functional Connectivity Score	26](#_Toc202356183)

[Diversity	28](#_Toc202356184)

[Temperature Diversity	29](#_Toc202356185)

[Temperature Classes	29](#_Toc202356186)

[Weighting for Cold Temperatures	30](#_Toc202356187)

[Macrohabitat Diversity	31](#_Toc202356188)

[Scoring Macrohabitat Diversity	32](#_Toc202356189)

[Integrated Diversity Score	33](#_Toc202356190)

[Integrated Connectivity Score	34](#_Toc202356191)

[Condition Score	35](#_Toc202356192)

[Naturalness	35](#_Toc202356193)

[Floodplains	36](#_Toc202356194)

[Floodplain Naturalness Index	37](#_Toc202356195)

[Riparian Areas	39](#_Toc202356196)

[Floodplain and Riparian Naturalness	39](#_Toc202356197)

[Integrating Floodplain-Riparian and Floodplain-Only Naturalness	40](#_Toc202356198)

[Watershed Naturalness	42](#_Toc202356199)

[Integrating Floodplain, Riparian, and Watershed Naturalness	43](#_Toc202356200)

[Water Quality	44](#_Toc202356201)

[Nutrients and Sediment	44](#_Toc202356202)

[Surface Mining Penalty	48](#_Toc202356203)

[Percent Mining Area	49](#_Toc202356204)

[Impervious Surface Override	51](#_Toc202356205)

[Integration of Condition Metrics	53](#_Toc202356206)

[Integrated Physical Score	54](#_Toc202356207)

[Water Score	55](#_Toc202356208)

[Potential Water Availability	56](#_Toc202356209)

[Surface Water Index	56](#_Toc202356210)

[Mapping Surface Water	57](#_Toc202356211)

[Persistent Snowpack	59](#_Toc202356212)

[Integrated Surface Water Index	60](#_Toc202356213)

[Groundwater Adjustments	62](#_Toc202356214)

[Springs and Seeps Adjustment	62](#_Toc202356215)

[Perennial Streams & Rivers Adjustment	63](#_Toc202356216)

[Groundwater Depletion Adjustment	64](#_Toc202356217)

[Hydrologic Landscape Regions	65](#_Toc202356218)

[Groundwater Depletion Analysis	66](#_Toc202356219)

[Groundwater Depletion Metric	66](#_Toc202356220)

[Integrated Map of Potential Water Availability	68](#_Toc202356221)

[Flow Alteration	69](#_Toc202356222)

[Integrated Water Score	70](#_Toc202356223)

[Integrated Freshwater Resilience Score	72](#_Toc202356224)

[Best in Ecoregion	74](#_Toc202356225)

[Macrohabitat Components	77](#_Toc202356226)

[Size	77](#_Toc202356227)

[Gradient	79](#_Toc202356228)

[Local Temperature	80](#_Toc202356229)

[Tidal	81](#_Toc202356230)

[Lakes	81](#_Toc202356231)

[Calculating Macrohabitat Diversity	83](#_Toc202356232)

[Mapping Hydrologic Landscape Region Groundwater Potential	86](#_Toc202356233)

[Subsurface Permeability	86](#_Toc202356234)

[Surface Permeability: Deep Sandy Soils	89](#_Toc202356235)

[Deriving Groundwater Potential Classes	91](#_Toc202356236)

[Estimated Mining Areas	93](#_Toc202356237)

[Freshwater Conservation Status	97](#_Toc202356238)

[General Model for Conservation Status of Freshwater Networks	97](#_Toc202356239)

[Protection Class	98](#_Toc202356240)

[Local Protection	99](#_Toc202356241)

[Protection of Freshwater Provided by Upstream Conservation Lands	100](#_Toc202356242)

[Definition and Categories of Conservation Land	101](#_Toc202356243)

[Regulating Function	103](#_Toc202356244)

[Protection Class Scoring	105](#_Toc202356245)

[Calculating Conservation Status	107](#_Toc202356246)

[Conservation Status for all Flowlines	107](#_Toc202356247)

[Conservation Status of the Freshwater Resilient and Connected Network	109](#_Toc202356248)

[Recognized Biodiversity Data Sources	113](#_Toc202356249)

[Steering Committee	117](#_Toc202356250)

[Supplementary References	119](#_Toc202356251)

List of Figures

[Figure S1. Freshwater Resilient and Connected Network	2](#_Toc202356252)

[Figure S2. Climatic Regions.	3](#_Toc202356253)

[Figure S3. Freshwater Resilience.	4](#_Toc202356254)

[Figure S4. Recognized Biodiversity.	5](#_Toc202356255)

[Figure S5. Freshwater Opportunity	6](#_Toc202356256)

[Figure S6. Protection Class	7](#_Toc202356257)

[Figure S7. Resilient and Connected Network by Resilience and Protection Class	8](#_Toc202356258)

[Figure S8. Freshwater resilience model for the humid region.	10](#_Toc202356259)

[Figure S9. Freshwater resilience model for arid, non-xeric, and perennial-dominated systems.	11](#_Toc202356260)

[Figure S10. Freshwater resilience model for arid, non-xeric, and intermittent-dominated systems.	12](#_Toc202356261)

[Figure S11. Freshwater resilience model for arid, xeric, and perennial-dominated systems.	13](#_Toc202356262)

[Figure S12. Freshwater resilience model for arid, xeric, and intermittent-dominated systems.	14](#_Toc202356263)

[Figure S13. River dams.	17](#_Toc202356264)

[Figure S14. Functionally Connected Network original size.	18](#_Toc202356265)

[Figure S15. Cumulative-discounted FCN length.	20](#_Toc202356266)

[Figure S16. Reservoir penalty.	21](#_Toc202356267)

[Figure S17. Reservoir position.	22](#_Toc202356268)

[Figure S18. HUC-12 percent headwater connectivity examples.	23](#_Toc202356269)

[Figure S19. HUC-12 headwater connectivity classes.	24](#_Toc202356270)

[Figure S20. FCN headwater connectivity classes.	25](#_Toc202356271)

[Figure S21. HUC-12 units adjusted for functional connectivity by type of adjustment.	27](#_Toc202356272)

[Figure S22. Final functional connectivity scores.	28](#_Toc202356273)

[Figure S23. Adjustment in mean summer stream temperature.	30](#_Toc202356274)

[Figure S24. Final temperature model.	31](#_Toc202356275)

[Figure S25. Macrohabitat diversity score.	33](#_Toc202356276)

[Figure S26. Diversity score.	34](#_Toc202356277)

[Figure S27. Final connectivity score.	35](#_Toc202356278)

[Figure S28. HUC-12 watershed 100-year floodplain percentage.	37](#_Toc202356279)

[Figure S29. Land cover naturalness index of the 100-year floodplain.	38](#_Toc202356280)

[Figure S30. Land cover naturalness index of the 100-year floodplain-riparian area.	40](#_Toc202356281)

[Figure S31. Integration of floodplain-only and floodplain-riparian land cover naturalness.	42](#_Toc202356282)

[Figure S32. Land cover naturalness index of HUC-12 watersheds.	43](#_Toc202356283)

[Figure S33. Integrated watershed and floodplain-riparian naturalness scores.	44](#_Toc202356284)

[Figure S34. Cumulative yield maps for SPARROW total nitrogen, total phosphorus, and suspended sediment, and their integration.	47](#_Toc202356285)

[Figure S35. Cumulative water quality index.	48](#_Toc202356286)

[Figure S36. Mining penalty deducted from cumulative water quality score.	50](#_Toc202356287)

[Figure S37. Cumulative water quality score with mining penalty.	51](#_Toc202356288)

[Figure S38. HUC-12 watersheds shown by average 2019 NLCD Percent Developed Imperviousness in five impact classes.	52](#_Toc202356289)

[Figure S39. HUC-12 watersheds with greater than 20% impervious surface.	53](#_Toc202356290)

[Figure S40. Final condition score.	54](#_Toc202356291)

[Figure S41. Physical score.	55](#_Toc202356292)

[Figure S42. Hydrologic processes (Tarboton 2003).	57](#_Toc202356293)

[Figure S43. Inputs to the surface water index.	58](#_Toc202356294)

[Figure S44. Areas of persistent snowpack.	60](#_Toc202356295)

[Figure S45. Surface water index.	62](#_Toc202356296)

[Figure S46. Spring density increase.	63](#_Toc202356297)

[Figure S47. Perennial streams and rivers adjustment.	64](#_Toc202356298)

[Figure S48. Hydrologic landscape regions (HLRs).	65](#_Toc202356299)

[Figure S49. Shallow groundwater depletion categories.	67](#_Toc202356300)

[Figure S50. Areas receiving the shallow groundwater depletion penalty.	68](#_Toc202356301)

[Figure S51. Potential water availability.	69](#_Toc202356302)

[Figure S52. Flow alteration score.	70](#_Toc202356303)

[Figure S53. Stratification regions for the water score.	71](#_Toc202356304)

[Figure S54. Water score.	72](#_Toc202356305)

[Figure S55. Climatic regions and variable influence.	73](#_Toc202356306)

[Figure S56. Areas increased to “Slightly Above Average” resilience score based on an ecoregional override.	75](#_Toc202356307)

[Figure S57. Final freshwater resilience score.	76](#_Toc202356308)

[Figure S58. The River Continuum Concept from Vannote et al. 1980 (FISRWG 1998).	78](#_Toc202356309)

[Figure S59. Illustration of how gradient shapes riverine habitats (FISRWG 1998).	79](#_Toc202356310)

[Figure S60. Macrohabitat components.	82](#_Toc202356311)

[Figure S61. Weighting factors for macrohabitat diversity score.	85](#_Toc202356312)

[Figure S62. Subsurface permeability: bedrock and deep surficial aquifer type	88](#_Toc202356313)

[Figure S63. Subsurface permeability summary classes.	89](#_Toc202356314)

[Figure S64. Deep surficial sandy sediment texture type.	90](#_Toc202356315)

[Figure S65. Surface permeability summary classes.	91](#_Toc202356316)

[Figure S66. Conservation status is defined by two axes, resilience and protection.	98](#_Toc202356317)

[Figure S67. Freshwater protections fall along a spectrum of management intensity and restriction. (Flitcroft et al. 2023).	99](#_Toc202356318)

[Figure S68. American Rivers Protected Rivers Index applied to full stream and river reaches.	100](#_Toc202356319)

[Figure S69. Conservation lands in the conterminous U.S. shown by protection class.	102](#_Toc202356320)

[Figure S70. Regulating function protection provided by conservation lands in a reach’s upstream drainage area.	105](#_Toc202356321)

[Figure S71. Protection class for streams and rivers in CONUS.	106](#_Toc202356322)

[Figure S72. Conservation status of all streams in CONUS based on freshwater resilience and level of protection.	108](#_Toc202356323)

[Figure S73. The distribution of conservation status of all flowlines in CONUS.	109](#_Toc202356324)

[Figure S74. Conservation status of the Freshwater Resilient and Connected Network (FRCN) shown by resilience and protection class.	110](#_Toc202356325)

[Figure S75. The distribution of conservation status of the FRCN in CONUS.	112](#_Toc202356326)

List of Tables

[Table S1. FCN national size classes.	18](#_Toc202356327)

[Table S2. Assignment of HUC-12 headwater connectivity values to z-scores.	23](#_Toc202356328)

[Table S3. Assignment of FCN headwater connectivity values to z-scores.	25](#_Toc202356329)

[Table S4. Final FCN size class z-scores.	27](#_Toc202356330)

[Table S5. Temperature classes in Celsius and Fahrenheit.	29](#_Toc202356331)

[Table S6. Land cover class assignment used in the floodplain and riparian naturalness index.	38](#_Toc202356332)

[Table S7. Data sources for surface water features and modifiers in the surface water index.	58](#_Toc202356333)

[Table S8. Surface water inputs and relative weighting scheme.	61](#_Toc202356334)

[Table S9. Weights for combining the physical and water scores by climatic region.	73](#_Toc202356335)

[Table S10. Length and percentage of each resilience category.	76](#_Toc202356336)

[Table S11. River reach size classes.	78](#_Toc202356337)

[Table S12. Gradient classes.	80](#_Toc202356338)

[Table S13. Temperature classes in Celsius and Fahrenheit.	80](#_Toc202356339)

[Table S14. Macrohabitat component class summary.	81](#_Toc202356340)

[Table S15. Resultant groundwater potential class from the combination of surface and subsurface permeability class.	92](#_Toc202356341)

[Table S16. Conservation land categories used in this analysis.	102](#_Toc202356342)

[Table S17. Weighting of conservation land types by assessment unit to calculate the regulating function potential.	104](#_Toc202356343)

[Table S18. Integration of the regulating function component metrics.	104](#_Toc202356344)

[Table S19. Percent of stream miles by protection class and by stream size.	106](#_Toc202356345)

[Table S20. Conservation status levels for all streams.	107](#_Toc202356346)

[Table S21. All flowline conservation status percentages summarized by protection and resilience class and stream size.	108](#_Toc202356347)

[Table S22. FRCN conservation status percentages summarized by protection and resilience class and stream size.	111](#_Toc202356348)

[Table S23. State plans and analyses of freshwater biodiversity.	113](#_Toc202356349)

[Table S24. Sources for species data in Recognized Biodiversity Value.	116](#_Toc202356350)

# Large Format Maps from Main Paper

All Supplemental Materials maps were created with ArcGIS Pro 3.5.2 (ESRI, Redlands, CA). The hillshade used in all Supplemental Materials maps is from World Hillshade (Sources: Esri, Airbus DS, USGS, NGA, NASA, CGIAR, N Robinson, NCEAS, NLS, OS, NMA, Geodatastyrelsen, Rijkswaterstaat, GSA, Geoland, FEMA, Intermap, and the GIS User Community), except for Figure S1 which uses a public domain hillshade from Herwig G. Schutzler, 1965, Shaded Relief Archive, [https://shadedreliefarchive.com/usa-schutzler.html](https://url.us.m.mimecastprotect.com/s/yDDoCERXyls39yEAqHNf0S7Ldym?domain=shadedreliefarchive.com).

Figure S1. Freshwater Resilient and Connected Network

Figure S2. Climatic Regions.

Figure S3. Freshwater Resilience.

Figure S4. Recognized Biodiversity.

Figure S5. Freshwater Opportunity

Figure S6. Protection Class

Figure S7. Resilient and Connected Network by Resilience and Protection Class

# Model Diagrams

This section provides full-page figures that detail the model components for each climatic region used in the freshwater resilience analysis. Diagrams are available for the following five regions:

- Humid ecoregions
- Arid, non-xeric, perennial-dominated systems
- Arid, non-xeric, intermittent-dominated systems.
- Arid, xeric, perennial-dominated systems.
- Arid, xeric, intermittent-dominated systems.

Figure S8. Freshwater resilience model for the humid region.

Figure S9. Freshwater resilience model for arid, non-xeric, and perennial-dominated systems.

Figure S10. Freshwater resilience model for arid, non-xeric, and intermittent-dominated systems.

Figure S11. Freshwater resilience model for arid, xeric, and perennial-dominated systems.

Figure S12. Freshwater resilience model for arid, xeric, and intermittent-dominated systems.

# Mapping Freshwater Resilience

In this section we present the model for estimating freshwater resilience. For each component, we explain the metrics calculated and how they were integrated to assess the resilience of each HUC-12.

Our model reflects the hypothesis that the physical setting and its condition, together with water availability and alteration, drive freshwater resilience by providing options and the integrity to maintain ecological function (Paukert et al. 2021). The resilience score estimates the relative capacity of a HUC-12 watershed to maintain species diversity and ecological function as the climate changes. The building blocks of the resilience score are connectivity and condition, which were combined to create a physical score; and potential water availability and flow alteration, which were combined to create a water score. The physical and water scores were then integrated to calculate freshwater resilience.

## Physical Score

In this section we explain how the physical score was derived. First, we describe the components of connectivity and describe how they were integrated. Next, we explain how the condition score was calculated from its component metrics and integrated into an index. Finally, we describe how the connectivity score was integrated with the condition score to calculate the physical score.

Our basic model was as follows:

*Physical Score = Connectivity + Condition*

*Connectivity = Functional Connectivity + Diversity*

*Condition = Naturalness + Water Quality + Impervious Surface Override*

### Connectivity Score

The connectivity score was comprised of two components, functional connectivity and diversity. The following sections detail how we defined, mapped, and assessed these two elements.

#### Functional Connectivity

In this analysis, functional connectivity refers to the longitudinal connectivity of a river network accounting for the location of barriers, the passability of those barriers, the detrimental influence of reservoirs, and the benefits of connection to the Great Lakes and to oceans. Larger and more complex networks provide greater access to life-stage habitat needs, refugia, and unimpaired riverine processes. Longitudinal connectivity matters to the resilience of riverine ecosystems because it provides access to habitat needs at various life-history stages, refugia, and unimpaired riverine processes (Tickner et al. 2020). Connectivity within a network of streams is essential to support ecosystem processes and recovery from disturbances. It enables water flow, sediment and nutrient regimes to function naturally, allows species to move throughout the network to find feeding and spawning habitat, and, in times of stress, enables individuals to relocate in pursuit of more suitable conditions for survival (Pringle 2001). Likewise, disruptions in hydrologic connectivity have had negative ecological impacts on several aquatic faunal groups including migratory fish and native freshwater mussels (Pringle 2003).

We measured the size of each Functionally Connected Network (FCN) using area to estimate the amount of connected freshwater habitat. We used area instead of river length because tests showed that area better approximated the true size and length of the connected freshwater network, including all rivers, headwaters, creeks, lakes, ponds, and connected wetlands. Specifically, area is highly correlated with the full network length, including all headwaters and intermittent streams. This approach also allowed us to integrate a suite of important soil and land use processes influencing the health of the connected river network and was not affected by issues arising from stream mapping and digitizing. All else being equal, the larger the connected network, the more likely it is to be resilient because large networks are likely to provide more options for species to move in response to changes in temperature and precipitation and stay within their physiological tolerances (Timpane-Padgham et al. 2017). Connected river networks provide species the ability to disperse to avoid negative impacts of climate change (Comte et al. 2014; Paukert et al. 2021).

##### Delineating FCNs

We used Python scripts developed using Esri’s arcpy package, in combination with all verified river dams, to generate FCNs and calculate the total length and area of each FCN (Figure S13). The scripts used a barrier dataset (points) to split a river network (polylines) into connected networks with unique ID's. We determined the land area draining directly into each FCN using ArcPro’s Watershed Delineation tool and the NHDPlus flow direction grid. The resultant watershed grid provided the drainage area of each unique FCN. The watersheds were then converted to polygons and the area of each FCN was calculated.

Figure S13. River dams.

This map shows the 7,399 river dams used to define FCN boundaries.

###### FCN Size Class

The size of the FCNs was highly skewed towards small values, but also contained a few huge outliers. To represent the variation in FCN size, we used natural breaks (Jenks) to guide our classification of each FCN into one of 11 national size classes (Table S1 and Figure S14).

Table S1. FCN national size classes.

| Size Class | Name | Area (km^2^) |
| --- | --- | --- |
| 1 | Enormous | >100,000 |
| 2 | Huge | >50,000 – 100,000 |
| 3 | Very large | >25,000 – 50,000 |
| 4 | Large | >10,000 – 25,000 |
| 5 | Largish | >5,000 – 10,000 |
| 6 | Moderate | >2,500 – 5,000 |
| 7 | Smallish | >1,000 – 2,500 |
| 8 | Small | >500 – 1,000. |
| 9 | Very small | >250 – 500 |
| 10 | Tiny | <=250 |
| 11 | Minuscule | < 50 |

Figure S14. Functionally Connected Network original size.

Size (km^2^) of the local watershed area of each Functionally Connected Network bounded by dams, coast, or upper extent of headwaters draining to these river segments.

We then adjusted an FCN’s size class to account for the following characteristics which altered the realized size of the network, and thus the availability of habitat to aquatic organisms:

- Dam passability (adjusted upward for partially passable dams)
- Great Lakes or ocean connection (adjusted upward for increased area)
- Reservoirs (adjusted downward for decreased traversability)
- Headwater fragmentation (adjusted downward for decreased headwater connection)

###### Accounting for Partially Passable Dams

Our dataset of river dams included 780 partially passable dams that allow AOP under some conditions. To account for this, we calculated a cumulative discounted length for each FCN bounded by a permeable dam to provide an estimate of the increase in network length. For a given FCN, the cumulative discounted length was calculated by multiplying the permeability of the dam by the length of the FCN on the other side of the dam and continuing in an iterative fashion, multiplying the area of each FCN by the product of all passability scores between it and the given FCN, until a full barrier, coastline, or upper headwater bound is reached (Figure S15). Thus, a large FCN immediately adjacent to a given FCN will have more influence on the cumulative discounted value than a large FCN that is accessed via multiple partially passable barriers. The resulting estimated increase is less than if the dam was removed but reflects a proportionally weighted increase in potential access to the networks upstream or downstream.

To estimate the *area* of the network described by the cumulative discounted length, we used the relationship between the FCN watershed area and the length of FCN rivers plus creeks (flowlines > 10 km^2^ drainage area) by fitting a regression model to the datasets (adj R^2^ = 0.89, p< 0.0001):

*Area (log km) = 1.3 + 0.979 FCN length in log km*

We then evaluated the size class of the predicted watershed area. In many cases, the small increase in accessible area was not enough to place the FCN into a larger size class, but in some cases the FCN moved up one or two size classes. We capped the increase at two size classes after inspecting the few FCNs that received more than a two-size class increase.

Figure S15. Cumulative-discounted FCN length.

The left network comprises five FCNs defined by four full-barrier dams. The right network shows how the cumulative discounted length was calculated when three of the dams were partially passable.

###### Connectivity to Large Water Bodies

We made further adjustments to the FCN size classes to reflect terminal connections to the Great Lakes or the ocean and the ability of many aquatic organisms to move through these environments.

- Great Lakes: All FCNs connected to the Great Lakes were moved to the largest size class to represent how these networks interact with a larger freshwater system.
- Oceans: We used the following rules, based on the median or mean size of all ocean-connected FCNs, to determine the appropriate size class for ocean-connected FCNs. While we increased the size class of these FCNs, they were not all moved to the largest size class as we did for the Great Lakes-connected FCNs, due to the traversability impediment for freshwater species not adapted to move through saline environments.

1. If already in size class 3 or larger (>25,000 km^2^), no change in size class
2. If already in size class 4 (>10,000-25,000 km^2^), move to size class 3
3. If greater than the mean size (2,000 km^2^) and less than 10,000 km^2^, move to size class 4
4. If greater than the median size (300 km^2^) and less than the mean (2,000 km^2^), move to size class 5
5. If less than the median size (300 km^2^), move to size class 6

###### Reservoir Penalty

Reservoirs create challenges for movement, especially in regions without large natural lakes. For freshwater biota adapted to riverine habitats, large reservoirs may present a significant obstacle to the movement of many species or disrupt reproductive strategies that require long, continuous streams for broadcast reproductive strategies (Gido et al. 2010). Additionally, reservoirs are often stocked with predatory invasive species which can have a negative effect on native biota (Juracek 2015), as can the recreational use of motorized boats. However, reservoir habitat is highly variable and sometimes may provide important refugia or connecting tissue especially during times of drought for freshwater biota.

We applied a traversability penalty to the size class of FCNs that contained a large reservoir to account for the increased difficulty freshwater biota could experience moving through large reservoir habitat. We only applied this penalty in ecoregions where large deep lakes do not naturally occur (Figure S16), and we limited it to large deep reservoirs over 1,000 acres in size or reservoirs over 30 feet deep and at least 500 acres in size.

Free-flowing v. dammed reaches of the Red River, Texas

In ecoregions with many natural lakes, we assumed that the freshwater biota evolved with the interspersion of large waterbodies. To identify these ecoregions, we selected freshwater and terrestrial ecoregions where no natural large and deep natural lakes occurred using the “origin” attribute for lakes in the National Lake Assessment dataset (EPA 2012).

Figure S16. Reservoir penalty.

The reservoir penalty was applied in the freshwater ecoregions shown in light brown because they had no natural large and deep lakes.

We calculated the reservoir penalty as a function of the reservoir position within the FCN and the orientation of incoming river(s). An example of a reservoir with minimal impact on connectivity would be one located at the base of an FCN where the total length of the longest incoming river above the reservoir was very close to the total length of the FCN (Figure S17 example 1). An example of a high impact reservoir would be one where the location of the reservoir and incoming tributaries fragments the total FCN length into many small segments (Figure S17 example 2). In the latter case, the longest riverine length above the reservoir would be a small percentage of the total FCN length. For every FCN that contained a reservoir, we calculated the length of the largest lobe above the reservoir and then the percentage of the total FCN length accounted for by the lobe. If the longest reservoir lobe was less than 70% of the total FCN size, we lowered the FCN size class by one class.

Figure S17. Reservoir position.

In example 1, the reservoir has a minimal impact because the longest river network above the reservoir is 90% of the previously connected riverine FCN length. In example 2, the reservoir has a high impact on connectivity because the longest river lobe above the reservoir is only 50% of the total connected riverine FCN length.

##### **Headwater Fragmentation**

The headwater fragmentation metric expresses how much of the headwater area of a HUC-12 and an FCN is likely to be connected to its rivers. Headwater connectivity is critical to protecting and maintaining downstream ecological integrity (Alexander et al. 2018). We used this analysis to adjust the FCN size, lowering the size class by one class if the headwaters were highly fragmented (“Below Average” or “Far Below Average” score). The analysis used the 58,000 dams falling on headwater streams, which we did not review for accuracy or location. We used the number and configuration of small headwater dams to compare the drainage area upstream of the small dams to the total area of the FCN to provide an estimate of the degree to which small dams are likely decreasing overall headwater connectivity within a network.

###### HUC-12 Headwater Fragmentation

We delineated the upstream drainage area of each dam on headwaters and creeks (draining 100 km^2^ or less), excluding any dams that were used to create the FCNs. We then spatially intersected the HUC-12 watersheds with these upstream dam drainages and calculated the total dam drainage area for each HUC-12. To calculate the percent headwater connectivity, we divided the largest drainage area in each HUC-12 by the total HUC-12 area (Figure S18).

We calculated z-scores by freshwater ecoregion, climatic zone, and CONUS-wide, and for each we assigned the connectivity values to seven classes using natural breaks (Table S2). After reviewing maps of the z-score and natural break classes, we selected the CONUS natural break classes as the most accurate representation of headwater fragmentation (Figure S19). We then translated the natural break classes to z-scores (Table S2).

Figure S18. HUC-12 percent headwater connectivity examples.

Watersheds in red are the most fragmented by dams on headwaters and creeks, while watersheds in green have no stream dams or the location of stream dams has a minimal impact on headwater connectivity.

Table S2. Assignment of HUC-12 headwater connectivity values to z-scores.

| **Headwater Connectivity (%)** | **Natural Break Class** | **Z-Score Range** |  | **Z-Score  Class** |
| --- | --- | --- | --- | --- |
| ≤45.53 | 1 | -3 to -2 |  | Far Below Average |
| > 45.53 & ≤ 60.61 | 2 | -2.1 to -1 |  | Below Average |
| > 60.61 & ≤ 72.72 | 3 | -1.1 to -0.5 |  | Slightly Below Average |
| > 72.72 & ≤ 82.64 | 4 | -0.51 to 0.51 |  | Average |
| > 82.64 & ≤ 90.60 | 5 | 0.51 to 1 |  | Slightly Above Average |
| > 90.60 & ≤ 97.04 | 6 | 1.1 to 2 |  | Above Average |
| > 97.04 | 7 | 2.1 to 2 |  | Far Above Average |

Figure S19. HUC-12 headwater connectivity classes.

These natural-break classes were translated to z-scores as shown in Table S2. Watersheds in red are the most fragmented by the location and/or number of stream dams while watersheds in green have no stream dams or the location of stream dams has very little impact on headwater connectivity.

###### FCN Headwater Fragmentation

We calculated percent headwater connectivity for FCNs using the same approach as described above for HUC-12 watersheds. That is, we spatially intersected the stream dam drainages with the FCN watersheds, calculated the total dam drainage area for each FCN, calculated the FCN area not in an upstream dam drainage, and found the largest drainage area in each FCN.

To calculate the percent headwater connectivity metric, we divided the largest drainage area in each FCN by the total FCN area. As for the HUC-12s, we used the CONUS-wide natural break classes and translated those classes to z-scores (Table S3, Figure S20). If FCN headwater connectivity was “Below Average” or “Far Below Average,” we lowered the size class by one class.

Table S3. Assignment of FCN headwater connectivity values to z-scores.

| **Headwater Connectivity (%)** | **Natural Break Class** | **Z-Score Range** | **Z-Score Class** |
| --- | --- | --- | --- |
| ≤ 40.47 | 1 | -3 to -2 | Far Below Average |
| > 40.47 & ≤ 56.02 | 2 | -2.1 to -1 | Below Average |
| > 56.02 & ≤ 68.30 | 3 | -1.1 to -0.5 | Slightly Below Average |
| > 68.30 & ≤ 79.05 | 4 | -0.51 to 0.51 | Average |
| > 79.05 & ≤ 88.52 | 5 | 0.51 to 1 | Slightly Above Average |
| > 88.52 & ≤ 96.34 | 6 | 1.1 to 2 | Above Average |
| > 96.34 | 7 | 2.1 to 2 | Far Above Average |

Figure S20. FCN headwater connectivity classes.

These natural-break classes were translated to z-scores as shown in Table S3. FCN watersheds in red are the most fragmented by the location and/or number of stream dams while watersheds in green have no stream dams or the location of stream dams has very little impact on headwater connectivity.

###### Scoring of HUC-12s not within an FCN

A small number of HUC-12s were not part of any FCN as they did not include a mapped river. Rather than exclude these HUC-12s from our analysis, we chose to assign them to a small to medium size class using the following rules based on their downstream connectivity type and headwater connectivity. High headwater fragmentation refers to HUC-12s that scored “Below Average” or “Far Below Average” for headwater connectivity.

- Ocean connected without high headwater fragmentation = size 7
- Ocean connected with high headwater fragmentation = size 8
- Great Lakes connected without high headwater fragmentation = size 3
- Great Lakes connected with high headwater fragmentation = size 4
- Internal HUCs not connected to a river, without high headwater fragmentation = size 10
- Internal HUCs not connected to a river with high headwater fragmentation = size 11
- Internal HUCs less than 50 km^2^ = size 11

##### Final Functional Connectivity Score

All the foregoing adjustments were used to determine the final functional connectivity score. Figure S21 shows which FCN and HUC-12 units were adjusted for their level of functional connectivity and for what reason (dam passability, large water-body connection, reservoirs, and headwater fragmentation). Table S4 shows how we translated the final size classes based on natural breaks to z-scores. Figure S22 shows the final adjusted functional connectivity z-score for all FCNs.

Figure S21. HUC-12 units adjusted for functional connectivity by type of adjustment.

Table S4. Final FCN size class z-scores.

| **Size  Class** | **Area (km^2^)  Thresholds** | **Z-Score** |
| --- | --- | --- |
| 1 | >100,000 | 3.0 |
| 2 | >50,000 - 100,000 | 3.0 |
| 3 | >25,000 - 50,000 | 2.3 |
| 4 | >10,000 - 25,000 | 1.7 |
| 5 | >5,000 - 10,000 | .99 |
| 6 | >2,500 - 5,000 | 0.5 |
| 7 | >1,000 - 2,500 | -0.51 |
| 8 | >500 - 1,000 | -1.01 |
| 9 | >250 - 500 | -1.7 |
| 10 | <=250 | -2.3 |
| 11 | < 50 | -3.0 |

Figure S22. Final functional connectivity scores.

This map shows the functional connectivity score for each FCN based on the original size of the riverine network adjusted for passable dams, connectivity to the ocean or Great Lakes, large reservoirs, headwater fragmentation, and for HUCs not within FCNs.

#### Diversity

The diversity score measures the physical habitat variation in an FCN. Greater connectivity increases the opportunity for dispersal, while variation in connectivity among multiple stream sizes and major habitats increases resilience and metapopulation stability (Pelletier et al. 2020). In other words, the connectivity of a variety of habitats in an FCN gives organisms options to move in response to seasonal and interannual changes in the ambient environment. Not only does this include connectivity across scales, but also the availability of diverse habitats for various life-history stages and to buffer aquatic systems from physical disturbance or invasive species, creating an insurance effect (Pelletier et al. 2020). This is important in a changing climate for species moving north or up in elevation seeking cooler waters (Comte & Grenouillet 2013), but also for species in prairie streams, such as in the Great Plains, as it maintains connectivity to larger order streams with more abundant perennial water (Dodds et al. 2004). When accessible, a diversity of temperatures, gradients, size classes, and habitats creates options for species to find suitable habitat as conditions change. For example, an FCN with a range of temperatures, especially cooler or colder waters, can provide habitat and refugia for species able to access them as temperatures increase.

##### Temperature Diversity

Stream temperature is fundamental to freshwater resilience because it sets the physiological limits of persistence for many organisms, and temperature extremes may directly preclude certain taxa from inhabiting a water body. Seasonal changes in water temperature often cue development or migration, and temperature can influence growth rates and fecundity. Ideally, a resilient stream network would span a range of current temperatures, offering options for both coldwater and warmwater species and providing connected habitats for species to stay within their thermal preferences in the future (Woodward et al. 2010). For each FCN, we calculated the diversity of temperature classes and gave more weight to areas with persistent cold water.

Mean summer stream water temperature modeled data was available for most reaches from the EPA’s Stream-Catchment (StreamCat) dataset (Hill et al. 2016), a national source widely used to study reference temperatures. The StreamCat model is based on stream gage data from 1999-2008 and fits a single, nation-wide predictive model to the data by applying variable selection techniques to potential variables. The final model used six predictors: mean summer air temperature, watershed area, base-flow index, mean stream channel slope, soil bulk density, and elevation ranges within each watershed. The model predicts stream temperature by accounting for differences in the physical environments among streams.

We extracted the mean summer stream temperature variable (MSST) to represent the predicted reference-condition mean summer stream temperatures. The missing data for streams in northern Michigan was filled in using Lyons et al. (2009). Temperature predictions were not available for very large rivers (>100,000 km^2^) because they were not represented in the MSST gage dataset.

###### Temperature Classes

The continuous temperature predictions were lumped into five summer classes following McManamay et al. (2019), except that we lumped the two coldest classes for which there were few reaches (Table S5). The temperature class breaks matched closely to those used in other stream and river classifications developed in Michigan (Lyons et al. 2009), the Appalachians (Olivero-Sheldon et al. 2015), and the Eastern U.S (McManamay et al. 2018).

Table S5. Temperature classes in Celsius and Fahrenheit.

| **Temperature Class** | **Celsius** | **Fahrenheit** |
| --- | --- | --- |
| Very Cold | <15 | <59 |
| Cold | >15-18 | 59-64.4 |
| Cool | >18-21 | 64.4-69.8 |
| Cool-Warm | >21-24 | 69.8-75.2 |
| Warm | >24C | >75/.2 |

###### Weighting for Cold Temperatures

The FCN represents the accessible stream network for populations to use and move within. Given stream temperatures are warming from climate change, our temperature metric incorporated a count of the diversity of temperature classes present in the FCN with more weight given to colder temperature classes. A temperature class was counted if it made up more than 5% of the network, exclusive of very small headwater streams (less than 10 km^2^). The classes were weighted as follows to form an index of temperature diversity:

*Temperature Diversity = (5*Very Cold) + (4*Cold) + (3*Cool) + (2*Cool-Warm) + (1*Warm)*

These scores were transformed into z-scores and an additional 0.5 SD increase was added to the score if modeling indicated the lowest temp class was maintained in a future that was 2.2°C warmer. An increase of 2.2°C is the rise in average summer stream temperature predicted for U.S. streams by 2100 (Hill et al. 2014). To calculate this, we added 2.2 degrees C to the reference predicted MSST stream temperatures of each flowline (Figure S23) and recalculated the presence and proportions of each class in the FCN in this new scenario (Figure S24).

Figure S23. Adjustment in mean summer stream temperature.

Left map shows the temperature reference condition estimated from the EPA StreamCat model. Right map shows the model results with a 2.2^0^ C rise in temperature.

Figure S24. Final temperature model.

This map shows the z-scores of the cold-weighted temperature index and the 0.5 SD persistent cold bump.

##### Macrohabitat Diversity

Macrohabitats are the finest scale unit of hierarchical aquatic classification types defined in TNC’s freshwater classification approach (Higgins et al. 2005). They define stream reach types or lake types and are based on key abiotic variables known to structure aquatic communities at the reach or lake scale. For this analysis, we created simplified macrohabitat types to represent variation in major aquatic physical habitats at the stream reach or individual lake scale using variables possible to model in GIS. Our hypothesis was that HUC-12 units with more macrohabitats would provide more options for species to adapt to change and thus support resilience.

Development of the macrohabitats was guided by the variables and ecologically relevant classes included in the national stream classification developed by McManamay and DeRolph (2019) and related studies on physical freshwater classification and conservation assessment (McManamay et al. 2018; Higgins et al. 2005). Lotic macrohabitats were calculated at the HUC-12 scale based on unique combinations of stream size (7 classes), gradient (5 classes), temperature (5 classes), and tidal influence (2 classes). Stream size is indicative of major ecosystem changes along the stream-river continuum, such as transitions in the state and source of energy and ecosystem metabolism (Vannote et al. 1980). Stream gradient also highly influences aquatic communities at the reach scale due to its influence on stream bed morphology, flow velocity, sediment transport/deposition, substrate, and grain size (Rosgen 1994). Local temperature was included to account for physiological limits of persistence for many organisms. Tidal influence and lake types were also counted as providing unique habitat diversity. Lake macrohabitats were based on depth to represent major differences in the probability of the presence of cold water in the summer (Olivero-Sheldon & Anderson 2016). A detailed explanation and maps of each macrohabitat component can be found later in this Supplemental Appendix

###### Scoring Macrohabitat Diversity

The final macrohabitat diversity score for each HUC-12 unit was calculated as the number of macrohabitat types, relative to the average number of macrohabitats expected for the size of the HUC. Because the count of macrohabitats was correlated with HUC size, we used a Poisson regression to establish a relationship between the size of the HUC and the count of macrohabitats, and then calculated the standardized residuals to identify HUCs that had more or less macrohabitat diversity than expected for their size. The standardized residuals were used as the z-score.

We made two adjustments to the scores. First, to account for the larger effect of perennial macrohabitats on the available diversity, we calculated z-scores for all streams and for perennial streams only. We combined the two scores, giving equal weight to each, and thus more weight to perennial streams, which were included in both.

*Macrohabitat Diversity = (standard residual all streams + standard residual perennial only)/ 2*

Small dams on headwaters and creeks decrease the accessibility of all types of macrohabitats within a HUC-12. In HUCs with “Average” or worse headwater fragmentation, we adjusted the macrohabitat diversity score by taking the average of the combined macrohabitat diversity z-score and the headwater fragmentation z-score. Finally, we integrated all the components and penalties into a single weighted map of macrohabitat diversity for CONUS (Figure S25).

Figure S25. Macrohabitat diversity score.

This map reflects all components and weightings.

##### Integrated Diversity Score

*Diversity Score = (Temperature score + Macrohabitat Diversity score) / 2*

The final diversity score combined the temperature score at the FCN scale and the macrohabitat score at the HUC-12 scale (Figure S26). We combined the two scores at the HUC level, giving equal weight to both scores. In the rare cases where HUCS were not within an FCN (e.g., some coastal watersheds or internally draining basins), the diversity score was based only on the macrohabitat diversity score.

Figure S26. Diversity score.

This map shows the integration of the FCN temperature diversity and the HUC-12 macrohabitat diversity scores.

#### Integrated Connectivity Score

*Connectivity Score = (2*Functional Connectivity + 1*Diversity) / 3*

Our final step was to integrate the functional connectivity score measured at the FCN scale with the diversity score measured at the HUC-12 scale (Figure S27). The aim was to be able to assess the size and diversity of a network with a single index that expresses the complexity of the available habitats. While each factor contributes to network resilience, they are not fully independent because functional connectivity is based on size of the network and diversity is also highly correlated with size. To account for this, we assessed macrohabitat diversity based on the departure from expected diversity when compared to that predicted from size alone. However, given the importance of network size to the availability of habitats, we gave functional connectivity twice the weight as diversity in our combined index.

Figure S27. Final connectivity score.

In this map, the size of the network is given twice the weight as the diversity of the network.

### Condition Score

*Condition = Naturalness + Water Quality adj. for Surface Mining + Impervious Surface Override*

Condition is a measure of current ecological function from landscape influences and accounts for two key ecological attributes of freshwater systems, habitat quality and water quality. This analysis was stratified by arid and humid regions.

#### Naturalness

*Naturalness = Floodplain and/or Riparian Naturalness + Watershed Naturalness*

In this section, we assess the naturalness of three essential river features, the floodplain, riparian area, and watershed. For each feature, we first developed a map of its extent and then overlaid land cover data to quantify the types and amounts of natural, agricultural, and developed land within the extent.

##### **Floodplains**

Connections between a river and its floodplain are essential to the resilience of the system because floodplains allow for water storage, nutrient flushes, and create spatial and temporal feeding and breeding habitat (Pringle 2003). The connection can be interrupted by roads and levees, although this can be difficult to precisely measure because it depends in part on the intensity and depth of each flood event, or the return interval of the flood. We calculated a metric of floodplain naturalness to estimate the relative intactness and availability of the floodplain.

To map floodplains, we compiled all data (10-m GeoTIFFs) representing the 100-year floodplain from the Fathom-U.S. 2.0 dataset (Bates et al. 2021; First Street Foundation 2020) for CONUS. These were merged into a single 10-m raster, taking the maximum value if two grid cells overlapped. We used the “defended” version, meaning that the spatial data accounts for known flood defenses such as levees. We reprojected and resampled the data to a 30-m resolution and snapped it to a base grid to ensure alignment with other grids developed for this analysis. All cells representing modeled water depth values were reclassified to a single value. Permanent water bodies were removed.

To identify watersheds where floodplains were a predominant freshwater feature, we summarized the floodplain area in each HUC-12 and calculated the floodplain percentage in each. We translated the floodplain percentage values to standardized normal scores (z-scores) by arid and humid ecoregions (Figure S28). We used this metric to determine how much weight to give the floodplain naturalness score as described below.

Figure S28. HUC-12 watershed 100-year floodplain percentage.

Watersheds in green have a large area of 100-year floodplain relative to their humid or arid ecoregion (inset map with blue indicating humid ecoregions and lime denoting arid). Watersheds in the Southeastern U.S. coastal plain and parts of the Mississippi River Basin had the highest percentages of floodplain area. There is no floodplain area in the lower Mississippi Alluvial Valley because the data incorporated flood defenses such as levees.

##### Floodplain Naturalness Index

After removing open water from the 100-year floodplain, we quantified the extent of four land cover categories (Table S6) in this zone using data from the National Land Cover Dataset (NLCD, Dewitz & USGS 2021) augmented with surface mining extent (see Surface Mining Penalty section). Land cover classes were weighted such that wetlands or natural forests had the highest positive influence and roads or permanent development had the highest negative influence, with agricultural lands in between. We did not include pasture and hay lands in the agriculture category as research has shown this land use is not strongly correlated with poor water quality (de Mello et al. 2018). Degree of naturalness was quantified using a weighted index:

*(1 * % High Intensity) + (0.75 * % Agriculture) + (0.25 * % Low Intensity)*

The index ranged from 0 for a floodplain in completely natural cover to 100 for a completely developed floodplain. We translated the floodplain naturalness index values to standardized normal scores (z-scores) by arid and humid ecoregions (Figure S29).

Table S6. Land cover class assignment used in the floodplain and riparian naturalness index.

| **Naturalness Category** | **NLCD 2019 Class (Value Code)** |
| --- | --- |
| High Intensity | Low Intensity Developed (22), Medium Intensity Developed (23), High Intensity Developed (24), Mining |
| Agriculture | Cultivated Crops (82) |
| Low Intensity | Pasture/Hay (81), Open Space Developed (21), Barren (31) |
| Natural | Open Water (11), Perennial Ice/Snow (12), Deciduous Forest (41), Evergreen Forest (42), Mixed Forest (43), Shrub/Scrub (52), Herbaceous (71), Woody Wetlands (90), Emergent Herbaceous Wetlands (95) |

Figure S29. Land cover naturalness index of the 100-year floodplain.

Scores were converted to z-scores by humid (blue in inset map) and arid (lime in inset map) ecoregions. Watersheds in green have floodplains in more natural land cover while those in brown are dominated by agricultural and/or developed land uses.

##### **Riparian Areas**

Riparian zones contribute to freshwater resilience through their potential to attenuate the effects of precipitation changes (Paukert et al. 2021), dampen temperature rise impacts (Bowler et al. 2012), and for their ability to retain sediments and nutrients that could degrade water quality (Kaufman et al. 2022). To map riparian areas, we compiled the pluvial data (10-m GeoTIFFs) representing the 100-year flood return interval from the Fathom-U.S. 2.0 dataset (Bates et al. 2021; First Street Foundation 2020) and again merged them into a single 10-m raster, taking the maximum value if two grid cells overlapped. “Pluvial” refers to flooding based on the accumulation of rainfall in topographic depressions and accounting for impervious surfaces and land cover. We reprojected and resampled the data to a 30-m resolution and snapped it to a base grid to ensure alignment with other grids developed for this analysis. All cells representing modeled water depth values were reclassified to a single value. Permanent water bodies were removed to avoid inflating natural areas due to the presence of water.

We considered other data sources to map riparian areas including a fixed distance buffer of stream reaches and the U.S. Forest Service national riparian areas (Abood et al. 2022) but found the Fathom pluvial model provided a consistent and comprehensive delineation of areas likely to be riparian based on topography.

###### Floodplain and Riparian Naturalness

The resampled 30-m grid had unique values to denote if the source was pluvial, fluvial, or both. We classified land cover in the combined fluvial and pluvial area of each HUC-12 into the four categories previously described and shown in Table S6. Using those categories, we calculated the land cover naturalness index as described above in the floodplain naturalness section. We again translated HUC-12 index values into z-scores stratified by arid and humid ecoregions (Figure S30).

Figure S30. Land cover naturalness index of the 100-year floodplain-riparian area.

Scores were converted to z-scores by humid (blue in inset map) and arid (lime in inset map) ecoregions. Watersheds in green have floodplain and riparian areas largely in natural land cover while those in brown are dominated by agricultural and/or developed land use.

##### Integrating Floodplain-Riparian and Floodplain-Only Naturalness

After reviewing the land cover naturalness indices for the two different extents (floodplain-only vs. floodplain-riparian), we established a set of rules to guide their use. In arid ecoregions, given the importance of riparian areas and the lack of large floodplains, particularly those with a high number of intermittent or ephemeral streams and rivers, we always used the floodplain-riparian naturalness score. For watersheds in humid ecoregions, we used the floodplain-only score (Figure S29) when the percentage of floodplain area was “Above Average” or “Far Above Average.” For watersheds in humid ecoregions where floodplains were not a dominant feature (< 1 SD, in the “Slightly Above Average” or lower classes), we used the floodplain and riparian naturalness index score (Figure S30).

Relativization of naturalness index values by arid and humid ecoregions led to some unintuitive patterns, particularly in the Western U.S. Because land is less urbanized and there is less water for row crops, land cover in western floodplain and riparian zones is often quite natural. Thus, a western watershed with an “average” naturalness z-score could be completely natural. While this situation also occurred in humid ecoregions, it was less pronounced. To address this issue, we adjusted the z-scores of HUC-12 watersheds with high floodplain and riparian naturalness scores. Regardless of climatic zone, if the floodplain-only or the floodplain and riparian raw naturalness index score was less than five (highly natural), an increase of 0.5 SD was applied to the respective z-score. If the naturalness index score was greater than or equal to five and less than ten, a smaller increase of 0.25 SD was awarded to the z-scores of those HUC-12s. After making these adjustments, we also adjusted z-scores for humid HUC-12s where floodplains are a critical freshwater component. For humid HUC-12s where the floodplain size was “Far Above Average,” we applied a 0.5 SD bump to the adjusted floodplain-only score if the z-score was “Average” or higher. Conversely, if the floodplain land cover was highly altered (less than “Average”) but comprised a large proportion of the HUC-12 (floodplain size ≥ 2.0 SD, “Far Above Average”), we deducted 0.5 SD from the z-score. For HUC-12 watersheds with an “Above Average” floodplain percentage, we applied a 0.25 SD increase if the naturalness z-score was “Average” or higher and a 0.25 SD penalty if the naturalness z-score was less than “Average.” Figure S31 shows the results of applying these adjustments, summarized below.

Floodplain-Riparian Score (raw scores)

Floodplain or Floodplain/Riparian Score < 5	0.50 SD increase

Floodplain or Floodplain/Riparian Score >= 5 & <10	0.25 SD increase

Floodplain-Only Score (z-scores)	Z-score adjustment

Floodplain size >2 SD & Adjusted Naturalness Score >=0.5 SD	0.50 SD increase

Floodplain size >1 SD & Adjusted Naturalness Score >=0.5 SD	0.25 SD increase

Floodplain size >2 SD & Adjusted Naturalness Score < -0.5 SD	-0.50 SD penalty

Floodplain size >1 SD & Adjusted Naturalness Score < -0.5 SD	-0.25 SD penalty

Figure S31. Integration of floodplain-only and floodplain-riparian land cover naturalness.

Results reflect the integration rules and z-score adjustments described in the text.

##### Watershed Naturalness

To put the floodplain and riparian scores in context and assess the degree of degradation within the whole watershed, we calculated a watershed naturalness score for each HUC-12 based on current landcover using the 2019 NLCD, adjusted for mining see Surface Mining Penalty section) and using the same naturalness index methods as described for the floodplain and riparian zones. We translated the watershed naturalness index values to z-scores by arid and humid ecoregions (Figure S32).

Figure S32. Land cover naturalness index of HUC-12 watersheds.

Scores are converted to z-scores by humid (blue in inset map) and arid (lime in inset map) ecoregions. Watersheds in green have more natural and intact land cover while those in brown are dominated by agricultural and/or developed land use.

##### Integrating Floodplain, Riparian, and Watershed Naturalness

A visual comparison of the combined floodplain and riparian land cover naturalness index and the watershed naturalness index shows there are many watersheds where the two metrics are quite similar (e.g., Mississippi River Basin). However, there are also areas where the two metrics paint a different picture such as in North Carolina’s coastal plain where riverine floodplains are often large and intact, but their watersheds tend to have altered land cover. We explored several different weighting schemes to address the correlation between these two indices and found that simply averaging the two z-scores seemed to appropriately balance the interplay between these different scales. For example, a watershed with a very natural floodplain but a less natural watershed typically received an “Average” overall naturalness score (dependent on the actual z-scores for the two scales). A watershed primarily in natural cover and with a floodplain in natural cover received a high overall naturalness score. Our final integrated score (Figure S33) can be thought of as a watershed naturalness score (Figure S32) with more weight given to the floodplain and riparian zone (Figure S31) to reflect the importance of direct interaction with streams and rivers in this region.

Figure S33. Integrated watershed and floodplain-riparian naturalness scores.

The z-scores are the average of the watershed naturalness z-score and the floodplain only or floodplain and riparian naturalness z-score for each HUC-12. The floodplains and land cover of watersheds in green are more natural while the floodplain and land cover of watersheds in brown are characterized by agricultural and/or developed land uses.

#### Water Quality

*Water Quality = Nutrient and Sediment Index adjusted for Surface Mining*

##### Nutrients and Sediment

Recent studies suggest most U.S. streams and rivers continue to have higher levels of nitrogen and phosphorus than is recommended (Manning et al. 2020). Although these nutrients are a natural part of aquatic ecosystems, human activity has increased their levels beyond what the system can naturally absorb and buffer. At high levels, nutrients can lead to excessive algal growth which can harm water quality, alter food webs and resources, change community composition and diversity, decrease the oxygen that fish and other aquatic life need to survive, and contribute to hypoxia in coastal waters (Frei et al. 2021; Piggott et al. 2015).

Excess sediment is another pollutant persistently found in U.S. streams and rivers (Murphy 2020). In addition to reducing water clarity, excess sediments can bury and suffocate benthic organisms and fish eggs, cover habitat, and disrupt food webs (Robertson & Saad 2019). Sediment can also impact fish by clogging their gills and reducing disease resistance, growth rates, and altering egg and larvae development. Sediment can bind harmful contaminants, and sedimentation of rivers can change stream flow and reduce water depth (MARC 2023). Nutrient and sediment pollution is also impacted by changes in streamflow (Murphy 2020) which can be exacerbated by climate change and dams.

The EPA’s most recent national assessment of streams and rivers in CONUS found 58% were rated poor for phosphorus and 43% were rated poor for nitrogen (USEPA 2020). The study found phosphorus, nitrogen, and suspended sediment had the highest attribute risk values of all the stressors assessed. For example, rivers and streams rated poor for nutrients and sediment levels were almost twice as likely to have poor biological condition based on multi-metric scores for benthic macroinvertebrate assemblages (USEPA 2020).

Sources of nutrient and sediment pollution vary but most are correlated with agricultural practices. Nitrogen is most likely to come from crop fertilizers, wastewater, and animal wastes as well as atmospheric deposition from the burning of fossil fuels. Increased phosphorus is more commonly the result of poor agricultural practices, sewage waste, increased soil erosion, and urban runoff. Sediment sources include runoff from agricultural fields, intensive forestry operations, urban areas, and construction activities.

While enforcement of the Clean Water Act has led to the reduction of point source pollutants in American rivers and streams, nonpoint source pollution, especially from agricultural activities, continues to be a regulatory challenge (Stets et al. 2020; Frei et al. 2021). Crop productivity and nitrogen from fertilizer has grown dramatically in the Midwestern U.S. as have concentrated livestock operations (Falcone et al. 2018). Another notable water quality trend, and one that we did not assess due to insufficient national data, is the emergence of salinity as a rapidly growing threat to freshwater systems (Stets et al. 2020). There are a few bright spots. A recent USGS study of 137 U.S. sites found that in some places, efforts to reduce sediment pollution entering waterways appear to be making a difference (Murphy 2020). Similarly, efforts to control nonpoint source pollution in urbanized areas also appear to be reducing nutrient loads to streams and rivers (Stets et al. 2020).

For these reasons, we considered water quality to be an important component of a resilient freshwater network. Fortunately, USGS SPARROW models provide spatially explicit estimates of nutrient loads, concentrations, and yields for all streams in CONUS. SPARROW uses spatially comprehensive geospatial data in a calibrated model to predict water-quality conditions at unmonitored stream locations. The most recent SPARROW models provide estimated nutrient information for each NHDPlus reach catchment, based on source inputs (e.g., land cover) and management practices existing during or near 2012 (Dewitz and USGS 2021). The streamflow component of the SPARROW models is based on the average input precipitation minus actual evapotranspiration from 2000 to 2014.

We obtained the SPARROW Northeast (Ator 2020), Southeast (Roland and Hoos 2020), Midwest (Saad & Robertson 2020), Southwest (Miller et al. 2020), and Pacific (Wise 2020) model outputs to cover CONUS. South Florida watersheds have significant water diversions and fluxes for which there is insufficient data to model in SPARROW. Thus, this region was excluded from the Southeast SPARROW models and is shown in gray to indicate “No Data” for all water quality maps. We used incremental and accumulated yield values for total nitrogen (TN), total phosphorus (TP), and suspended sediment (SS). Incremental values are the predicted mean annual loads of a stream that are contributed from sources in the immediate drainage area of a stream reach (equal to NHD catchment). Accumulated values are loads contributed by all sources in the total upstream drainage area of the reach outlet and include the effects of in-stream attenuation processes in all upstream reaches. Incremental yield is the incremental load divided by the local catchment area, while accumulated yield is the accumulated load divided by the total upstream drainage area. TN and TP yield units are kilograms per km^2^ per year, and SS yield units are metric tons per km^2^ per year.

The dataset for the Southwest contained duplicate units where catchments crossed state lines. To address this issue, we took the maximum suspended sediment value for duplicate units. We then combined the selected attributes for all SPARROW regions, assigned “no-data” values to closed catchments with no predicted nutrient loads, and examined patterns in the incremental and accumulated yields for each constituent. To capture the cumulative impact of nutrients and sediments on freshwater systems, we used cumulative yields for each constituent as our measures of nutrients and sediments (Figure S34).

Figure S34. Cumulative yield maps for SPARROW total nitrogen, total phosphorus, and suspended sediment, and their integration.

Maps A-C show classes relative to each SPARROW model region (black lines) of cumulative yields for A) TN total nitrogen, B) TP total phosphorus, and C) SS suspended sediment with higher loads denoted with darker colors. D) The integrated water quality index z-scores stratified by arid/humid zones.

We assigned each catchment to its dominant freshwater ecoregion and converted the TN, TP, and SS accumulated yield values to standard normal scores (z-scores) by arid and humid ecoregions, and CONUS. CONUS level z-scores highlighted western watersheds, which typically have lower nutrient and sediment loads relative to midwestern and eastern watersheds. Relativizing results by arid and humid ecoregions resulted in the most logical and interpretable patterns. Regardless of the stratification used, the TN and TP z-scores were often strongly correlated. Thus, for each catchment, we took an average of the TN and TP z-scores and then averaged that score with the SS z-score to calculate a cumulative water quality index for each catchment (Figure S34).

We used an area-weighted approach to translate the cumulative water quality index (Figure S34D) to HUC-12 watersheds. Specifically, we calculated the proportion that each catchment contributed to the total HUC-12 watershed area. We then multiplied the cumulative water quality z-score by catchment proportion and summed the values by HUC-12 (Figure S35).

Figure S35. Cumulative water quality index.

Scores are by HUC-12 watersheds. Black lines indicate the arid and humid climatic regions used to stratify the cumulative water quality yields at the catchment scale (Figure S34).

##### Surface Mining Penalty

Metals, minerals, and rocks are extensively mined in the U.S., with coal, sand and gravel, gold, and copper the top mined commodities. Mining can be detrimental to freshwater systems due to land degradation, depletion of surface and groundwater supplies, stream channel and stream flow alterations, water pollution, alteration of soil and subsurface geologic structure, and disruption of surface and subsurface hydrologic regimes (SDWF 2017; Starnes & Gasper 1995). The primary water quality concerns from mining activities are acid mine drainage, heavy metal contamination and leaching, pollution from processing chemicals, and sedimentation and erosion (SDWF 2017).

While there are a range of both national and regional spatial datasets that identify mine footprints in the U.S., many are outdated, inconsistent, and/or have incomplete geographic coverage. To estimate the potential impact of surface mining activities (e.g., mountaintop removal, quarries, strip mines, open pit mines, dredging) on watersheds, we developed a new dataset of mining footprints in CONUS using current land cover with global, national, regional, and state mining datasets. Here we summarize our approach to identify surface mining areas.

We compiled, compared, and error checked all the data sources and made adjustment until they represented a spatially consistent dataset of mining operations. We converted the selected mining polygons to a 30-m raster and merged all the grids using Cell Statistics in ArcGIS to retain the source datasets for all mapped mining areas. We created a mining area raster where all mining areas were coded as 10 and then we merged this on top of the 2019 NLCD land cover for use in the water quality mining metric and the floodplain, riparian, and watershed land cover naturalness metrics.

##### Percent Mining Area

We tabulated the area of mine land in each HUC-12 watershed and calculated the percentage of mining area in each watershed. After removing HUC-12s with no mine area, we converted the percent mining area values to z-scores, stratified by the two aridity zones. For watersheds with mining area, the stratified z-scores ranged from -3.00 to 2.78. Watersheds with z-score values greater than zero had low percentages of mining but we didn’t want to treat the presence of any mine area, no matter how small, as a positive attribute. Thus, we rescaled the z-scores for watersheds with mining area. We explored three different rescaling scenarios that varied in penalty strength and chose a -2.0 SD penalty as the most appropriate to reflect the detrimental water quality impacts of coal mining in the Appalachian region of the Eastern U.S. and mineral mining in the Western U.S.

In all scenarios, HUC-12 watersheds with the lowest percentage of mining area received the smallest penalty (-0.1 SD) while the largest penalty went to watersheds with the highest mining area percentage (-2.0 SD, Figure S36). The mining penalty was then deducted from the HUC-12 cumulative SPARROW water quality index z-scores (Figure S37). Although we were unable to find information on the reclamation status of western mines, water quality was ultimately weighted lower in the arid ecoregions than in the humid ecoregions (see Condition Integration) and the role of mining areas should be considered when interpreting water quality values in the Western U.S.

Figure S36. Mining penalty deducted from cumulative water quality score.

This map groups HUC-12 watersheds into classes based on their mining penalty. Watersheds in yellow had no estimated mining area and thus received no mining penalty. Watersheds in dark brown had the largest percentage of mining area relative to all other watersheds with mine land, and received the largest range of mining penalties, up to 2 SD.

Figure S37. Cumulative water quality score with mining penalty.

This map reflects the map shown in Figure S35 with the mining area penalty shown in Figure S36 subtracted from the score.

#### Impervious Surface Override

Impervious surfaces, such as asphalt or concrete, are surfaces incapable of being penetrated by water. These surfaces alter the natural pattern of rainwater soaking into the ground and slowly seeping into streams. Instead, rainwater accumulates and flows rapidly overland, altering streams in important ways (Chithra et al. 2015). Storm drains deliver large volumes of water to streams much faster than would occur naturally, resulting in flooding and bank erosion. Flows peak more rapidly during storms, and peak flows are higher and more frequent. Lack of groundwater recharge during rain events leads to lower daily base flows. Additionally, stream channels become wider, less stable, and less complex. Stream inhabitants are stressed, displaced, or killed by fast moving water and the debris, sediment, and disturbed channel habitat it brings. Pollutants such as gasoline, oil, and fertilizers accumulate on impervious surfaces and are washed into the streams. Finally, during hot weather, rain that falls on impervious surfaces becomes superheated and can stress or kill stream biota.

All indicators of stream quality relative to biotic condition, hydrologic integrity, and water quality decline with increasing watershed imperviousness. Research suggests that aquatic systems become seriously impacted when watershed impervious cover exceeds 10% (CWP 2003) and show significant declines in many stream taxa at levels of impervious surface as low as 0.5 to 2% of the watershed. Serious (40-45%) declines in invertebrates, fish, amphibians have been found at watershed imperviousness greater than 3% (King & Baker 2010).

While we calculated condition metrics for all HUC-12 watersheds, the impact of impervious surfaces in highly urbanized watersheds can override other condition characteristics. In these situations, we applied an impervious override to account for high levels of impervious surfaces. To identify highly urbanized watersheds, we calculated the average percent impervious surface in each HUC-12 using zonal statistics in a GIS (Figure S38). We clipped the HUC-12 watersheds to the CONUS boundary and then calculated the area of each. We evaluated different impervious surface percent thresholds and determined that a threshold greater than 20% identified the most urban watersheds across the country. The small number of HUC-12s that met this threshold (1,487, less than 2%), were automatically assigned a “Far Below Average” condition score (Figure S39).

Figure S38. HUC-12 watersheds shown by average 2019 NLCD Percent Developed Imperviousness in five impact classes.

Figure S39. HUC-12 watersheds with greater than 20% impervious surface.

These watersheds (n=1,487) automatically received an overall condition score of “Far Below Average.”

#### Integration of Condition Metrics

After assigning HUC-12 watersheds that met the impervious surface threshold an overall condition score of “Far Below Average,” we explored different approaches and weights to combine the naturalness metric with the water quality metric. In our final integration, we assigned the naturalness metric the highest weight of five, and the water quality with mining penalty a weight of three for humid ecoregions and a weight of two for arid ecoregions. We weighted the water quality metric lower in the arid ecoregions because water availability is a bigger stressor. The impervious surface override was applied equally in both regions.

*Humid Region Condition = (5*Naturalness + 3*Water quality with Mining penalty) / 8*

*Arid Region Condition = (5*Naturalness + 2*Water quality with Mining penalty) / 7*

Lastly, the condition z-score was capped at one rather than allowing it to go as high as three for the arid intermittent HUC-12s, defined as those with more than 75% of their streams and rivers (size 11 and larger) coded as intermittent and less than one km of perennial large river (size 30 and larger) (Figure S40). This was done to limit the extreme positive influence of condition in these watersheds where there is little permanent water and minimal amounts of freshwater habitats available for freshwater dependent biota.

Figure S40. Final condition score.

This map shows the integrated condition score after accounting for arid HUC-12 watersheds characterized by intermittent streams and rivers.

### Integrated Physical Score

*Physical Score = (1*Connectivity + 1*Condition) / 2*

Our final physical score was an equally weighted combination of the connectivity score (Figure S27) and the condition score (Figure S40).

Figure S41. Physical score.

This map shows the final integration of the connectivity score (Figure S27) and the condition score (Figure S40) with appropriate weights, overrides, and score caps.

## Water Score

*Water Score = Potential Water Availability + Hydrologic Alteration*

The water score was designed to estimate the availability of ample unaltered water to each river network. As the natural levels of precipitation, surface water, and groundwater differ markedly across the U.S., we often had to stratify the results by climate regions (humid, arid, and xeric) to obtain accurate and locally relevant results. The score is a function of potential water availability and hydrologic alteration, which we describe in detail below.

### Potential Water Availability

*Potential Water Availability = Surface Water Index + Groundwater Adjustments*

Potential water availability accounts for the presence of significant surface water and the potential presence of groundwater adjusted for depletion near the surface. We reasoned that areas with greater availability of water will be more resilient to climate change (Wilby 2020), particularly in the arid region of the U.S. where water availability is a key factor in distinguishing resilient systems (Rieman & Isaak 2010).

#### Surface Water Index

Land surfaces that express or store perennial water support freshwater resilience by providing direct habitat for freshwater biota, and by collecting precipitation and providing gradual release and continual access to water throughout the season and during droughts. Surface water is made available throughout the year through a complex set of hydrologic processes integrating precipitation, transpiration, evaporation, overland flow, depressional storage, interflow, and groundwater baseflow (Figure S42).

While we anticipate changes in precipitation, evaporation, and transpiration rates as the climate continues to change, local hydrologic processes driven by geophysical processes like topography and soil type will continue to function. Thus, we developed a surface water storage index based on more stable topographic features such as depressions and gullies, runoff waterways, incised channels; areas with predicted persistent snow; as well as verifiable expressions of surface water such as wetlands and perennial streams and rivers.

Figure S42. Hydrologic processes (Tarboton 2003).

##### **Mapping Surface Water**

To create an index of surface water likely to be meaningful under future climate scenarios, we compiled existing data from multiple sources (Table S7) on the location of:

- Wetlands, springs, perennial lakes, streams, and rivers, and floodplains
- Pluvial depressions and moisture accumulating flats adjusted for land cover type
- Areas of more persistent snowpack in the western U.S.
- Tile drainage applied in heavily agricultural areas

These inputs were merged into a single dataset reflecting the enduring sources and storage areas for surface water across CONUS (Figure S43). As these areas have distinct topographic signatures or are associated with groundwater outlets such as springs or seeps, we expect their locations to remain relatively stable into the future, even though the actual amount of water they collect and store will fluctuate with changes in the climate. We recognized the massive influence of tile drainage in parts of the country where agriculture is dominant and is likely to remain dominant into the future by incorporating a recent dataset showing areas of tile drainage (Valayamkunnath et al. 2020)

Table S7. Data sources for surface water features and modifiers in the surface water index.

| **Feature** | **Data Source** |
| --- | --- |
| Wetlands | NLCD, Dewitz and USGS 2021 |
| Perennial Rivers | NHDPlusV21, USGS 2016 |
| Perennial Headwaters | NHDPlusV21, USGS 2016 |
| Perennial Waterbodies | NHDPlusV21, USGS 2016 |
| Springs and Seeps | NHDPlusV21, USGS 2016 |
| Wet Flat Landforms | TNC 2020a |
| Pluvial Moist Flat Landform | TNC 2020a |
| Tile Drained Agriculture | Valayamkunnath et al. (2020) |
| Persistent Snowpack | Developed for this analysis |

Figure S43. Inputs to the surface water index.

##### **Persistent Snowpack**

In the western U.S., many freshwater ecosystems are dependent on snow as a high percentage of their total water balance (Mayer & Naman 2011; Barnhart et al. 2016). As the climate changes, areas with persistent snowpack are likely to provide some freshwater systems with a more continuous supply of freshwater, increasing their resilience. To identify these areas, we developed a snowpack persistence map based on elevation and topographic influence on local temperature, climate, and solar radiation.

First, we used datasets from TNC (Anderson et al. 2019) and LANDFIRE (2022) to identify upper montane and higher elevation zones as they are expected to maintain colder temperatures into the future, facilitating the persistence of deep snow accumulations and later snowmelt (Jones et al. 2021; Provencher et al. 2021). Upper montane elevation thresholds differed by ecoregion and ranged from 800 meters in the Pacific Northwest to 2,422 m in the Sierra Nevada. Elevation thresholds for the Rocky Mountain and Desert Southwest ecoregions were from Anderson et al. (2019). For other western ecoregions, we defined the lowest elevation as the mean elevation (0.5 SD) of biophysical settings that only occurred in the upper montane zone of each ecoregion (Comer 2022; LANDFIRE 2022). To identify the cells most hydrologically dependent on snowpack, we removed areas where snow comprised less than 50% of water in a NHDPlus catchment water balance model (Wolock & McCabe 2017).

Lastly, we used the Continuous Heat-Insolation Load Index (CHILI, Theobald et al. 2015; Theobald 2022) to assign surface water index scores for the cells identified above. The CHILI model uses topographic slope and latitude to predict potential direct radiation. We considered areas with less incident radiation and a lower heat load, such as north-facing slopes, to be more persistent as they retain snow longer. We z-scored the CHILI data within the upper montane and higher-elevation zones to distinguish areas receiving the least solar radiation (e.g., most persistent snowpack) from those experiencing the most solar radiation (less persistent and/or faster melting snow). We classified the continuous CHILI z-scores into three classes for inclusion in the surface water index which had values that ranged from 0 to 500, with 500 indicating the most water storage and 0 the least. Cells with the least solar radiation, those that scored greater than “Average” in the CHILI z-scores, were assigned a surface water index value of 500. Areas with an “Average” CHILI z-score received a value of 400 while cells receiving the most solar radiation, those with z-scores less than “Average,” were assigned a value of 300 (Figure S44).

Figure S44. Areas of persistent snowpack.

##### Integrated Surface Water Index

To create a continuous surface water index, the compiled inputs were weighted from 0 to 500 based on their expression and storage of surface water and their relative importance to river networks (Table S8). The input weights for certain features were adjusted by climatic zone to reflect the greater importance of perennial water in the arid zone where these features are rare and often the only wet habitats. Wetlands and snowpack areas were given the highest value (500), reflecting their ability to collect and store water, and slowly release it over the season to feed flows in adjacent streams and rivers. Wetlands also directly provide aquatic habitat for many freshwater dependent species throughout the year. Perennial streams, rivers, lakes, and springs/seeps were given moderate values in the humid zone (50-100) where they are relatively common, but higher values in the arid zone (150-300), where they are often the only source of available water and aquatic habitat. Fluvial wet flats and pluvial moist flats are topographic depressions and channels that accumulate overland flows during precipitation events or overbank flooding but are not continuously wet, and thus were given lower values (5-100). In the agricultural regions of the country, the weights on pluvial landforms were decreased slightly where there was tile drainage to reflect the negative effect of tile drainage on water storage.

We then calculated the mean of the surface water index grid cells for each HUC-12 and transformed the values to z-scores relative to CONUS (Figure S45). The results highlight areas where we expect surface water to be more persistent, given enduring topographic water storage and surface expression. This analysis does not consider precipitation patterns and as the humid and arid regions differ markedly in overall precipitation, a less than “Average” score in the humid region may have more absolute surface water than a greater than “Average” score in the arid region.

Table S8. Surface water inputs and relative weighting scheme.

|  | **Land Cover** | | | | | | |
| --- | --- | --- | --- | --- | --- | --- | --- |
|  | **Natural Land** | |  | **Tile Ag.** | **Row** | **Tile Ag. &Row** |  |
| **Feature** | **Arid** | **Humid** | **Hay** | **& Hay** | **Crops** | **Crops** | **Developed** |
| Wetlands | 500 | 500 |  |  |  |  |  |
| Persistent Snowpack | 300-500 |  |  |  |  |  |  |
| Waterbodies: Natural | 300 | 300 |  |  |  |  |  |
| Springs & Seeps | 300 | 100 |  |  |  |  |  |
| Perennial Rivers | 150 | 50 |  |  |  |  |  |
| Perennial Headwaters | 300 | 100 |  |  |  |  |  |
| Waterbodies:  non-natural | 50 | 50 |  |  |  |  |  |
| Wet Flat | 50 | 100 | 50-60 | 30 | 20 | 10 | 0 |
| Pluvial or Moist Flat | 25 | 25 | 20 | 15 | 10 | 5 | 0 |

Figure S45. Surface water index.

This map shows the integrated surface water score.

#### Groundwater Adjustments

Groundwater inputs support freshwater resilience by providing baseflow in times of low precipitation, colder water under moderate temperatures, and maintaining a stable hydrograph. In arid regions, groundwater supply is often critical to maintaining perennial rivers and local water sources such as seeps and springs. In some cases, karst underground freshwater rivers or springs provide important habitat and refuges for freshwater biodiversity (Hare et al. 2021). We accounted for the groundwater influence on available water by developing hydrologic landscapes derived from soils, geology, and aquifer maps, and we also incorporated the location of springs, seeps, and perennial rivers. To adjust for groundwater depletion, we used remotely sensed measures of near-surface groundwater change over the last 18 years.

##### Springs and Seeps Adjustment

As groundwater availability is complex, we wanted to confirm groundwater areas with other sources of data. The NHDPlus high resolution springs/seep point dataset (USGS 2016) provides information on the locations where spring water emerges and thus is available at the surface for freshwater biota. A spring is a water resource formed when a landform (often a hillslope or valley bottom) intersects groundwater at or below the local water table. Springs may be formed in any sort of rock at bedrock fractures, although they are common in limestone and dolomite geology because these geologies fracture relatively easily when weak carbonic acid from percolating rainwater enters and dissolves the bedrock. Factors such as fracture size, aquifer water pressure, size of the spring basin, and rainfall influence the amount of water flowing from a spring. Although the spring dataset did not contain information on the amount of water emerging, we treated high spring density as an indicator of confirmed groundwater availability.

We calculated a spring density map based on the density of spring points in each HUC-12. We converted the results to z-score for those HUC12s containing a spring. We selected all areas that scored greater than average (z-scores > 0) and rescaled them to be between 0 and 1 (Figure S46). The rescaled z-score was used to increase the potential water availability score as an override if the score was higher than the surface water index score.

Figure S46. Spring density increase.

Areas where spring density is greater than average, with scores reflecting relative density per HUC-12.

##### Perennial Streams & Rivers Adjustment

In arid ecoregions, perennial streams and rivers are essential for maintaining freshwater biodiversity and resilience as they represent the only places where water is reliably found throughout the year. For example, 56% of the streamflow in the Upper Colorado River Basin originates as baseflow, with precipitation being the dominant driver of spatial variability in baseflow (Miller et al. 2016). Because arid region rivers are so dependent on groundwater and snowmelt, we used the occurrence of perennial rivers in this region as an indicator of groundwater at or near the surface.

To account for their importance in the arid region, we used an override to increase the potential water availability score of HUC-12s that had perennial rivers and streams. For each qualifying HUC-12, we increased the water score from 0.10 to 1.0 SD based on the percentage of perennial streams in the HUC, ranging from 10% to 100%. Additionally, we increased the water score of a HUC to a minimum of “Above Average” (1 SD) if it had a perennial river greater than 1,000 km^2^ in drainage area, regardless of the HUC perennial river percentage (Figure S47). This increase was only applied if the score was not already greater than 1 SD based on the surface water index and spring density.

Figure S47. Perennial streams and rivers adjustment.

This map shows where the perennial streams and rivers increase from 0.1-1.0 SD was applied.

##### Groundwater Depletion Adjustment

Our final groundwater adjustment to the water availability score was a penalty imposed for groundwater depletion in the arid region, usually due to excessive pumping from underground aquifers. Following the advice of TNC’s global groundwater group, we imposed a larger penalty for groundwater depletion in high groundwater dependent Hydrologic Landscape Regions (HLR). Areas in low groundwater HLRs were given a small penalty as these areas are likely to be more surface water dependent and groundwater depletion has less of an impact.

###### Hydrologic Landscape Regions

Groundwater is notoriously difficult to map at a national scale, as its expression is often subject to recharge levels and underground flow hydrology that is poorly understood. Multiple attempts have been made to interpolate baseflow from gauge data with limited success (Wolock 2003; Santhi 2008; Wenger et al. 2010), but an approach developed by Wolock et al. (2004) for identifying and mapping hydrologic landscape regions (HLRs) proved to be useful for our purposes.

HLRs delineate groundwater potential based on dominant soil, bedrock, and aquifer characteristics. The landscape is divided into categories based on similarities in landforms and geologic texture, and these groups are assigned weights that reflect their potential to support and store groundwater. We created an HLR map for CONUS following Wolock et al. (2004), using their suggested weightings for the individual geophysical components but with more recent soil (TNC 2020c), geology (TNC 2020c), and landform data (TNC 2020a) to map those components (Figure S48).

The methods used to develop of our HLR groundwater potential classes are described later in this Supplemental Index.

Figure S48. Hydrologic landscape regions (HLRs).

This map of groundwater potential is revised from Wolock et al. (2004) using recent data on soil texture, bedrock geology, and landforms.

###### Groundwater Depletion Analysis

We used a new remotely sensed dataset of shallow groundwater depletion, NASA’s GRACE Shallow Groundwater Drought Indicator: Wetness Percentile relative to 1948-2012 (Houborg et al. 2012; Getirana et al. 2020). The GRACE data measures how altered the groundwater volume is compared to historic levels. It integrates multiple ground and space based observing systems, including NASA's Gravity Recovery and Climate Experiment (GRACE) satellite mission and provides weekly data at 12.5-km resolution grid cells across CONUS from 2003-2022. The GRACE satellite pair detects small changes in the Earth's gravity field caused by the redistribution of water on and beneath the land surface. The resulting maps of North America depict the “Groundwater Wetness Percentile,” which compares the amount of groundwater storage at a given date with the historic period of 1948-2012. Negative changes are primarily a result of groundwater withdrawals, while positive changes appear to result from irrigation, but they may also reflect climatic changes in precipitation.

###### Groundwater Depletion Metric

We focused our analysis on the months of February and March, the period of most complete groundwater recharge. We compiled the data for an 18-year period (2003-2021) relative to a 64-yr. average (1948-2012). We categorized the results into classes representing the depletion level most common over the 18-year period. We assigned each grid cell the most frequent percentile class (0-20%, 20-50%, 50-80%, 80-100%) relative to the historic period (Figure S49). For example, a result of 0-20% indicates that in February and March for the years 2003-2021, the shallow groundwater was most frequently at 0-20% of its historic (64-year) level.

The GRACE data focuses on relative change and does not consider the total groundwater storage present or available. After review of the data, we limited our groundwater penalty to the most severely altered class in the arid region, where we were most confident that shallow groundwater declines would directly affect water availability and resilience. We defined this as areas of 0-20% historic groundwater levels in the arid freshwater ecoregions (Figure S50). We weighted the penalty by HLRs, giving the greatest penalty to regions most dependent on groundwater, and the least to regions primarily dependent on surface water. Thus, HUCs in the 0-20% high depletion class received a penalty of -0.25 SD to -0.50 SD depending on the proportion of the HUC covered (10%-100%). HUCs in moderate groundwater HLRs received a penalty of -0.05 to -0.25 SD, and those in the low groundwater HLR class received no penalty.

Figure S49. Shallow groundwater depletion categories.

The map shows the most frequent percentile class (0-20%, 20-50%, 50-80%, 80-100%) found in Feb. and March during an 18-year period. A result of 0-20% indicates that over the period from 2003-2021, the shallow groundwater was most frequently at 0-20% of its historic level.

Figure S50. Areas receiving the shallow groundwater depletion penalty.

#### Integrated Map of Potential Water Availability

Our final potential water availability score was calculated differently for arid and humid regions. In both, we began with the surface water score and applied the HLR-weighted increases and penalties for groundwater springs, seeps, perennial rivers, and shallow groundwater depletion. In the humid region, the final score is dominated by surface water availability, driven by wetlands and topographic storage, with an adjustment for springs and seeps. In the arid region, the score reflects a more combined surface-groundwater score reflecting wetland features, snowpack areas, springs and seeps, perennial rivers, and groundwater depletion (Figure S51):

*Humid Region: PWA = Surface Water Index with Spring Density Override where applicable.*

*Arid Region: PWA = Surface Water Index with Spring Density or Perennial Rivers Override and HLR Weighted Groundwater Depletion penalty applied where applicable*

Figure S51. Potential water availability.

The map is stratified by the arid and humid regions (black line) and includes all the appropriate surface water weights and groundwater adjustments.

### Flow Alteration

Protecting and restoring natural flow regimes, hydrologic integrity, and environmental flows are consistently pointed to as climate change adaptation and biodiversity conservation strategies for inland rivers (Paukert et al. 2021; Wilby 2020; Tickner et al. 2020). Flow is considered the master variable to sustain ecological integrity (Poff et al. 1997). We accounted for flow alteration using the Hydrologic Alteration Index (HAI) from McManamay et al. (2022). This index compares stream flows to reference conditions at a set of gages, then uses random forest models to assign a degree of flow alteration to each NHDPlus stream reach. We summarized the HAI for river reaches (drainage area greater than 100 km^2^) at the HUC-12 scale using a length-weighted average that accounted for whether a reach was intermittent or perennial. For HUC-12s with no river length, we summarized the HAI for stream and headwater reaches based on the proportion of perennial and intermittent length.

HUCs were then z-scored across CONUS to derive a final hydrologic alteration score where 3 SD equaled no alteration, -3 SD equaled highly altered, and 0 was the average degree of alteration. We capped the flow alteration score at 1 for HUCs dominated by intermittent streams in the arid ecoregion (criteria = greater than 75% intermittent and/or less than 25% perennial). We did this because alteration was not independent of the amount of available water, and intermittent systems usually did not have enough surface water to experience flow alteration. This allowed us to distinguish flow alteration from little water (Figure S52).

Figure S52. Flow alteration score.

Hydrologic Alteration Index (HAI) scores from McManamay et al. (2022) were summarized for rivers by HUC-12 watersheds, transformed to z-scores relative to CONUS, and capped for high scores in the intermittent systems of the arid west.

### Integrated Water Score

*Potential Water Availability + Flow Alteration*

For each HUC-12, we integrated potential water availability and flow alteration into a water score by taking a weighted average of the two scores, with the weights differing substantially as the climatic regions became more arid (Figure S53). In the humid region where water is readily available, the score was based primarily on flow alteration (three times the weight). In arid regions dominated by perennial rivers, the score was an equal combination of water availability and alteration. In arid regions characterized by intermittent streams, the score was based primarily on water availability (three times the weight) as water is the more limiting factor in these extremely dry landscapes.

The final water score map (Figure S54) is stratified by these regions, and the scores are relative to each.

- *Humid = (3*Flow Alteration+ 1*Potential Water Availability / 4*
- *Arid-Perennial = (1*Flow Alteration + 1*Potential Water Availability)/ 2*
- *Arid-Intermittent = (1*Flow Alteration + 3*Potential Water Availability / 4*

Figure S53. Stratification regions for the water score.

Figure S54. Water score.

This map shows the final water score after combining potential water availability and hydrologic alteration with the weights and adjustments described in the text. This map is stratified by three climatic regions (humid, arid-perennial, and arid-intermittent) thus the results are relative to those regions.

## Integrated Freshwater Resilience Score

We determined a resilience score for each HUC-12 by calculating a weighted average of the physical score and the water score, with the relative weight of each component dependent on the climatic region (Figure S55, Table S9). In humid regions, more weight was given to the physical score, emphasizing the size and condition of the network. Less weight was given to the water score, which primarily reflects hydrologic alteration. Very little weight was given to water availability as water is generally not a limiting factor in this region. In the arid region, we gave equal weight to the physical and water score, reflecting the relative importance of both these factors. In xeric regions, more weight was given to the water score, with potential water availability being the largest contributor to the score. Arid non-xeric regions had intermediate weights (Table S9).

Table S9. Weights for combining the physical and water scores by climatic region.

| **Climatic Region** | **Physical Score Weight** | **Water Score Weight** |
| --- | --- | --- |
| Humid | 2x | 1x |
| Arid non-xeric | 1x | 1x |
| Arid xeric – more perennial | 1x | 2x |
| Arid xeric – more intermittent | 1x | 4x |

**Very High:** Water Availability

**Moderate**: Hydrologic Alteration

**Low:** Connectivity, Condition

**High:** Water Availability

**Moderate**: Hydrologic Alteration

**Low:** Connectivity, Condition

**Equal:** Connectivity, Condition, Hydrologic Alteration, Water Availability

**High:** Connectivity, Condition

**Moderate**: Hydrologic Alteration

**Low:** Water Availability

Figure S55. Climatic regions and variable influence.

These are the same climatic regions used throughout the analysis. Annotations describe the dominant factors in each of the regions.

### Best in Ecoregion

To ensure representation of all freshwater ecosystems, our final step was to include an ecoregional override. We Z-scored the resilience score relative to within each freshwater ecoregion (WWF & TNC 2019) and terrestrial ecoregion (TNC 2020b) and then selected those HUCS scoring slightly above average or greater (>0.5 SD) in either their freshwater or terrestrial resilience as the potential set of high ecoregional scoring HUCS. If these HUC’s ecoregional score was higher than the CONUS score and the CONUS score was not already greater than “Average” (> 0.5 SD), the HUC’s final resilience score was increased and rescaled to be in the “Slightly Above Average” resilience class (> 0.5 to 1 SD). To prevent increasing the score of vulnerable watersheds even if they were scoring > 0.5 in the ecoregional rescale, HUCs also had to meet the following criteria:

- “Average” for resilience, and
- “Average” or higher for all three major components: size-diversity, condition, water alteration, and
- Greater than “Average” for water availability if in the arid/xeric climatic zone

The ecoregional override raised the score of 3% of the HUCs (2,395 out of 95,973) to “Slightly Above Average,” ensuring that the most resilient HUCs in each ecoregion were (Figure S56). The final freshwater resilience score incorporates this last adjustment and identifies 26% of CONUS as scoring greater than “Average” for resilience (Figure S57, Table S10).

Figure S56. Areas increased to “Slightly Above Average” resilience score based on an ecoregional override.

Figure S57. Final freshwater resilience score.

This integrated map is stratified by arid and humid climatic regions and incorporates all the appropriate scores and adjustments relative to each region.

Table S10. Length and percentage of each resilience category.

|  | **Freshwater Resilience Score** | | | | | | |  |
| --- | --- | --- | --- | --- | --- | --- | --- | --- |
| **Units** | **Far Above Average** | **Above Average** | **Slightly Above Average** | **Average** | **Slightly Below Average** | **Below Average** | **Far Below Average** | **Total** |
| **Kilometers** | 3,559 | 371,438 | 972,020 | 3,049,329 | 596,557 | 146,670 | 6,561 | 5,146,134 |
| **Miles** | 2,211 | 230,801 | 603,985 | 1,894,765 | 370,683 | 91,136 | 4,077 | 3,197,658 |
| **Percent** | 0% | 7% | 19% | 59% | 12% | 3% | 0% | 100% |
| **Cumulative Percent** | 0% | 7% | 26% | 85% | 97% | 100% | 100% | 100% |

# 1.Macrohabitat Components

For the connectivity component of the physical score, we calculated a diversity score that measures the physical habitat variation in a Functionally Connected Network (FCN) and was calculated as follows:

*Diversity Score = (Temperature score + Macrohabitat Diversity score) / 2*

Macrohabitats are the finest scale unit of hierarchical aquatic classification types defined in TNC’s freshwater classification approach (Higgins et al. 2005). They define stream reach types or lake types and are based on key abiotic variables known to structure aquatic communities at the reach or lake scale. For this analysis, we created simplified macrohabitat types to represent variation in major aquatic physical habitats at the stream reach or individual lake scale using variables possible to model in GIS. Our hypothesis was that HUC-12 units with more macrohabitats would provide more options for species to adapt to change and thus support resilience. Here, we provide details on how we created the simplified macrohabitat types.

## Size

Stream size is indicative of major ecosystem changes along the stream-river continuum, such as transitions in the state and source of energy and ecosystem metabolism. Stream size has been given the highest classification importance in many reach scale stream classification systems because of its strong effect on determining aquatic biological assemblages at the reach scale (Vannote et al.1980; Higgins et al. 2005). The well-known "river continuum concept," depicted in Figure S58, illustrates how the physical size of a stream relates to major ecosystem changes from small headwater streams dominated by coarse organic matter to autochthonous production by plankton in large river mouths (Vannote et al. 1980; FISRWG 1998).

Figure S58. The River Continuum Concept from Vannote et al. 1980 (FISRWG 1998).

Size classes were defined based on total upstream drainage which is the most stable and geomorphologically comparable measure of size across the country. Size class breaks were based on the National River Fish Habitat Condition Assessment (Wang et al. 2011) which was designed to provide a coarse-level understanding of diversity at a national scale. We made two small modifications, adding two very large river classes and combining the two smallest classes (Table S11).

Table S11. River reach size classes.

| **Size Class Number** | **Size Class Name** | **Catchment Area (km^2^)** |
| --- | --- | --- |
| 10,11 | Headwaters, Creek | ≤ 100 |
| 20 | Small Rivers | ≤ 1000 |
| 30 | Medium Rivers | ≤ 10,000 |
| 40 | Mainstem Rivers | ≤ 25,000 |
| 50 | Large Rivers | ≤ 100,000 |
| 60 | Great Rivers | ≤ 1,000,000 |
| 70 | Mega Rivers | > 1,000,000 |

## Gradient

Stream gradient also highly influences aquatic communities at the reach scale due to its influence on stream bed morphology, flow velocity, sediment transport/deposition, substrate, and grain size (Rosgen 1994). As shown in Figure S59, high gradient streams are dominated by step-pools to plane-bed systems with substrates of cobble and boulders, and colluvial sediment transport. Low gradient systems are dominated by riffle-pool systems, with substrates of sand, gravel, and cobble, alluvial storage, and depositional sediment regimes. Very low gradient streams are dominated by ripple-dune streams with very high sinuosity. These rivers have sand, gravel and finer sediment substrates, alluvial storage and depositional sediment regimes, and slight entrenchment with critical adjacent floodplain systems (Rosgen 1996; Allan 1995).

Figure S59. Illustration of how gradient shapes riverine habitats (FISRWG 1998).

Gradient classes were based on McManamay et al. (2019), except that we combined the two highest classes, which were inextricably correlated (Table S12). Gradient in the source NHDPlus reaches represents the average gradient over the length of an entire arc based on the length and elevational change from node to node.

Table S12. Gradient classes.

| **Gradient Class Name** |  | **Slope** |
| --- | --- | --- |
| Very Low |  | <0.001 |
| Low |  | 0.001-0.005 |
| Moderate |  | 0.005-0.02 |
| Moderate - High |  | 0.02-0.04 |
| High and Steep |  | >0.04 |

## Local Temperature

Stream temperature is fundamental to freshwater resilience because it sets the physiological limits of persistence for many organisms and temperature extremes may directly preclude certain taxa from inhabiting a water body. Seasonal changes in water temperature often cue development or migration, and temperature can influence growth rates and fecundity. Ideally, a resilient stream network would span a range of current temperatures offering options for both coldwater and warmwater species and provide connected habitats for species to stay within their thermal preferences in the future (Woodward et al. 2010).

Mean summer stream water temperature modeled data was available for most reaches from the EPA Stream-Catchment (StreamCat) dataset (Hill et al. 2016), a national source widely used to study reference temperatures. We extracted the variable Mean Summer Stream Temperature (MSST) to represent the predicted reference-condition mean summer stream temperatures. The missing data for streams in Northern Michigan was filled in using Lyons et al. (2009). Temperature predictions were not available for all very large rivers because the source EPA MSST data constrained their predictions to stream segments that were within the range of predictor values used to develop the model and the largest rivers (>100,000 km^2^) were not represented in the gage dataset.

The continuous predictions were placed into five summer temperature classes following McManamay et al. (2019), except that we lumped the two coldest classes for which there were few reaches (Table S13). The temperature class breaks closely matched other stream and river classifications developed in Michigan (Lyons et al. 2009), the Appalachians (Olivero-Sheldon et al. 2015), and Eastern U.S (McManamay et al. 2018).

Table S13. Temperature classes in Celsius and Fahrenheit.

| **Temperature Class** | **Celsius** | **Fahrenheit** |
| --- | --- | --- |
| Very Cold | <15 | <59 |
| Cold | >15-18 | 59-64.4 |
| Cool | >18-21 | 64.4-69.8 |
| Cool-Warm | >21-24 | 69.8-75.2 |
| Warm | >24C | >75/.2 |

## Tidal

Streams and rivers that connect directly to the ocean or to large tidal river estuaries are influenced by ocean tides. Their water flow and level fluctuate with the tides and salinity can range from fresh to brackish to saline. They often represent a unique diversity of habitat for anadromous, diadromous, and euryhaline species. We had two classes, tidal and non-tidal, as defined by the “Tidal” attribute in the source NHDPlus data (USGS 2016).

## Lakes

We included lakes in the HUC-12 macrohabitat diversity to represent distinct lentic ecosystem types. Depth is a critical variable related to summer lake stratification and the presence of permanent coldwater habitat for species like lake trout, rainbow smelt, burbot, and landlocked salmon. For the macrohabitat analysis, we defined lakes as any waterbody over ten acres in surface area and we classified them into two types: 1) deep, > 30 ft., and 2) shallow, < 30 ft. We assumed that waterbodies without depth records were shallow.

A summary of the five components and their classes is provided in Table S14 and Figure S60.

Table S14. Macrohabitat component class summary.

| **Size Class (cumulative drainage area in km^2^)** | **Gradient** | **Temperature** | **Tidal** | **Lakes** |
| --- | --- | --- | --- | --- |
| Headwaters-Creek (≤ 100) | Very low (<0.001) | Very Cold (<15C; < 59F) | Tidal | Deep |
| Small River (>100 & ≤ 1,000) | Low (0.001 – 0.005) | Cold (>15-18C; 59-64.4F) | Non-tidal | Shallow |
| Medium Rivers (>1,000 & ≤ 10,000) | Moderate (0.005 – 0.02) | Cool (>18-21C; 64.4-69.8F) |  |  |
| Mainstem Rivers (>10,000 & ≤ 25,000) | Moderate-High (0.02 – 0.04) | Cool-Warm (>21-24C; 69.8-75.2F) |  |  |
| Large Rivers (> 25,000 & ≤ 100,000) | High & Steep (>0.04) | Warm (>24C; >75.2) |  |  |
| Great Rivers (> 100,000 & ≤ 1,000,000) |  |  |  |  |
| Mega Rivers (> 1,000,000) |  |  |  |  |

Figure S60. Macrohabitat components.

Clockwise from top: size classes, gradient classes, temperature classes, tidal classes, and lake depth classes.

## Calculating Macrohabitat Diversity

The macrohabitat components were combined at the reach flowline scale to code each NHDPlus flowline with its size class, temperature class, gradient class, and tidal class. Each unique combination of these classes was considered a “macrohabitat.” All macrohabitat types longer than one km in a HUC-12 were extracted and counted toward a total diversity count of types present in the HUC. The total length (km) of all perennial and intermittent/ephemeral stream-river macrohabitat types was summarized. Additionally, the length (km) of only perennial stream-river macrohabitat types was summarized for each HUC-12 to explore the difference in diversity when ephemeral and intermittent streams were included versus when they were excluded. The presence of a deep lake or shallow lake was then added to the total stream-river macrohabitat counts to increase the total by one or two if deep, shallow, or both types of lakes were present in a HUC-12. The final macrohabitat diversity score for each HUC-12 unit was calculated based on the number of macrohabitat types relative to the average number of macrohabitats expected for the size of the HUC. Because the count of macrohabitats was correlated with the size of the HUC, we used a Poisson regression to establish a relationship between the size of the HUC and the count of macrohabitats and then calculated the standardized residuals to identify HUCs that had more or less macrohabitat diversity than expected for their size. The standardized residuals were used as the z-score. See details of the statistical models below.

Perennial Reaches Only Model

m1 <- vglm(formula = CLPm19S755e ~ logSqKM , family = poissonff(), data = tab)

summary(m1)

Call:

vglm(formula = CLPm19S755e ~ logSqKM, family = poissonff(), data = tab)

Coefficients:

Estimate Std. Error z value	Pr(>|z|)

(Intercept) -0.225599	0.021434 -10.53 <0.0000000000000002 ***

logSqKM	0.287002	0.004723	60.77 <0.0000000000000002 ***

Signif. codes: 0 ‘***’ 0.001 ‘**’ 0.01 ‘*’ 0.05 ‘.’ 0.1 ‘ ’ 1

Name of linear predictor: loglink(lambda)

Residual deviance: 168330.6 on 82670 degrees of freedom

Log-likelihood: -185421 on 82670 degrees of freedom

Number of Fisher scoring iterations: 5

No Hauck-Donner effect found in any of the estimates

All Reaches Model, Perennial + Intermittent/Ephemeral (Using R Statistical Package)

m2 <- vglm(formula = CLAm19S755e ~ logSqKM , family = poissonff(), data = tab)

summary(m2)

Call:

vglm(formula = CLAm19S755e ~ logSqKM, family = poissonff(), data = tab)

Coefficients:

Estimate Std. Error z value	Pr(>|z|)

(Intercept) -0.191308	0.017137 -11.16 <0.0000000000000002 ***

logSqKM	0.376811	0.003762 100.17 <0.0000000000000002 ***

Signif. codes: 0 ‘***’ 0.001 ‘**’ 0.01 ‘*’ 0.05 ‘.’ 0.1 ‘ ’ 1

Name of linear predictor: loglink(lambda)

Residual deviance: 73712.31 on 82670 degrees of freedom

Log-likelihood: -170717.5 on 82670 degrees of freedom

Number of Fisher scoring iterations: 5

No Hauck-Donner effect found in any of the estimates

Maps of the residual scores (Figure S61) of the two models highlighted mountainous HUC-12s with higher diversity than expected by area alone. When non-perennial reaches were excluded, the arid west had a significant reduction in diversity scores over much of the arid region. We combined the two scores together giving equal weight to each in the final diversity score, and thus more weight was given to perennial streams which were included in both models.

*Macrohabitat Diversity = (standard residual all streams + standard residual perennial only)/2*

Small dams on headwaters and creeks make the accessibility of all types of macrohabitats within a HUC-12 more difficult. In HUCs with “Average” or worse headwater fragmentation, we adjusted the macrohabitat diversity score by taking the average of the combined macrohabitat diversity z-score and the headwater fragmentation z-score. If headwater fragmentation was low, we kept the diversity score as it was, assuming that some portion of the macrohabitat types present in the HUC would probably be accessible, at least at some part of the year. Finally, we integrated all the components and penalties into a single weighted map of macrohabitat diversity for CONUS (Figure S25).

Figure S61. Weighting factors for macrohabitat diversity score.

Clockwise from upper left: residual z-score for all streams, residual z-score for perennial streams only, and habitat fragmentation penalty.

# 2.Mapping Hydrologic Landscape Region Groundwater Potential

Our goal was to assign each HUC-12 watershed in CONUS to a groundwater potential class based on the hydrologic flow paths corresponding to the soil and geologic setting type as identified in the USGS hydrologic landscape regions (HLRs, Wolock et al. 2004). According to the hypotheses of the HLRs framework, areas with permeable bedrock would have “deep groundwater hydrologic flow paths” and areas with permeable soils would have “shallow groundwater flow paths,” while those with impermeable bedrock and impermeable soils would have overland hydrologic flow paths not driven by groundwater.

The original HLRs dataset used surface sandy soil texture and bedrock and deep aquifer types to estimate the effect of underlying geologic settings on surface runoff, infiltration, and groundwater flow. The underlying geologic setting was summarized for small watersheds (~200 km^2^) which had been generated form a coarse 1-km resolution digital elevation model (DEM) in this original 2004 HLR dataset. The input geology in the original HLRs was based on a very coarse aquifer dataset mapped at a scale of 1:2.5M (USGS 2003). Given the availability of newer and higher resolution bedrock geology and soils datasets, we used the methods in Wolock et al. (2004) to create updated HLRs subsurface and surface permeability datasets.

## Subsurface Permeability

We adopted the permeability classes of general lithologic groups as described in the original HLRs. These classes ranged from the highest permeability rank of seven to the lowest permeability rank of one as follows:

Subsurface Permeability Rank: (1= low permeability, 7 = high permeability)

1: Not a principal aquifer

2: Sandstone aquifers

3: Semiconsolidated sand aquifers

4: Igneous basalt and metamorphic volcanic rock aquifers

5: Sandstone and carbonate rock aquifers

6: Unconsolidated sand and gravel aquifers

7: Carbonate rock aquifers

To assign a subsurface permeability class to each HUC-12 watershed, we initially evaluated the subsurface geologic source used by Wolock et al. (2004) and the USGS map of Principal Aquifers of the 48 Conterminous United States 1:2.5M scale (USGS 2003). The bedrock aquifer types in the USGS (2003) product had been much more finely mapped in recent USGS state bedrock geology maps (Horton 2017) and in other sources such as the USGS National Karst map (Weary & Doctor 2014). Previously, we had used the USGS state geology polygons to develop a terrestrial settings dataset (TNC 2020c) and already classified the formations into a very similar grouping as that used in the HLRs subsurface permeability classes. The recent USGS karst map (Doctor et al. 2020) also provided new finely mapped karst formations. Using these updated geologic sources, we extracted four of the six HLRs subsurface permeability classes from our terrestrial settings (TNC 2020c), the USGS state bedrock data (Horton 2017), and/or the recent karst mapping (Doctor et al. 2022). The updated geologic data were placed into the following HLRs subsurface permeability rank classes (Figure S62):

- Class 2: Sandstone (and non-calcareous shale) aquifers: (sandstones, arenite, arkose, conglomerate, or sandstone mixed with mudstone, claystone, bentonite, politic schist, argillite, phyllite (slightly metamorphosed shale), black shale or oil shale)
- Class 4: Igneous basalt and metamorphic volcanic rock aquifers. Includes basalt, andesite, mafic volcanic, alkali volcanic rock. Mafic and felsic volcanic such as rhyolite, trachyte, basanite in major or minor rock types, USGS Karst dataset: Volcanic rocks with potential for pseudokarst feature. Also mafic/intermediate rock such as: quartz-poor alkaline to slightly acidic igneous and meta-igneous rock. Syenite, diorite, gabbro, anorthosite, migmatite, syenite, tectonite, hornfels, amphibolite, monzonite , amphibole schist, dioritic, and monzodiorite. Ultramafic rocks that were extremely rare in this region, such as magnesium-rich alkaline igneous and meta-igneous rock, serpentine, and peridotites.
- Class 5: Sandstone and carbonate rock aquifers. Mixed sedimentary/limestone formations. Moderately alkaline sedimentary or meta-sedimentary rock with some calcium but less than rocks in the purely calcareous rock aquifer class. Calcareous shales, mixed sedimentary rocks with calcareous components, calcium-silicate, and calcium-silicate schist rocks, usually in the minor components of the rock. Note, in the data set any mixed bedrock with one of the three major components in the dataset listed as limestone or dolostone was assigned here.
- Class 7: Carbonate rock aquifers. Alkaline sedimentary or metasedimentary rock with high calcium content such as limestone, dolostone, marlstone, calcarenite, carbonite, chalk, coquina, marble, and marl; USGS Karst “carbonate rocks at or near the surface.”

As we were unable to extract the remaining two types of deep, non-bedrock aquifers from existing state bedrock geology sources, we used the following two types from the original map of Principal Aquifers of the United States (USGS 2003):

- Class 3: Semiconsolidated sand aquifers
- Class 6: Unconsolidated sand and gravel aquifers

Areas not covered by the above classes were divided into two final classes:

- Class 1: Not a Principal Aquifer, Granite. Included quartz-rich: resistant igneous and meta-igneous or meta-sedimentary rock such as granite, granodiorite, gneiss, tonalite, migmatite, quartzite, mylonite, latite, dacite. Mapped using TNC terrestrial settings (TNC 2020c) and the USGS state bedrock data (Horton 2017).
- Class 1.75: Not a Principal Aquifer, Deep Surficial Sediment Setting. Areas of deep surficial soil where bedrock was deeply buried, and the areas were not identified in the source bedrock or deep semi or unconsolidated sand/gravel aquifer datasets.

Figure S62. Subsurface permeability: bedrock and deep surficial aquifer type

Similar to Wolock et al. (2004), we used small watersheds to summarize the underlying permeability patterns. Each input geologic class was assigned a permeability rank from one to seven. We then calculated the mean subsurface permeability value for each HUC-12 to place the watersheds in one of three summary groups representing high, moderate, and low subsurface permeability as follows (Figure S63):

- Low: 0-1.5
- Moderate >1.5 – 3.25
- High >3.25

Figure S63. Subsurface permeability summary classes.

## Surface Permeability: Deep Sandy Soils

Surface permeability classes were mapped using the approach in Wolock et al. (2004), which focused on identifying areas of surficial sand. The percentage of sand in the soil is directly related to its permeability, with permeability increasing as the percentage of sand in the soil increases (Wolock et al. 2004). Sandy areas were mapped using TNC’s terrestrial settings geology and soils dataset (TNC 2020c) which placed areas of deeper surficial deposits into soil texture categories. In TNC’s terrestrial settings, soil texture was mapped where deeper soils occurred within ecoregions, while bedrock types were mapped elsewhere (and had been used in the previous HLRs subsurface permeability classification). In most ecoregions, the category “discontinuous or patchy surficial material” from the national USGS Surficial Materials in the Conterminous United States (Soller 2009) defined the extent of where bedrock was mapped, and soil texture was mapped in all the remaining areas where deeper soils occurred and bedrock was completely buried. The soil texture in TNC’s terrestrial settings was derived from sources such as SSURGO, STATSGO, POLARIS, and Soller et al. (2012) which represented the best available and appropriate data source in each ecoregion (TNC 2020c).

The categories of sand, acidic loam, and calcareous loam were extracted from TNC’s terrestrial settings geology and soils layer and used to map two categories, loam and sand (Figure S64). In a small portion of the Pacific NW and California where soil texture categories had not been used in the TNC settings, STATSGO2 was used to map sand and sandy loam/loamy sand in areas where deeper soils occurred and were not classified as “discontinuous or patchy bedrock” in Soller (2009).

Figure S64. Deep surficial sandy sediment texture type.

We assigned sand areas a permeability of 200 and loam areas a permeability of 100, half the permeability of sand. We sampled the mean surface permeability value for each HUC-12 and placed the watersheds into three summary groups representing high, moderate, and low surface permeability as follows (Figure S65):

- Low: 0-50
- Moderate: >50-100
- High: >200

Figure S65. Surface permeability summary classes.

## Deriving Groundwater Potential Classes

We combined the summary surface and subsurface bedrock permeability classes to identify areas with low, moderate, or high overall potential groundwater based on enduring geologic and soil settings (Table S15). If an area was high in “either” deep groundwater or shallow groundwater, it was identified as having high overall groundwater availability. If an area was moderate in both, or moderate in either deep groundwater or shallow groundwater, it was placed into the moderate groundwater class. If an area was low in both deep groundwater and shallow groundwater, it received a low overall groundwater potential assignment.

Table S15. Resultant groundwater potential class from the combination of surface and subsurface permeability class.

| DOWN: Subsurface Permeability, Deep Groundwater | ACROSS: Surface Permeability, Shallow Groundwater | | |
| --- | --- | --- | --- |
|  | Low | Moderate | High |
| Low | Low | Moderate | High |
| Moderate | Moderate | Moderate | High |
| High | High | High | High |

Lastly, we augmented the groundwater potential assignment after review of the NHDPlus springs dataset (USGS 2016). We noticed some areas where springs were present and expressing groundwater at the surface, but that had been misclassified as low groundwater areas by the coarse geologic mapping of subsurface and surface material permeability. Although many of these springs reflected key groundwater expressions at the surface, we did not want to automatically increase the HLRs groundwater potential class of all HUC-12 watersheds that contained any spring/seep point because a single spring point could be an error and/or very rare small insignificant groundwater feature. We also did not want to increase the groundwater potential HLRs class of any HUC-12 that was in an area of high snowpack as those spring/seeps are often more surface water driven as snowmelt collects above impermeable bedrock in very shallow mountain soils, which didn’t match the other deeper permeable soil and deep bedrock permeability concepts currently the driver of moderate or high HLRs groundwater potential. Consequently, we decided to move a HUC-12 to a higher groundwater potential class if most of it was not located in areas with 25% or more snow water balance, using the following rules:

- Move HUC-12s with six or more springs to the high groundwater potential class, if not already in the high category (six represents the 1^st^ natural break of a seven-class break distribution on all HUC-12s with a spring)
- Move HUC-12 watersheds with two to five springs and less than 25% snow that are in the low class to the moderate class, or if in the moderate class, move to the high class.

The final groundwater potential classes are shown in Figure S43.

# Estimated Mining Areas

For the water quality component of the condition score, we wanted to include a measure for mine lands in HUC-12 watersheds. While there are a range of both national and regional spatial datasets that identify mine footprints in the U.S., many are outdated, inconsistent, and/or have incomplete geographic coverage. To estimate the potential impact of surface mining activities (e.g., mountaintop removal, quarries, strip mines, open pit mines, dredging) on watersheds, we developed a new dataset of mining footprints in CONUS using current land cover with global, national, regional, and state mining datasets. Each data source is described and then we summarize our approach to identify surface mining areas.

Mining Point Locations

- *U.S. Active Mines and Mineral Processing Plants* (USGS 2005). This is a USGS point dataset of active mines and mineral processing plants in the U.S that we downloaded from <https://mrdata.usgs.gov/mineplant/>. The dataset includes mineral and metal operations considered active in 2003. For our analysis, we removed peat mines and processing plant locations, unless they included the site mines.
- *U.S. Coal Mines* (US EIA 2020). This is a U.S. Energy Information Administration (EIA) dataset. We downloaded a point dataset representing operating surface and underground coal mines in the U.S. in 2020 from the EIA U.S. Energy Atlas at <https://atlas.eia.gov/maps/coal-mines-1>.

Spatial Extents

- *Appalachian Mining Disturbed Areas.* TNC staff developed this layer, which reflects landscape conditions up to and around 2015, by merging several datasets for the Appalachian mining region encompassing the states of Pennsylvania, West Virginia, Ohio, Virginia, Kentucky, Tennessee, and Alabama. Mining disturbance areas were initially compiled using a combination of state mining agency permit data and aerial photo interpretation. Three additional datasets based on regional aerial photo assessment projects were used to improve the accuracy of the mining disturbance areas (Townsend et al. 2009; and Pericak et al. 2018). There were no distinctions made between surface disturbances due to surface mines, valley fills, mining roads, preparation plants, underground mines, or reclaimed mined lands. In Eastern Kentucky, mining areas were based on an aerial photo digitization project completed by TNC in 2006 and updated with data from Pericak et al. (2018) and a Downstream Strategies mining analysis. Western Kentucky mining areas are based solely on agency permit data.
- *Florida Phosphate Mining Areas* (FDEP 2021). Central Florida (Polk, Hillsborough, Manatee, and Hardee counties) has an extensive phosphate mining region that encompasses about 1.3 million acres of land often referred to as the “Bone Valley.” There is an additional mine in Hamilton county located in North Florida. Since 1975, land mined for phosphate in Florida is required to be reclaimed through contouring and revegetation. Florida’s Department of Environmental Protection maintains spatial data on phosphate mine boundaries and reclamation units. We downloaded the 2019 Mandatory Phosphate Reclamation Units that identifies reclamation units of all active mandatory phosphate mines in Florida in 2019 at <https://geodata.dep.state.fl.us/explore?query=phosphate>.
- *Global Mining Areas Dataset* (Maus et al. 2020)*.* Recently published and publicly accessible global dataset of above-ground mining areas derived from analysis of satellite imagery (<https://doi.org/10.1594/PANGAEA.910894>). The dataset includes open cuts, tailings dams, waste rock dumps, water ponds, and processing infrastructure. Note that after completion of the freshwater resilience analysis in June 2023, Maus et al. (2022) published an updated version of the global mining dataset in July 2023 that significantly increased the initial 2020 mining area.
- *LANDFIRE EVT Quarries-Strip Mines-Gravel Pits-Well and Wind Pads* (LANDFIRE 2020). Grid value 7295 from the 2020 LANDFIRE (LF) 2.2.0 30-m Existing Vegetation Type (EVT) raster. We aggregated the selected pixels using the eight nearest neighbors, and then converted to polygon.
- *LCMAP Barren Lands* (Pengra et al. 2021). USGS Land Change Monitoring, Assessment, and Projection (LCMAP) v1.2 30-m raster grids for 1990, 2000, 2010, and 2020 (<https://www.usgs.gov/special-topics/lcmap/collection-12-conus-science-products>). Using the four decadal time periods, we identified cells that transitioned to barren land cover (grid value 8). We aggregated the resultant LCMAP barren pixels using an 8-neighbor region group rule in ArcGIS and converted the grouped cells into a polygon. In examining the LCMAP barren areas, we found they did a good job of expanding existing surface mine areas from other datasets (e.g., USGS surface mines, global mining dataset) but also identified areas where it was difficult to determine if mining was occurring.
- *USGS Surface Mining Areas* (Soulard et al. 2016). USGS produced 30-m raster of moderate to large surface mining areas in CONUS using a semi-automated procedure with mine point locations from <https://onlinelibrary.wiley.com/action/downloadSupplement?doi=10.1002%2Fldr.2412&file=ldr2412-sup-0001-mines.zip> and <https://onlinelibrary.wiley.com/action/downloadSupplement?doi=10.1002%2Fldr.2412&file=ldr2412-sup-0002-gains.zip>).

*Identifying and Refining Surface Mining Areas*

After exploring the above data sources, we used several different approaches, trial and error, and substantial manual review to refine and expand existing mining areas as well as identify new ones.

To refine the spatial extent of mapped mining areas using current land cover, we created a layer of land uses that could be associated with mining operations. We extracted the following 2019 NLCD land cover classes: open water (class 11), development (classes 21-24), barrens (31), shrub/scrub (52), grassland (71), pasture/hay (81), and row crops (82). We removed roads using the 2019 NLCD Impervious Surface source grid, aggregated the remaining cells using a four-neighbor rule, and converted the aggregated raster cells to polygons.

To update and check the USGS surface mine areas, we first merged the USGS 2001 and 2006 surface mine grids, selected mine cells (grid value 1), aggregated the mining pixels using four nearest neighbors, and converted to polygon. We reviewed the resultant polygons with coal and mineral mine point locations and discovered that many of the small sites were not near coal or other mines and appeared to simply be barren areas. We used several rules to address this issue with search distances and the large size threshold from Soulard et al. (2016). First, we retained surface mine polygons that intersected mining polygons from the other mining areas datasets or that were within 500 meters of USGS mineral mine points (excluding processing plants) or within 2,000 meters of coal mine points. We manually reviewed polygons that were greater than 500 m and less than or equal to 1,000 m from USGS mine locations. We grouped the remaining polygons (>250K) into size classes. Polygons less than 10,000 m^2^ did not appear to be surface mines and were removed. Most polygons greater than 10,000 m^2^ and less than 50,000 m^2^ were not mines with a few exceptions that we retained. We manually reviewed all large polygons greater than 100,000 m^2^ and those between 50,000 and 100,000 m^2^. The size thresholds intentionally removed western US oil fields. We retained smaller polygons that intersected larger surface mining polygons that we positively identified as mines. Because the USGS surface mining areas were based on 2006 data, we selected LCMAP barren polygons that intersected the USGS surface mining polygons and found the barren polygons did a great job of capturing new mining areas and helped fill in holes. We converted the selected LCMAP polygons to a raster and merged those with the USGS surface mining grid using Cell Statistics in ArcGIS to retain the data source. Lastly, we intersected the reviewed surface mines with the global mining areas, Appalachian mining footprint, LCMAP barrens, and LANDFIRE EVT mine class to add a field identifying polygons that overlapped with the USGS dataset.

To refine and check the footprints in the 2020 global mines dataset, we clipped the 2019 NLCD land cover mine layer and the LCMAP barrens by the global mining polygons, merged the two and assigned unique values to retain the land cover source. We identified one polygon from the global mining dataset that didn’t intersect these two datasets or the USGS surface mining dataset. The polygon was in Arkansas and review suggested it was not a mine. In the Appalachian region, we manually investigated the global mining footprints that did not intersect TNC’s Appalachian mining footprint and retained those that appeared be mining areas based on imagery and mine point data.

To identify potential new coal mining areas not in the other datasets, we selected LANDFIRE polygons greater than or equal to 250,000 m^2^ that were within 2,000 m of coal mines. After removing the resultant polygons that intersected global mines, USGS surface mines, and Appalachian mines, no new coal areas were identified. We repeated this process for the LCMAP barrens polygons and identified four new potential areas. After examination, one area was retained and all nearby LCMAP barren polygons were selected to represent the full mining footprint based on satellite imagery.

To identify potential new non-coal mining areas, we selected LANDFIRE polygons 100,000 m^2^ or larger within 500 m of mines. A total of 104 polygons remained after removing those that intersected existing mining areas in the other datasets. These were manually reviewed using imagery and the LCMAP barren polygons. Most were retained and the footprint of many was refined by incorporating intersecting LCMAP barren polygons. While manually reviewing the polygons, we identified additional smaller LANDFIRE polygons that improved the mine footprint. We used the same process for the LCMAP barren polygons. There were more than 200 polygons that did not spatially intersect mining polygons in the other datasets, and most were retained after manual review. In some cases, the footprint was improved by incorporating nearby LCMAP polygons or LANDFIRE polygons. After identifying the LANDFIRE and LCMAP surface mines, we ran a spatial intersection between reviewed surface mines and the LCMAP and LANDFIRE smaller polygons to further fill out mining areas where there were intersections. Lastly, we examined mining points that did not have nearby mining area polygons and examined those with the LCMAP barrens, NLCD layer, and the LANDFIRE EVT polygons to identify potential new mining areas.

In addition, there were geographies that required detailed manual review and additional datasets; of note are portions of Florida and Louisiana. While the existing mining datasets identified some of the phosphate mining areas in Central Florida, we wanted to remove reclaimed sites and identify new ones to ensure that only active mines were represented. Using the Florida phosphate mining data, we excluded the reclamation units where work had been completed or there was no disturbance, and selected the units with a future work, work scheduled, or work in progress status. We clipped the global mining, LCMAP barrens, LANDFIRE, and USGS surface mine polygons by the selected reclamation units and retained those polygons to represent the active phosphate mining areas. In Louisiana, there is extensive sand and gravel mining along the Amite River and Bogue Chitto River. The USGS surface mine and other datasets picked up some of these locations, but we ended up manually adding new ones based on imagery examination and using the LCMAP barrens, LANDFIRE, and NLCD datasets.

Finally, we did some additional clean up, error checking, and data source coding for all the selected mining areas. We converted the selected mining polygons to a 30-m raster and merged all the grids using Cell Statistics in ArcGIS to retain the source datasets for all mapped mining areas. We created a mining area raster where all mining areas were coded as 10 and then we merged this on top of the 2019 NLCD land cover for use in the water quality mining metric and the floodplain, riparian, and watershed land cover naturalness metrics.

Freshwater Conservation Status

## General Model for Conservation Status of Freshwater Networks

Conservation of the environment may require both protection, which is the prevention of future degradation (Higgins et al. 2021), and restoration of ecologic function when a system is below a level of viability (Possingham et al. 2015). Ideally then, to say that an instance of an ecosystem is conserved, it would be above a threshold for ecologic condition based on sufficient function and long term viability, and it would be protected, based on sufficient legal mechanisms or community management agreements that prevent further degradation (Higgins et al. 2021). It is very difficult to establish these thresholds. Generally in practice, conservationists prefer to set goals for improving specific dimensions of ecosystems, and even then tend to measure actions taken rather than ecological outcomes, which are difficult to detect and can take many years (Stephenson 2019). Yet 30x30 goals for protecting land, oceans, and inland waters, require that we have a means to say whether something is conserved or not. Here, we have considered how we might measure the conservation status of freshwater river networks in the context of 30x30.

Using our general definition of “conserved,” we have established two axes to assess conservation status (Figure S66). The first is the level of freshwater resilience of a given river reach (Anderson et al. 2024). Freshwater resilience, defined as the ability of a freshwater ecosystem to support biodiversity in the face of a changing climate, encompasses both condition and viability by evaluating the key ecological attributes of rivers from the perspective of what will contribute to the climate resilience of that river into the future. Freshwater resilience is described in Chapter 3 of this report. The second axis is the level of protection, which we determined by combining the American Rivers Protected Rivers Index (CSP 2025) with our own analysis of regulating function protection for hydrologic regime and water quality.

Figure S66. Conservation status is defined by two axes, resilience and protection.

## Protection Class

We define protection as the establishment and implementation of mechanisms that avoid future impacts to freshwater ecosystem key ecological attributes for 25 years or more, or are of high probability of renewal and enforceability, or have completely alleviated the potential for a future threat (Higgins et al. 2021). These key ecological attributes include habitat, water quality, hydrologic regime, connectivity, and biotic composition (Higgins et al. 2021).

The United States has a web of federal laws such as the Clean Water Act that are intended to protect the key ecological attributes of rivers everywhere. One might suppose that rivers everywhere are protected thanks to this law, but that is not the case given the law’s attempt to balance interests, variable implementation by states, and the impact of judicial interpretation of the law. For example, in May 2025, the Supreme Court’s Sackett decision removed almost fifty years of federal protection for wetlands unless they meet a narrow, and not scientifically supported definition in which only wetlands with a surface connection are important to the health of the nation’s waterbodies (McElfish 2023).

Protection mechanisms exist at all levels of governance (**Error! Reference source not found.**) (Flitcroft et al. 2023) , but there are limits to our ability to translate policy to specific locations. As with the freshwater resilience score, our findings are a starting place to begin to identify where and what types of conservation actions are needed to achieve protection of ecological integrity and resilience to climate change for biodiversity, and application of local information is absolutely warranted before specific conservation actions are taken.

Given that context, the American Rivers Protected Rivers Index (PRI, CSP 2025) is an ambitious attempt to connect measures of local protection to each KEA. After studying it extensively, we adopted it in its entirety as an estimate of each stream reach’s local protection. To completely assess protection, however, we combined the local protection level with a measure of regulating function based on the amount of conservation land in the contributing area.

Figure S67. Freshwater protections fall along a spectrum of management intensity and restriction. (Flitcroft et al. 2023).

### Local Protection

The American Rivers’s Protected River Index (PRI) includes five categories of protection, most of which have multiple specific mechanisms. These categories are:

River Conservation

includes Outstanding National Resource Waters, Outstanding Tribal Resource Waters, or equivalent state water quality protection designations, national Wild and Scenic River System designations, and state wild and scenic rivers.

Riparian and Floodplain Conservation Mechanisms

includes riparian buffers; watershed protection areas; riparian, floodplain, and wetland conservation areas; and fishing management areas.

Endangered Species Critical Habitat

Terrestrial Protected Areas

two subclasses – strict and other.

Multiple Land Use

distinguished for special management purposes.

The PRI was calculated by “attributing different weights to each protection mechanism according to their potential conservation effectiveness and accounting for the protection of surrounding lands beyond the river channel.” (CSP 2025) The weights were determined from how fully each mechanism potentially protects each key ecological attribute of freshwater ecosystems (sensu Higgins et al. 2021).

Weights were assigned values between 0 and 1 for each mechanism for each of the five KEAs of hydrologic regime, connectivity, water quality, habitat, and biotic composition for the protection mechanisms. American Rivers graciously made their results available to us linked to the NHD medium resolution arc data (CSP, 2025).

To integrate the American Rivers Protected Rivers Index into our project, we calculated the PRI “over the entire length” of each NHD V2. COMID reach to match our method for calculating upstream conservation land and resilience metrics at the reach level. We placed the “PRI over the entire length of the reach” into 5 classes following the American Rivers binning methodology where those with 0 went into class 5 “No protection” and then the top 4 classes were divided into equal interval groups where each bin included a quarter of the maximum possible score (CSP 2025).

Figure S68. American Rivers Protected Rivers Index applied to full stream and river reaches.

### Protection of Freshwater Provided by Upstream Conservation Lands

Maintaining freshwater in a natural state is arguably the most effective and long-lasting tool for conserving species and habitats. By regulating the use of land and water within a network of places, conservation can sustain species populations and prevent habitat degradation at larger scales. However, because freshwater networks are integrally linked to the surrounding landscape, protection provided by lands managed for conservation depends not just on lands immediately adjacent to freshwater ecosystems, but also the lands and land use in the whole contributing area of the network (Flitcroft et al. 2023). We accounted for the protective benefit of upstream conservation lands to freshwater networks using a method modified from Abell et al. (2017), who argued that the simple intersection of protected areas with rivers misses that fluvial systems are also shaped by the character and condition of their entire catchments. Thus, this complements the PRI assessment of the local protective benefit of conservation lands by considering the protection potential of conservation lands in a reach’s upstream catchment. Conservation lands in the upstream catchment are critical for their regulation of many functions, especially water quality and flow regime.

Our integration of upstream conservation land protection involved three parts. First, we calculated the amount of conservation lands upstream of each reach using The Nature Conservancy’s comprehensive secured lands dataset (Olivero & Clark 2024). Next, we estimated the relative protective benefit of each different category of conservation ownership for regulating function and determined which areas should count toward the score. Third, we translated the results into scores.

#### Definition and Categories of Conservation Land

In this report, “conservation land” refers to land that is permanently secured against conversion to development. This definition was adopted by an international group of American and Canadian scientists to encompass a wide variety of public and private ownership and management types. It goes beyond the more restrictive IUCN definition of “protected areas” which refers to land with a government designation aimed at the conservation of nature. Our definition includes public land designated for conservation but also includes land with no formal designation if the intent of the landowner or easement holder (public, private, or NGO) is for permanent conservation, and if there is a measurable capacity to fulfill the intent. For example, a permanent conservation easement held by an established land trust qualifies as conservation land even though it is privately held and has no designation.

TNC’s Secured Lands dataset is compiled bi-annually from over sixty state, federal, and private sources (Olivero & Clark 2024). The foundation of the dataset is public land information maintained by each state, supplemented by federal land information from PAD-US, and private conservation land information compiled by TNC and the National Conservation Easement Database. We assigned a GAP Status to each tract, populated other standard attribute fields, and quality checked the dataset for consistency and discrepancies across states. Importantly, TNC’s dataset is focused on permanent conservation and only includes land where the intent of the owner or easement holder is conservation in perpetuity and there is some legal capacity to fulfill that intent. Thus, places with important temporary, volunteer, or one-time conservation activities are not included in the dataset. For this analysis, we distinguished six major types of land secured from conversion to development, from most protective to least: GAP 1, GAP 2, GAP 3 non-BLM land, GAP 3 BLM land, State Board Lands, and Permanent Agricultural Easements (Figure S69, Table S16).

Figure S69. Conservation lands in the conterminous U.S. shown by protection class.

Table S16. Conservation land categories used in this analysis.

| **Conservation Land Category** | **Definition** | **Additional Information** |
| --- | --- | --- |
| GAP 1 | Permanently Secured for Nature and Natural Processes. Managed for biodiversity with all natural processes, little to no human intervention. | Primary intention of the owner or easement holder is for biodiversity, nature protection, natural diversity, and natural processes. Land and water managed through natural processes including disturbances with little or no human intervention. Examples: wilderness area, some national parks. |
| GAP 2 | Permanently Secured for Nature with Management: Managed for biodiversity, with hands-on management or interventions. | Primary intention of the owner or easement holder is for biodiversity conservation, nature protection, and natural diversity. Land and water managed for natural biodiversity conservation, but which may include some hands-on manipulation or suppression of disturbance and natural processes. Examples: national wildlife refuges, areas of critical environmental concern, inventoried roadless areas, some natural areas and preserves. |
| GAP 3, non-BLM | Permanently Secured for Multiple Uses, including nature: Primary intention of the owner or easement holder for multiple uses. | Strong focus on recreational use, game species production, timber production, grazing and other uses in addition to these lands providing some biodiversity value. May include extractive uses of a broad, low-intensity type (e.g., some logging, grazing) or of a localized intense type (e.g., mining, military artillery testing area, public access beach area within large natural state park). Examples: recreation focused protected areas such as state parks, state recreation areas, wildlife management areas, game lands, state and national forests. |
| GAP 3, BLM lands | Permanently Secured for Multiple Uses, including nature: Primary intention of the owner or easement holder for multiple uses. | Lands owned by the Bureau of Land Management were placed in a separate category because of the higher level of use and mandate for grazing, mining, and other extractives uses on these lands which often leads to more potential impacts to the ecological systems on these lands. |
| State Board Lands and State Trust Lands | Lands in western and some southern states that are owned by the state and prevented from being developed, but which are managed to produce long term sustained revenue for the state’s educational system. | These lands were separated from other protected multiple use lands in GAP 3. Most of these lands are subject to intensive timber extraction and management for profitable forest product production. Some also have agricultural use and revenue generated from grazing and/or agricultural production leasing. These lands are not specifically managed for biodiversity values. |
| Permanent Agricultural Easements | Conservation land where the primary intent is the preservation of farmland. | Land is in a permanent easement to maintain agricultural cover and/or grass cover. The land will not be converted to a built or paved development. Example: vegetable farm with permanent easement to prevent development. |

Regulating Function

We based the level of regulating function protection for a reach on an assessment of the upstream watershed at three scales:

1) Percent of the upstream drainage area in conservation land,
2) Percent of the upstream river floodplain fluvial area (FATHOM v2 fluvial 100yr floodplain) area in conservation land ,
3) Percent of the local catchment in conservation land.

We used a custom R script with the NHDPlus v2.1 medium resolution reaches and catchments to accumulate the total upstream drainage area in conservation land and the total upstream floodplain in conservation land.

The percent total upstream conservation lands, percent total upstream floodplain conservation lands, and percent local catchment conservation lands were multiplied by weights to adjust the score for the relative level of protection provided by the type of conservation land (Table S17). For example, a reach with 100% of its total upstream area in GAP 2 received a score of 100, a reach with 100% of its total upstream area in GAP 3 non-BLM received a score of 100, and a reach with 100% of its total upstream area in GAP 3 BLM received a score of 25. Each of the 3 spatial scales had a possible maximum score of 100% and minimum of zero.

Table S17. Weighting of conservation land types by assessment unit to calculate the regulating function potential.

| **Conservation Land Type** | **Upstream Drainage Area** | **Upstream Floodplain** | **Local  Catchment** |
| --- | --- | --- | --- |
| GAP 1 | 1 | 1 | 1 |
| GAP 2 | 1 | 1 | 1 |
| GAP 3, non-BLM | 1 | 1 | 1 |
| GAP3, BLM | 0.25 | 0.25 | 0.25 |
| State Land Board lands | 0.25 | NA | 0.25 |
| Permanent Agricultural easements | 0.25 | NA | 0.25 |

We calculated the overall regulating function score by integrating the three scales of assessment and various weights for the types of conservation lands (**Error! Reference source not found.**). First, we calculated the scores for each spatial scale, then we combined these into a single index giving 70% of the weight to the total upstream drainage area. The remaining 30% was distributed differently depending on the streams size. For larger rivers, we gave 20% to the floodplain score and 10% to the local catchment. For smaller rivers, we gave 10% to the floodplain score and 20% to the local catchment. For headwaters and creeks, we gave all 30% to the local catchment score.

We calculated the overall regulating function score by integrating the three scales of source metrics according to the size of the stream or river using the weights in Table S18. First, we calculated the scores for each spatial scale, then we combined these into a single index giving 70% of the weight to the total upstream drainage area. The remaining 30% was distributed differently depending on the streams size. For larger rivers, we gave 20% to the floodplain score and 10% to the local catchment. For smaller rivers, we gave 10% to the floodplain score and 20% to the local catchment. For headwaters and creeks, we gave all 30% to the local catchment score.

The final regulating index was rescaled between 0-100 and we used natural breaks to identify six major classes of regulating function. The lowest scoring class, <10, was given no value for regulating function as inspection suggested they were not meaningful values for protecting significant regulating function. The classes 1-5 were given values ranging from 1 (low protection) to 5 (very high protection) (Figure S70).

Table S18. Integration of the regulating function component metrics.

| **Regulating Function Score** | **Upstream Drainage Area** | **Upstream Floodplain** | **Local Catchment** |
| --- | --- | --- | --- |
| Size 3 + rivers: >1,000 km^2^ drainage area | 0.7 | 0.2 | 0.1 |
| Size 2 rivers: 100-1,000 km^2^ drainage area | 0.7 | 0.1 | 0.2 |
| Streams < 100 km^2^ drainage area | 0.7 |  | 0.3 |

Figure S70. Regulating function protection provided by conservation lands in a reach’s upstream drainage area.

### Protection Class Scoring

To create an overall protection class score for each reach, we combined the PRI local protection score with the regulating function score, giving equal weight to each component and rescaling the result to be between 0 to 100. We group the scores into five protection classes by assigning a score of “No Protection” to all zeros and then dividing the remaining scores into equal interval groups where each bin included a quarter of the maximum possible score (Figure S71). Table S19 shows the percentage of reach length in each of the protection classes by stream size.

Figure S71. Protection class for streams and rivers in CONUS.

Table S19. Percent of stream miles by protection class and by stream size.

| **Class** |  | **Protection** | **Total Percent** | **1. Streams (< 100 km2)** | **2. Small Rivers (100-1,000 km2)** | **3. Medium Rivers (1,000-10,000 km2)** | **4. Large Rivers (10,000-100,000 km2)** | **5. Very Large Rivers (100,000+ km2)** |
| --- | --- | --- | --- | --- | --- | --- | --- | --- |
| 1 |  | High | 4% | 4% | 2% | 1% | 1% | 0% |
| 2 |  | Medium | 11% | 11% | 8% | 10% | 12% | 8% |
| 3 |  | Low | 7% | 6% | 11% | 14% | 14% | 15% |
| 4 |  | Very Low | 29% | 27% | 36% | 41% | 43% | 50% |
| 5 |  | None | 51% | 52% | 43% | 33% | 30% | 26% |
|  |  | **Grand Total** | **100%** | **100%** | **100%** | **100%** | **100%** | **100%** |

### Calculating Conservation Status

We combined protection class with the freshwater resilience analysis to answer two critical questions. First, what is the conservation status of all rivers in CONUS? Second, what is the conservation status of the nation’s most resilient rivers?

#### Conservation Status for all Flowlines

For all flowlines, we combined the seven resilience categories and five protection categories, which resulted in 34 unique combinations of classes. We then distilled these classes into six major groups (Table S20).

Our results (Table S21, Figure S72, Figure S73) show that only 7% of the flowlines in CONUS are both resilient and have a high or medium level of protection. Another 23% of flowlines are resilient but have either low, very low, or no protection from the mechanisms examined here. The remaining 70% of flowlines need significant restoration to be resilient. The group that are average for resilience (green and tan) are mostly not protected (50% unprotected compared to 6% protected). The remaining more vulnerable stream reaches (medium and dark brown totaling 14%) are also mostly unprotected (13%).

Table S20. Conservation status levels for all streams.

| **Resilience and Protection Class** | **Resilience** | **Protection** |
| --- | --- | --- |
| Meets Resilience and Protection | Greater than Average | High or Medium |
| Meets Resilience but Needs Protection | Greater than Average | Low, Very Low, None |
| Needs Restoration of Resilience but has Protection | Average | High or Medium |
| Needs Restoration of Resilience and Needs Protection | Average | Low, Very Low, None |
| Needs Much Restoration of Resilience but has Protection | Less than Average | High or Medium |
| Needs Much Restoration of Resilience and Needs Protection | Less than Average | Low, Very Low, None |

Figure S72. Conservation status of all streams in CONUS based on freshwater resilience and level of protection.

Table S21. All flowline conservation status percentages summarized by protection and resilience class and stream size.

| **Conservation Status Class** | **Total Percent** | **1. Streams (< 100 km2)** | **2. Small Rivers (100-1,000 km2)** | **3. Medium Rivers (1,000-10,000 km2)** | **4. Large Rivers (10,000-100,000 km2)** | **5. Very Large Rivers (100,000+ km2)** |
| --- | --- | --- | --- | --- | --- | --- |
| 1. Meets Resilience and Protection | 7% | 7% | 6% | 5% | 5% | 1% |
| 2. Meets Resilience but Needs Protection | 23% | 22% | 25% | 22% | 14% | 7% |
| 3. Needs Restoration of Resilience but has Protection | 6% | 6% | 3% | 5% | 7% | 7% |
| 4. Needs Restoration of Resilience and Needs Protection | 50% | 50% | 51% | 55% | 62% | 77% |
| 5. Needs Much Restoration of Resilience but has Protection | 1% | 2% | 1% | 1% | 1% | 0% |
| 6. Needs Much Restoration of Resilience and Needs Protection | 13% | 13% | 14% | 12% | 11% | 8% |
| Grand Total | 100% | 100% | 100% | 100% | 100% | 100% |

Figure S73. The distribution of conservation status of all flowlines in CONUS.

The horizontal axis is the freshwater resilience score from “Far Below Average” (FBA) to “Far Above Average” (FAA) resilience. Average is centered. The vertical axis is the level of protection from none to high, using the six classes shown in Figure S72. The size of the sphere reflects the percentage of the stream reaches that fall into each category. Finally, the colored circles correspond to the conservation status groupings in Table S20.

#### Conservation Status of the Freshwater Resilient and Connected Network

When we intersected the protection classes with the FRCN flowlines, we found that 16% of the FRCN flowlines are both resilient and have a high or medium level of protection (dark blue). Another 39% of flowlines are resilient but have either low, very low, or no protection from the mechanisms examined (light blue). The remaining FRCN groups that are average for resilience are mostly unprotected (39%, tan), while a small percentage (6%) are protected (green) (Table S22, Figure S74, Figure S75).

Figure S74. Conservation status of the Freshwater Resilient and Connected Network (FRCN) shown by resilience and protection class.

Table S22. FRCN conservation status percentages summarized by protection and resilience class and stream size.

| **Conservation Status Class** | **Total percent** | **1. Streams (< 100 km2)** | **2. Small Rivers (100-1,000 km2)** | **3. Medium Rivers (1,000-10,000 km2)** | **4. Large Rivers (10,000-100,000 km2)** | **5. Very Large Rivers (100,000+ km2)** |
| --- | --- | --- | --- | --- | --- | --- |
| 1. Meets Resilience and Protection | 16% | 17% | 14% | 10% | 7% | 2% |
| 2. Meets Resilience but Needs Protection | 39% | 40% | 42% | 31% | 18% | 8% |
| 3. Needs Restoration of Resilience but has Protection | 6% | 6% | 4% | 7% | 10% | 8% |
| 4. Needs Restoration of Resilience and Needs Protection | 39% | 37% | 40% | 52% | 65% | 82% |
| Grand Total | 100% | 100% | 100% | 100% | 100% | 100% |

Figure S75. The distribution of conservation status of the FRCN in CONUS.

The horizontal axis is the freshwater resilience score from “Far Below Average” (FBA) to “Far Above Average” (FAA) resilience. Average is centered. The vertical axis is the level of protection from none to high, using the four classes shown in Figure S74. The size of the sphere reflects the percentage of the stream reaches that fall into each category. Finally, the colored circles correspond to the conservation status groupings in Table S22.

# Recognized Biodiversity Data Sources

Table S23. State plans and analyses of freshwater biodiversity.

| **State** | **Plan Title & Date** | **Link to Source** |
| --- | --- | --- |
| AR | Ecologically Sensitive Waters | <https://gis.arkansas.gov/product/ecologically-sensitive-streams-line/> |
| CA | Howard et al. (2018) A Freshwater Conservation Blueprint for California | <https://www.journals.uchicago.edu/doi/full/10.1086/697996>  <https://databasin.org/datasets/b03819ca45bc46aa912966bb062763ee/> |
| CO | Colorado (2015): Crucial Habitat for Tier 1 Terrestrial Animal and Plant SGCN (Figure 21). | <http://cpw.state.co.us/aboutus/Pages/StateWildlifeActionPlan.aspx> |
| FL | Florida (2016) Priority 1 and 2 CLIP V.4 Significant Surface Waters | [https://www.fnai.org/services/clip https://www.fnai.org/pdf/CLIP_v4_technical_report.pdf](https://www.fnai.org/services/clip) |
| GA | Georgie (2015). SWAP High Priority Watersheds Priority Conservation Areas. | <https://georgiawildlife.com/sites/default/files/wrd/pdf/swap/appendix-f-aquatic-habitat-technical-team-report-high-priority-watersheds.pdf> |
| IL | Illinois (2016): COAs currently recognized through the Illinois Wildlife Action Plan (Figure 1). | <https://www.dnr.illinois.gov/conservation/iwap/pages/default.aspx> |
| IN | Indiana (2015): Indiana conservation opportunity areas | <https://www.in.gov/dnr/fishwild/7580.htm> |

| **State** | **Plan Title & Date** | **Link to Source** |
| --- | --- | --- |
| KS | Kansas (2016): Aquatic Ecological Focal Areas | <https://www.sciencebase.gov/catalog/item/58498170e4b06d80b7b094c7https://ksoutdoors.com/Services/Kansas-SWAP> |
| KY | Kentucky (2013) State Wildlife Action Plan | <https://fw.ky.gov/WAP/Pages/default.aspx> ; map downloaded from <ftp://ftp.kymartian.ky.gov/kdfwr/swap/huc8_fish_bivalve_overlap.zip> |
| LA | Louisiana (2015) Conservation Opportunity Areas. | <https://www.wlf.louisiana.gov/assets/Conservation/SWG/Files/17_WAP_2017_Ch_8.pdf> |
| MA | Massachusetts (2022) BioMap3 | <https://biomap-mass-eoeea.hub.arcgis.com/> |
| MI | Cohen (2011) Michigan: Biodiversity Stewardship Areas | <https://mnfi.anr.msu.edu/reports/MNFI-Report-2011-08.pdf> |
| MN | Minnesota (2015): The Wildlife Action Network map, | PrioritizedNetwork_Aquatic_20150914.SHP - file from MN DNR Nongame Wildlife Program; Priority Rank (gridcode=4) <https://www.dnr.state.mn.us/mnwap/index.html> |
| MS | Mississippi (2015) Mississippi Conservation Opportunity Areas | Mississippi Conservation Opportunity Areas: Geospatial Data Presentation Form: vector digital data <https://www.sciencebase.gov/catalog/item/5849874be4b06d80b7b094fa> |
| MO | Missouri (2015): 2015 Conservation Opportunity Areas | <https://mdc.mo.gov/sites/default/files/downloads/SWAPopt.pdf> |
| MT | Montana (2015): Aquatic Focal Areas | <http://fwp.mt.gov/fishAndWildlife/conservationInAction/actionPlan.html> |
| NE | Nebraska (2015): Nebraska Natural Legacy Project: Biologically Unique Landscapes and Demonstration Sites | <http://outdoornebraska.gov/naturallegacyproject/> |
| NV | Nevada (2017) Wildlife Action Plan. | <https://www.ndow.org/wp-content/uploads/2022/01/2013-NV-WAP-Complete-NOT-ADA.pdf> |
| NH | New Hampshire (2015): WAP | <https://www.wildlife.state.nh.us/wildlife/wap.html> |
| NM | New Mexico (2016): Conservation Opportunity Areas (Fig. 11) | <https://nmswap.org> |

| **State** | **Plan Title & Date** | **Link to Source** |
| --- | --- | --- |
| NC | North Carolina (2015) State Wildlife Action Plan | <https://www.ncwildlife.org/plan> |
| ND | North Dakota (2015): North Dakota State Wildlife Plan focal areas | <https://gf.nd.gov/wildlife/swap> |
| OH | Ohio (2015): State Wildlife Action Plan | Ohio COAs aquatic subset - <https://www.sciencebase.gov/catalog/item/58499154e4b06d80b7b09547>; <http://wildlife.ohiodnr.gov/ohioswap> |
| OK | WAFWA (2013): Critical Habitat Assessment Tool - Oklahoma | <https://www.wafwachat.org/> ;  see also: <https://www.wildlifedepartment.com/sites/default/files/2021-11/Oklahoma%20Comprehensive%20Wildlife%20Conservation%20Strategy.pdf> |
| OR | Oregon (2016): Oregon Conservation Strategy | <https://oregonconservationstrategy.org/> |
| PA | Pennsylvania (2011). Conservation Opportunity Areas. | Pennsylvania Conservation Opportunity Areas from <https://www.sciencebase.gov/catalog/item/584991a4e4b06d80b7b0954b> |
| SD | South Dakota (2015): Wildlife Action Plan | <https://gfp.sd.gov/UserDocs/WAPCh4_AquaticSystems.pdf>  <https://gfp.sd.gov/wildlife-action-plan/> |
| TN | Tennessee (2015) SWAP | <https://www.tn.gov/content/tn/twra/wildlife/action-plan/tennessee-wildlife-action-plan.html> |
| TX | WAFWA (2018): Critical Habitat Assessment Tool - Texas | from WAFWACHAT.org |
| VT | Vermont (2018) Vermont Conservation Design | <https://vtfishandwildlife.com/sites/fishandwildlife/files/documents/Conserve/VT%20Conservation%20Landscape-level%20Design/Vermont-Conservation-Design-Summary-Report-February-2018.pdf>   This feature layer is displayed on the Biofinder website at https://anrmaps.vermont.gov/websites/BioFinder/ under Species Components, Aquatic Habitats – Highest Priority. |
| WI | Wisconsin 2015: Wisconsin Conservation Opportunity Areas | <https://dnr.wi.gov/topic/wildlifehabitat/actionplan.html> A compiled statewide map is here: <https://dnr.wi.gov/topic/WildlifeHabitat/documents/MapCOA_statewide.pdf> |
| WY | Wyoming (2010): Conservation Opportunity Areas | wgfd.maps.arcgis.com  Links to the 2017 and the 2010 plan: <https://wgfd.wyo.gov/Habitat/Habitat-Plans/Wyoming-State-Wildlife-Action-Plan> |

Table S24. Sources for species data in Recognized Biodiversity Value.

| **Data Layer** | **Origin** | **Link to Source** |
| --- | --- | --- |
| Critical Habitat for Salmon, Steelhead, Green Sturgeon | NOAA Fisheries - Critical Habitat - Maps and GIS Data (West Coast Region) | <https://www.fisheries.noaa.gov/resource/map/critical-habitat-maps-and-gis-data-west-coast-region> |
| Bull Trout strongholds | Climate Shield (Isaaks et al. 2017, 2022) | <https://www.fs.usda.gov/rm/boise/AWAE/projects/ClimateShield/maps.html> |
| Atlantic Salmon Critical Habitat in Maine | NOAA 2023 | <https://www.fisheries.noaa.gov/resource/map/atlantic-salmon-gulf-maine-dps-critical-habitat-map-and-gis-data> |
| Atlantic Subspecies Atlantic Sturgeon | NOAA 2023 | <https://www.fisheries.noaa.gov/resource/map/atlantic-sturgeon-critical-habitat-map-and-gis-data> |
| Eastern Brook Trout Secure strongholds | Trout Unlimited (Fesenmyer et al. 2017) | <https://www.tu.org/science/conservation-planning-and-assessment/conservation-portfolio/eastern-brook-trout-conservation-portfolio/> |
| TNC Atlantic Coast Alosine Prioritization (top 5%) | Martin (2019) Atlantic Coast Whole System Diadromous Fish Prioritization | <https://www.atlanticfishhabitat.org/wp-content/uploads/2019/12/TNC_AtlanticCoast_AlosinePrioritization.pdf> |
| Chesapeake Alosine occurrences | Martin, E. H. 2019. Chesapeake Fish Passage Prioritization: An Assessment of Dams in the Chesapeake Bay Watershed. The Nature Conservancy.  Data is based on the professional opinion of the Chesapeake workgroup (which includes the fish passage coordinators in MD, VA & PA, USFWS & NOAA staff). | <https://maps.freshwaternetwork.org/chesapeake/> |
| Critical Habitat for inland aquatic species | USFWS 2023 | <https://gis-fws.opendata.arcgis.com/maps/794de45b9d774d21aed3bf9b5313ee24/about> |

# Steering Committee

This work would not have been possible without the contributions of colleagues across The Nature Conservancy (TNC) and beyond. From champions to challengers, we are grateful for everyone’s input. At the risk of leaving someone out, below are listed the colleagues who advocated for and participated in mapping freshwater resilience for the conterminous U.S.

Michael Reuter, Midwest Division Director

Michael Lipford, former Southern Division Director

Terry Sullivan, former Northeast Division Director

Freshwater Advisory Committee

Robin Abel, TNC Durable Freshwater Protection Director

Allison Aldous, TNC Global Provide

Roshni Arora, TNC India

Alison Bowden, TNC Massachusetts

Jonathan Higgins, formerly TNC Global Protect

Jeanette Howard, TNC California

Nathan Karres, TNC Global Provide

Patrick McCarthy, formerly TNC Colorado River Program

Paulo Petry, TNC Latin America Region

Meg White, formerly TNC North America Region

Mississippi and Great Plains Steering Committee

Danna Baxley, TNC Kentucky

Kristen Blann, TNC Minnesota/Dakotas

Rob Bullard, TNC Tennessee

Darren Crabtree, formerly TNC New York Division

Jeff Fore, TNC Arkansas

Cassie Hauswald, formerly TNC Indiana

Matt Herbert, TNC Michigan

Steve Herrington, TNC Minnesota/Dakotas

Suzanne Hickey, formerly TNC Iowa

Amy Holtshouse, TNC Ohio

Nathan Korb, formerly TNC Montana

Courtney Larson, TNC Wyoming

Maria Lemke, TNC Illinois

Scott Lemmons, TNC Mississippi

Heidi Mehl, TNC Kansas

Chris Pague, TNC Colorado

Bryan Piazza, TNC Louisiana

Terri Schulz, TNC Colorado*

Ryan Smith, TNC Texas*

Scott Sowa, TNC Great Lakes Program

Rip Sparks, TNC Illinois Science Advisory Committee

Josh Spies, TNC Iowa

John Wagner, TNC Wisconsin

Rick Walters, TNC Nebraska

*also served on Western U.S. Steering Committee

Eastern U.S. Steering Committee

Braven Beaty, TNC Virginia

Dirk Bryant, TNC New York Division

Mark Bryer, TNC Northeast Division Director

Barbara Charry, TNC Massachusetts

Chuck DeCurtis, TNC New Hampshire

Julie DeMeester, TNC North Carolina

Holly Drinkuth, TNC Connecticut

Su Fanok, TNC Pennsylvania

Sara Gottlieb, TNC Southern Appalachians Program, formerly TNC Georgia

Katie Kennedy, TNC North America Region

Eric Krueger, TNC South Carolina

Beth Lewis, TNC Florida

Josh Royte, TNC Maine

Danica Schaffer – Smith, formerly TNC North Carolina

George Shuler, formerly TNC New York Division

Sarah Whateley, formerly TNC New York Division

Western U.S. Steering Committee

Martha Cooper, TNC New Mexico

Emily Howe, TNC Washington

Aaron Jones, TNC New Mexico

Jessie Pearl, TNC Arizona

Louis Provencher, TNC Nevada (also represented Utah)

Holly Richter, formerly TNC Arizona

Corinna Riginos, TNC Wyoming

Claire Ruffing, TNC Oregon

Laurel Saito, TNC Nevada

Melissa Stamp, Utah Reclamation Mitigation and Conservation Commission

Joe Trungale, TNC Colorado River Program

Nathan Welch, formerly TNC Idaho

# Supplementary References

Abell, R., Lehner, B., Thieme, M., and S. Linke. 2017. Looking Beyond the Fenceline: Assessing Protection Gaps for the World’s Rivers, Conservation Letters, 10(4). DOI: 10.1111/conl.12312

Abood, S.A., Spencer, L., Wieczorek, M. 2022. U.S. Forest Service national riparian areas base map for the conterminous United States in 2019. Fort Collins, CO: Forest Service Research Data Archive. <https://doi.org/10.2737/RDS-2019-0030>

Alexander, L.C., Fritz, K.M., Schofield, K.A., Autrey, B.C., DeMeester, J.E., Golden, H.E., Goodrich, D.C., Kepner, W.G., Kiperwas, H.R., Lane, C.R., LeDuc, S.D., Leibowitz, S.G., McManus, M.G., Pollard, A.I., Ridley, C.E., Vanderhoof, M.K., and Wigington, P.J. 2018. Featured Collection Introduction: Connectivity of Streams and Wetlands to Downstream Waters. *Journal of the American Water Resources Association*, 54(2), 287–297. <https://doi.org/10.1111/1752-1688.12630>.

Allan, J. D. 1995. Stream Ecology: Structure and function of running waters. Kluwer Academic Publishers. Dordrecht, The Netherlands.

Anderson, M.G., M. Clark, A. Olivero, and J. Prince. 2019. Resilient Sites and Connected Landscapes for Terrestrial Conservation in the Rocky Mountain and Southwest Desert Region. The Nature Conservancy, Eastern Conservation Science.

Barnhart, T.B., Molotch, N.P., Livneh, B., Harpold, A.A., Knowles, J.F. and Schneider, D. 2016. Snowmelt rate dictates streamflow. Geophysical Research Letters, 43(15), pp.8006-8016.

Bates, P. D., Quinn, N., Sampson, C., Smith, A., Wing, O., Sosa, J., et al. 2021. Combined modeling of US fluvial, pluvial, and coastal flood hazard under current and future climates. Water Resources Research, 57, e2020WR028673. https://doi.org/10.1029/2020WR028673

Bowler, D. E., Mant, R., Orr, H., Hannah, D. M., and Pullin, A. S. 2012. What are the effects of wooded riparian zones on stream temperature? Environmental Evidence, 1(1), 1–9. <https://doi.org/10.1186/2047-2382-1-3>.

Center for Watershed Protection (CWP). 2003. Impacts of Impervious Cover on Aquatic Systems. Watershed Protection Research monograph No. 1.

Chithra, S.V., Harindranathan Nair, M.V., Amarnath, A., and Anjana, N.S. 2015. Impacts of Impervious Surfaces on the Environment. International Journal of Engineering Science Invention, 4(5), 27–31.

Comer, Patrick. 2022. Crosswalk of Landfire biophysical systems to Upper Montane, Subalpine, and Alpine Ecosystems. Personal Communication from Senior Ecologist, NatureServe.

Comte, L., and Grenouillet, G. 2013. Do stream fish track climate change? Assessing distribution shifts in recent decades. Ecography, 36(11), 1236–1246. https://doi.org/10.1111/j.1600-0587.2013.00282.x.

Comte, L., Murienne, J., and Grenouillet, G. 2014. Species traits and phylogenetic conservatism of climate-induced range shifts in stream fishes. Nature Communications, 5, 5053. <https://doi.org/10.1038/ncomms6053>

Conservation Science Partners (CSP). 2025. Protected Rivers Assessment of the United States - Final Report. Truckee, California, USA.

de Mello K., Valente R.A, Randhir T.O., Alves dos Santos A.C., and Vettorazzi C.A. 2018. Effects of land use and land cover on water quality of low-order streams in southeastern Brazil: watershed versus riparian zone. Catena 167: 130-138. <https://doi.org/10.1016/j.catena.2018.04.027>.

Dewitz, J., and U.S. Geological Survey, 2021, National Land Cover Database (NLCD) 2019 Products (ver. 2.0, June 2021): U.S. Geological Survey data release, https://doi.org/10.5066/P9KZCM54.

Doctor, D.H., Jones, J., Wood, N, Falgout, J., and Rapstine, N. 2020. Progress toward a Preliminary Karst Depression Density Map for the Conterminous United States. NCKRI Symposium 8: Proceedings of the 16th Multidisciplinary Conference on Sinkholes and the Engineering and Environmental Impacts of Karst. 315-326

Dodds, W., Gido, K., Whiles, M.R., Fritz, K.M., and Matthews, W.J. 2004. Life on the edge: the ecology of Great Plains prairie streams. BioScience, 54(3), 205–216. <https://academic.oup.com/bioscience/article/54/3/205/22306>

Falcone, J.A., Murphy, J.C., and Sprague, L.A. 2018. Regional patterns of anthropogenic influences on streams and rivers in the conterminous United States, from the early 1970s to 2012. Journal of Land Use Science. 13:6, 585-614, doi: 10.1080/1747423X.2019.1590473

Federal Interagency Stream Restoration Working Group (FISRWG). 1998. Stream Corridor Restoration: Principles, Processes, and Practices. GPO Item No. 0120-A; SuDocs No. A 57.6/2:EN3/PT.653. ISBN-0-934213-59-3.

First Street Foundation. 2020. First Street Foundation Flood Model Technical Methodology Document. Zenoda. https://zenodo.org/record/4740762CDFW.

Flitcroft, R. L., Abell, R., Harrison, I., Arismendi, I., & Penaluna, B. E. (2023). Making global targets local for freshwater protection. *Nature Sustainability*. https://doi.org/10.1038/s41893-023-01193-7

Florida Department of Environmental Protection (FDEP), Division of Water Resource Management. 2021. Mandatory Phosphate Reclamation Units 2019 vector digital data. https://publicfiles.dep.state.fl.us/OTIS/GIS/data/MMP_MANPHO_REC_UNITS_2019.zip

Frei, R.J., Lawson, G.M., Norris, A.J., Cano, G., Vargas, M. C., Kujanpää, E., Hopkins, A., Brown, B., Sabo, R., Brahney, J., & Abbott, B.W. 2021. Limited progress in nutrient pollution in the U. S. caused by spatially persistent nutrient sources. PLoS ONE, 16(11 November), 1–26. <https://doi.org/10.1371/journal.pone.0258952>.

Getirana, A., Rodell, M., Kumar, S., Beaudoing, H.K., Arsenault, K., Zaitchik, B., Save, H., & Bettadpur, S. 2020. GRACE improves seasonal groundwater forecast initialization over the United States. Journal of Hydrometeorology, 21(1), 59–71. https://doi.org/10.1175/JHM-D-19-0096.1.

Gido, K.B., Dodds, W.K., and Eberle, M.E. 2010. Retrospective analysis of fish community change during a half-century of landuse and streamflow changes. Journal of the North American Benthological Society, 29(3), 970–987. <https://doi.org/10.1899/09-116.1>

Hare, D. K., Helton, A. M., Johnson, Z. C., Lane, J. W., & Briggs, M. A. 2021. Continental-scale analysis of shallow and deep groundwater contributions to streams. Nature Communications, 12(1), 1–10. https://doi.org/10.1038/s41467-021-21651-0

Higgins, J.V., Bryer, M.T., Khoury, M.L. and Fitzhugh, T.W. 2005. A Freshwater Classification Approach for Biodiversity Conservation Planning. Conservation Biology, 19, 432-445. <https://doi.org/10.1111/j.1523-1739.2005.00504.x>.

Higgins, J., Zablocki, J., Newsock, A., Krolopp, A., Tabas, P., & Salama, M. (2021). Durable freshwater protection: A framework for establishing and maintaining long-term protection for freshwater ecosystems and the values they sustain. Sustainability (Switzerland), 13(4), 1–17. https://doi.org/10.3390/su13041950.

Hill, R.A., Hawkins, C.P., and Jin, J. 2014. Predicting thermal vulnerability of stream and river ecosystems to climate change. Climatic Change 125(3): 399-412

Hill, R.A., Weber, M.H., Leibowitz, S.G., Olsen, A.R. & Thornbrugh, D.J. 2016. The Stream‐Catchment (StreamCat) Dataset: A database of watershed metrics for the conterminous United States. J Am Water Resour Assoc 52, 120–128.

Horton, J.D. 2017. The state geologic map compilation (SGMC) geodatabase of the conterminous United States. https://doi.org/10.3133/ds1052 http://pubs.usgs.gov/ds/425</onlink>

https://www.safewater.org/fact-sheets-1/2017/1/23/miningandwaterpollution. Accessed October 5, 2023.

Houborg, R., Rodell, M., Li, B., Reichle, R., & Zaitchik, B. F. 2012. Drought indicators based on model-assimilated Gravity Recovery and Climate Experiment (GRACE) terrestrial water storage observations. Water Resources Research, 48(7). https://doi.org/10.1029/2011WR01129.

Jones Jr, C.E., Leibowitz, S.G., Sawicz, K.A., Comeleo, R.L., Stratton, L. E., Morefield, P.E., & Weaver, C.P. 2021. Using hydrologic landscape classification and climatic time series to assess hydrologic vulnerability of the western US to climate. Hydrology and earth system sciences, 25(6), 3179-3206.

Juracek, K. E. 2015. The aging of America’s reservoirs: in-reservoir and downstream physical changes and habitat implications. Journal of the American Water Resources Association, 51(1), 168–184. https://doi.org/10.1111/jawr.12238

Kaufmann, P.R., Hughes, R.M., Paulsen, S.G., Peck, D.V., Seeliger, C.W., Weber, M.H., & Mitchell, R.M. 2022. Physical habitat in conterminous US streams and rivers, Part 1: Geoclimatic controls and anthropogenic alteration. Ecological Indicators, 141(January), 109046. https://doi.org/10.1016/j.ecolind.2022.109046

King, R.S. and M.W. Baker. 2010. Considerations for analyzing ecological community thresholds in response to anthropogenic environmental gradients. Journal of the North American Benthological Society. 29(4): 998-1008.

LANDFIRE. 2022. Biophysical Setting Layer (LC16_BpS_200). LANDFIRE 2016 REMAP LF 2.0.0 (U.S. Department of the Interior, Geological Survey, and U.S. Department of Agriculture, Washington, DC, 2016). https://www.landfire.gov/viewer/. Accessed 11 May 2022.

LANDFIRE. 2020. Existing Vegetation Type Layer (EVT), LANDFIRE 2.2.0, U.S. Department of the Interior, Geological Survey, and U.S. Department of Agriculture. Accessed at <http://www.landfire/viewer>.

Lyons, J., Zorn, T., Stewart, J., Seelbach, P., Wehrly, K., and Wang, L. 2009. Defining and Characterizing coolwater streams and their fish assemblages in Michigan and Wisconsin, USA. North American Journal of Fisheries Management, 29(4), 1130–1151. https://doi.org/10.1577/M08-118.1

Mid-America Regional Council (MARC). 2023. What is Sediment Pollution? https://cfpub.epa.gov/npstbx/files/ksmo_sediment.pdf Accessed September 15, 2023.

Manning, D. W. P., A. D. Rosemond, J. P. Benstead, P. M. Bumpers, and J. S. Kominoski. 2020. Transport of N and P in U.S. streams and rivers differs with land use and between dissolved and particulate forms. Ecological Applications 30(6):e02130. 10.1002/eap.2130.

Martin, E. 2019. Atlantic Coast Whole System Diadromous Fish Prioritization. <https://www.atlanticfishhabitat.org/wpcontent/uploads/2019/12/TNC_AtlanticCoast_AlosinePrioritization.pdf>

Maus, V., Giljum, S., Gutschlhofer, J. et al. 2020. A global-scale data set of mining areas. Sci Data 7, 289. https://doi.org/10.1038/s41597-020-00624-w

Maus, V., Giljum, S., da Silva, D.M. et al. 2022. An update on global mining land use. Sci Data 9, 433. https://doi.org/10.1038/s41597-022-01547-4

Mayer, T. D. & S. W. Naman. 2011. Streamflow response to climate as influenced by geology and elevation. Journal of the American Water Resources Association, 47(4), 724–738. https://doi.org/10.1111/j.1752-1688.2011.00537.x

McElfish, J.A. (2023). What Comes Next for Clean Water? Six Consequences of Sackett v. EPA. Environmental Law Institute. https://www.eli.org/vibrant-environment-blog/what-comes-next-clean-water-six-consequences-sackett-v-epa. Accessed September 18, 2024.

McManamay, R.A. and DeRolph, C.R. 2019. A stream classification system for the conterminous United States. Scientific Data, 6(1), pp.1-18.

McManamay, R. A., George, R., Morrison, R. R., & Ruddell, B. L. 2022. Mapping hydrologic alteration and ecological consequences in stream reaches of the conterminous United States. Sci Data 9, 1–14. https://doi.org/10.1038/s41597-022-01566-1.

McManamay R.A., Troia M.J., DeRolph C.R., Olivero Sheldon A, Barnett, Kao S-C, et al. 2018. A stream classification system to explore the physical habitat diversity and anthropogenic impacts in riverscapes of the eastern United States. PLoS ONE, 13(6): e0198439. https://doi.org/10.1371/journal.pone.0198439

Miller, M. P., S. G. Buto, D. D. Susong, and C. A. Rumsey. 2016. The importance of base flow in sustaining surface water flow in the Upper Colorado River Basin, Water Resources Research, 52:3547–3562.

Miller, O.L., Wise, D.R., and Anning, D.W. 2020. SPARROW model inputs and simulated streamflow, nutrient and suspended sediment loads in streams of the Southwestern United States, 2012 Base Year: U.S. Geological Survey data release, https://doi.org/10.5066/P9GFLBG8.

Murphy, J. 2020. Changing suspended sediment in United States rivers and streams: linking sediment trends to changes in land use/cover, hydrology, and climate. Hydrol. Earth Syst. Sci., 24, 991–1010, 2020. <https://doi.org/10.5194/hess-24-991-2020>

Olivero, A. and M. Clark. 2024. Secured Lands of the Conterminous United States dataset. Center for Resilient Conservation Science, The Nature Conservancy.

Olivero-Sheldon, A. and M.G. Anderson. 2016. Northeast Lake and Pond Classification System. The Nature Conservancy, Eastern Conservation Science, Eastern Regional Office. Boston, MA.

Olivero-Sheldon, A., Barnett, A. & Anderson, M. G. 2015. A stream classification for the Appalachian Region. The Nature Conservancy, Eastern Conservation Science, Eastern Regional Office: Boston, MA https://www.conservationgateway.org/ConservationByGeography/NorthAmerica/United States /edc/reportsdata/freshwater/habitat/Pages/Appalachian-Stream-Classification.aspx 2015.

Paukert, C., Olden, J.D., Lynch, A.J., Breshears, D.D., Christopher Chambers, R., Chu, C., Daly, M., Dibble, K.L., Falke, J., Issak, D., Jacobson, P., Jensen, O. P., & Munroe, D. 2021. Climate change effects on North American fish and fisheries to inform adaptation strategies. Fisheries, 46(9), 449–464. https://doi.org/10.1002/fsh.10668

Pelletier, M. C., Ebersole, J., Mulvaney, K., Rashleigh, B., Gutierrez, M. N., Chintala, M., Kuhn, A., Molina, M., Bagley, M., & Lane, C. 2020. Resilience of aquatic systems: Review and management implications. Aquatic Sciences, 82(2), 1–44. https://doi.org/10.1007/s00027-020-00717-z.

Pengra, B.W., Stehman, S.V., Horton, J.A., Auch, R.F., Dockter, D.J., Kambly, S., and Taylor, J.L. 2021. LCMAP CONUS Intensification Reference Data Product 1984-2019 land cover, land use and change process attributes: U.S. Geological Survey data release, https://doi.org/10.5066/P9QA5Q25.

Pericak A.A., Thomas, C.J., Kroodsma, D.A., Wasson, M.F., Ross, M.R.V., Clinton, N.E., et al. 2018. Mapping the yearly extent of surface coal mining in Central Appalachia using Landsat and Google Earth Engine. PLoS ONE, 13(7): e0197758. https://doi.org/10.1371/journal.pone.0197758.

Piggott, J.J., Townsend, C.R., & Matthaei, C.D. 2015. Climate warming and agricultural stressors interact to determine stream macroinvertebrate community dynamics. Global Change Biology, 21(5), 1887–1906. https://doi.org/10.1111/gcb.12861.

Poff, N. L., Allan, J. D., Bain, M. B., Karr, J. R., Prestegaard, K. L., Richter, B. D., Sparks, R. E., & Stromberg, J. C. (1997). The Natural Flow Regime. *BioScience*, *47*(11), 769–784. https://doi.org/10.2307/1313099

Possingham, H. P., Bode, M., & Klein, C. J. (2015). Optimal Conservation Outcomes Require Both Restoration and Protection. *PLoS Biology*, *13*(1), 1–15. https://doi.org/10.1371/journal.pbio.1002052

Pringle, C.M. 2001. Hydrologic connectivity and the management of biological reserves: a global perspective. Ecological Applications, 11(4), 981–998.

Pringle, C. 2003. What is hydrologic connectivity and why is it ecologically important? Hydrological Processes, 17(13), 2685–2689. https://doi.org/10.1002/hyp.5145.

Provencher, L., Blankenship, K., and Byer, S. 2021. Handbook for Mapping High-Elevation Forest Thinning Opportunities to Increase Runoff in the Colorado River Basin Above Lake Mead: A Report to the Colorado River Program’s Forest and Snow Management Team. The Nature Conservancy, Reno NV. 31p.

Rieman, B. E., & Isaak, D. J. 2010. Climate change, aquatic ecosystems, and fishes in the rocky mountain west: Implications and alternatives for management. USDA Forest Service - General Technical Report RMRS-GTR, 250, 1–53. https://doi.org/10.2737/RMRS-GTR-250

Robertson, D.M., and Saad, D.A. 2019. Spatially referenced models of streamflow and nitrogen, phosphorus, and suspended-sediment loads in streams of the Midwestern United States: U.S. Geological Survey Scientific Investigations Report 2019–5114, 74 p. including 5 appendixes, <https://doi.org/10.3133/sir20195114>.

Roland, V.L., II, and Hoos, A.B. 2020. SPARROW model inputs and simulated streamflow, nutrient and suspended-sediment loads in streams of the Southeastern United States, 2012 Base Year: U.S. Geological Survey data release, https://doi.org/10.5066/P9A682GW.

Rosgen, D. L. 1994. A classification of natural rivers. Catena 22, 169–199.

Rosgen, D.L. 1996. Applied River Morphology. Wildland Hydrology, Pagosa Springs, CO.

Saad, D.A., and Robertson, D.M. 2020. SPARROW model inputs and simulated streamflow, nutrient and suspended-sediment loads in streams of the Midwestern United States, 2012 Base Year: U.S. Geological Survey data release, https://doi.org/10.5066/P93QMXC9.

Santhi, C.P., Allen, P., Muttiah, R., Arnold, J. & Tuppad, P. 2008. Regional estimation of base flow for the conterminous United States by hydrologic landscape regions. Journal of Hydrology, 351:139-153. 10.1016/j.jhydrol.2007.12.018.

Safe Drinking Water Foundation (SDWF). 2017. Mining and Water Pollution Fact Sheet. Available from: https://www.safewater.org/fact-sheets-1/2017/1/23/miningandwaterpollution. Accessed September 2023.

Soller, D.R. 2009. Map database for surficial materials in the Conterminous United States (Geologic units, 1:5,000,000). Vector digital data.

Soller, D.R., Packard, P.H., and Garrity, C.P. 2012. Database for USGS Map I-1970 — Map showing the thickness and character of Quaternary sediments in the glaciated United States east of the Rocky Mountains: U.S. Geological Survey Data Series 656, available at https://pubs.usgs.gov/ds/656/.

Soulard, C.E., Acevedo, W., Stehman, S.V., and Parker, O.P. 2016. Mapping extent and change in surface mines within the United States for 2001 to 2006. Land Degradation and Development, 27 (2): 248-257.

Starnes, L.B. and Gasper, D.C. 1995. Effects of Surface Mining on Aquatic Resources in North America (Revised, Full Text). American Fisheries Society (AFS) Policy Statement #13. https://fisheries.org/policy-media/policy-statements/afs-policy-statement-13/

Stephenson, P. J. (2019). The Holy Grail of biodiversity conservation management: Monitoring impact in projects and project portfolios. *Perspectives in Ecology and Conservation*, *17*(4), 182–192. https://doi.org/10.1016/j.pecon.2019.11.003

Stets, E.G., Sprague, L.A., Oelsner, G.P., Johnson, H.M, Murphy, J.C., Ryberg, K., Vecchia, A.V., Zuellig, R.E., Falcone, J.A., and Riskin, M.L. 2020. Landscape drivers of dynamic change in water quality of U.S. rivers. Environmental Science and Technology, 54 (7), 4336-4343. DOI: 10.1021/acs.est.9b05344

Tarboton, D.G. 2003. A workbook to accompany the Rainfall-Runoff Processes Web module. Utah State University, Hydrology Research Group . <https://uwrl.usu.edu/hydrology/files/rrp/rainfall-runoff-processes.pdf>

The Nature Conservancy. 2020a. CONUS Landforms, based on terrestrial resilience plans 2016-2019. The Nature Conservancy, Center for Resilient Conservation Science. Newburyport, MA. Data download: https://tnc.app.box.com/file/959024468113?s=7dgi8gbc6w7e39qu14yjgwdix96aqoro; AGOL Map Service: <https://tiles.arcgis.com/tiles/F7DSX1DSNSiWmOqh/arcgis/rest/services/Landforms/MapServer>.

The Nature Conservancy. 2020b. TNC Terrestrial Ecoregion Boundaries. The Nature Conservancy, Center for Resilient Conservation Science. Newburyport, MA. Data download: https://tnc.app.box.com/s/nkph9u19ucy11uzuo961x9qjynbbdtcb; AGOL Map Service: <https://tnc.maps.arcgis.com/home/item.html?id=0316dce180e14f339181ac8d4986b4d5>.

The Nature Conservancy. 2020c. CONUS Geophysical Settings based on terrestrial resilience plans 2016-2019. The Nature Conservancy, Center for Resilient Conservation Science. Newburyport, MA. Data download: <https://tnc.app.box.com/file/959021593944?s=voj4tfz0dyokpvwrldji9b3nbrsi36xl>; AGOL Map Service: <https://tiles.arcgis.com/tiles/F7DSX1DSNSiWmOqh/arcgis/rest/services/Geophysical_Settings/MapServer>

The Nature Conservancy. 2024. Conservation Lands. Compiled from multiple sources. https://tnc.maps.arcgis.com/home/item.html?id=5686424360814955a7d40ce1c2442549

Theobald D.M., Harrison-Atlas D., Monahan W.B., Albano C.M. 2015. Ecologically-Relevant Maps of Landforms and Physiographic Diversity for Climate Adaptation Planning. PLoS ONE, 10(12): e0143619. doi:10.1371/journal.pone.0143619

Theobald, D. 2022. SRTM CHILI CONUS. 30-m grid dataset. Google Earth Engine Extract 5/18/2022.

Tickner, D., Opperman, J.J., Abell, R., Acreman, M., Arthington, A.H., Bunn, S.E., Cooke, S.J., Dalton, J., Darwall, W., Edwards, G., Harrison, I.A.N., Hughes, K., Jones, T.I.M., Leclère, D., Lynch, A.J., Leonard, P., Mcclain, M.E., Muruven, D., Olden, J.D., and Young, L. 2020. Bending the curve of global freshwater biodiversity loss: an emergency recovery plan. BioScience, 70(4):330–342. https://doi.org/10.1093/biosci/biaa002.

Timpane-Padgham, B. L., Beechie, T., & Klinger, T. 2017. A systematic review of ecological attributes that confer resilience to climate change in environmental restoration. PLoS ONE, 12(3), e0173812. https://doi.org/10.1371/journal.pone.0173812

Townsend P. A., Helmers D. P., Kingdon C. C., McNeil B. E., de Beurs K. M., Eshleman K. N. 2009. Changes in the extent of surface mining and reclamation in the Central Appalachians detected using a 1976–2006 Landsat time series. Remote Sensing of Environment, 113(1):62–72.

U.S. Energy Information Administration (EIA) and U.S. Department of Labor, Mine Safety and Health Administration. 2020. Coal Mines digital vector data. https://atlas.eia.gov/datasets/eia::coal-mines-1/about .

U.S. Environmental Protection Agency (USEPA). 2020. National Rivers and Streams Assessment 2013–2014: A Collaborative Survey. EPA 841-R-19-001. Washington, DC. https://www.epa.gov/national-aquatic-resource-surveys/nrsa

U.S. Geological Survey (USGS). 2003. Principal Aquifers of the 48 Conterminous United States, Hawaii, Puerto Rico, and the U.S. Virgin Islands. 1:2,500,000. http://nationalatlas.gov/atlasftp.html.

U.S. Geological Survey (USGS). 2005. Active Mines and Mineral Processing Plants in the United States in 2003: U.S. Geological Survey, Reston, Virginia. https://mrdata.usgs.gov/mineplant/

U.S. Geological Survey (USGS). 2016. HUC-12 Watershed Boundary Polygons, US and Territories, 2016, WBD.

Valayamkunnath, P., Barlage, M., Chen, F., Gochis, D.J., and Franz, K. J. 2020. Mapping of 30-meter resolution tile-drained croplands using a geospatial modeling approach. Scientific Data, 7:257. <https://doi.org/10.1038/s41597-020-00596-x>.

Vannote, R. L., G. W. Minshall, K. W. Cummins, J.R. Sedell, and E. Cushing. 1980. The river continuum concept. Canadian Journal of Fisheries and Aquatic Sciences, 37:130-137.

Wang, L., Infante, D., Esselman, P., Cooper, A., Wu, D., Taylor, W., Beard, D., Whelan, G. and Ostroff, A. 2011. A hierarchical spatial framework and database for the National River Fish Habitat Condition Assessment. Fisheries, 36(9):436-449, DOI: 10.1080/03632415.2011.607075.

Weary, D.J., and Doctor, D.H., 2014. Karst in the United States of America: a digital map compilation and database. U.S. Geological Survey Open-file Report 2014-1156. <https://pubs.usgs.gov/of/2014/1156/>

Wenger, S.J., C.H. Luce, A.F. Hamlet, D.J. Isaak, and H.M Neville. 2010. Macroscale hydrologic modeling of ecologically relevant flow metrics. Water Resources Research. 46: W09513. doi:10.1029/2009WR008839

Wilby, R. L. (2020). Change and Water Management. Water, 12, 2510. https://doi.org/10.3390/w12092510

Wise, D.R. 2020. SPARROW model inputs and simulated streamflow, nutrient and suspended-sediment loads in streams of the Pacific Region of the United States, 2012 Base Year (ver 1.1, June 2020): U.S. Geological Survey data release, https://doi.org/10.5066/P9AXLOSM.

Woodward, G., Perkins, D. M., & Brown, L. E. 2010. Climate change and freshwater ecosystems: Impacts across multiple levels of organization. Philosophical Transactions of the Royal Society B: Biological Sciences, 365(1549):2093–2106. <https://doi.org/10.1098/rstb.2010.0055>

Wolock, D. M. 2003. Base-flow index grid for the conterminous United States. U.S. Geological Survey Open-File Report 03-263. U.S. Geological Survey. Reston, Virginia. <https://onlinelibrary.wiley.com/r/eap1of19https://doi.org/10.1002/eap.2534>

Wolock, D.M. and McCabe, G.J. 2017. USGS Water Balance Tabular Dataset. <https://data.usgs.gov/datacatalog/search?otherKeyword=%5B%22water+balance+model%22%5D>

Wolock, D. M., Winter, T. C. and McMahon, G. 2004. Delineation and evaluation of hydrologic landscape regions in the United States using geographic information system tools and multivariate statistical analyses. Environmental Management, 34:S71-S88.
